# IRF8-mutant B cell lymphoma evades immunity through a CD74-dependent deregulation of antigen processing and presentation in MHCII complexes

**DOI:** 10.1126/sciadv.adk2091

**Published:** 2024-07-12

**Authors:** Zhijun Qiu, Jihane Khalife, Purushoth Ethiraj, Carine Jaafar, An-Ping Lin, Kenneth N. Holder, Jacob P. Ritter, Lilly Chiou, Gabriela Huelgas-Morales, Sadia Aslam, Zhao Zhang, Zhijie Liu, Shailee Arya, Yogesh K. Gupta, Patricia L. M. Dahia, Ricardo C.T. Aguiar

**Affiliations:** ^1^ Division of Hematology and Medical Oncology, Department of Medicine, Mays Cancer Center, University of Texas Health Science Center San Antonio, San Antonio, TX 78229, USA.; ^2^ Department of Pathology, University of Texas Health Science Center San Antonio, San Antonio, TX 78229, USA.; ^3^ Department of Molecular Medicine, University of Texas Health Science Center San Antonio, San Antonio, TX 78229, USA.; ^4^ Department of Biochemistry and Structural Biology, University of Texas Health Science Center San Antonio, San Antonio, TX 78229, USA.; ^5^ South Texas Veterans Health Care System, Audie Murphy VA Hospital, San Antonio, TX 78229, USA.

## Abstract

The mechanism by which interferon regulatory factor 8 (IRF8) mutation contributes to lymphomagenesis is unknown. We modeled IRF8 variants in B cell lymphomas and found that they affected the expression of regulators of antigen presentation. Expression of IRF8 mutants in murine B cell lymphomas suppressed CD4, but not CD8, activation elicited by antigen presentation and downmodulated CD74 and human leukocyte antigen (HLA) DM, intracellular regulators of antigen peptide processing/loading in the major histocompatibility complex (MHC) II. Concordantly, mutant IRF8 bound less efficiently to the promoters of these genes. Mice harboring IRF8 mutant lymphomas displayed higher tumor burden and remodeling of the tumor microenvironment, typified by depletion of CD4, CD8, and natural killer cells, increase in regulatory T cells and T follicular helper cells. Deconvolution of bulk RNA sequencing data from IRF8-mutant human diffuse large B cell lymphoma (DLBCL) recapitulated part of the immune remodeling detected in mice. We concluded that IRF8 mutations contribute to DLBCL biology by facilitating immune escape.

## Teaser

IRF8 mutations contribute to lymphoma pathogenesis by promoting immune escape.

## INTRODUCTION

The immune system can detect early signs of, and eliminate, malignant cell transformation. To thrive, cancer cells acquire genetic changes that can disengage this surveillance system (*1*, *2*). Pharmacological strategies that reestablish antitumor immunity have cemented the pivotal role of the immune system in cancer biology (*3*).

In diffuse large B cell lymphoma (DLBCL), a common, genetically diverse, and still often fatal B cell malignancy, the mutations that disarm the immune system range from highly predictable suspects, including genes encoding products directly involved in antigen processing/presentation (e.g., *B2M*, *CIITA*, and *HLA-A/B/C*) (*4*–*6*), to less immediately obvious candidates, such as chromatin modifiers (e.g., *CREBBP* and *EZH2*) (*7*–*9*).

The genetic heterogeneity of DLBCL is well recognized, and more than 100 protein-coding genes are recurrently mutated or targeted by somatic structural changes in this lymphoma type (*10*, *11*). The current classification systems identify approximately six DLBCL subgroups driven by distinct biological processes (*12*, *13*). In multiple instances, the mechanism by which a given driver gene contributes to DLBCL development has been, at least partially, elucidated (*14*). However, in the case of interferon regulatory factor 8 (IRF8), which is a common target for somatic mutations in DLBCL, it remains unclear how these variants may contribute to lymphomagenesis. Notably, IRF8 is also deregulated by series of potentially oncogenic events, including chromosomal translocation (*15*), focal amplification (*12*, *13*), and super-enhancer perturbations (*16*, *17*), in principle suggesting that the mutations may also represented gain-of-function events.

IRF8, a member of the IRF family of transcription factor, plays important roles in both myeloid differentiation and B cell development (*18*, *19*). IRF8 binds to specific DNA sequences as a heterodimer with partner transcription factors and can act as either an activator or a repressor. In mice, germline deletion of *Irf8* yields a dominant myeloid phenotype (a chronic myeloid leukemia-like disease), which agrees with Irf8’s essential role on monocyte and dendritic cell (DC) development (*20*). Concordantly, in humans, germline loss-of-function *IRF8* missense mutations cause rare, at times severe, inborn errors of immunity, characterized by susceptibility to infections, depletion of DCs, and reduced numbers and/or impaired activity of natural killer (NK) cells (*21*–*24*).

IRF8’s contribution to B cell physiology is more nuanced. IRF8 is expressed in multiple B cell developmental stages, predominates in the germinal center, and is absent in plasma cells (*18*, *25*). IRF8 regulates several genes important for normal and malignant B cell biology (e.g., *BCL6* and *AICDA*) (*26*), but deletion of *Irf8* in the mouse B cell compartment yielded only a modest phenotype (*27*), which could be better appreciated in mice with B cell–specific double *Irf8*/*Spi1* (encoding PU.1) knockout (KO). In these instances, Irf8 was shown to be important for the development of follicular B cells and germinal center responses and to constrain plasma cell differentiation (*28*, *29*). However, many of the validated IRF8 targets are known to be important for antigen processing/presentation (*30*–*33*), raising the possibility that IRF8 may also play a role in the immune composition of the tumor microenvironment (TME), while its dysfunction could facilitate immune escape.

Here, we show that *IRF8* missense or nonsense mutants, mapping either to the N-terminal DNA binding domain (DBD) or C-terminal tail, display impaired promoter binding and decreased expression of CD74 and human leukocyte antigen DM (HLA-DM), intracellular modulators of antigen processing, and loading into the major histocompatibility complex II (MHCII) complex. Concordantly, we find that *IRF8* mutations in primary human DLBCLs are mutually exclusive with mutations in genes directly associated with antigen presentation including *CIITA*, a direct IRF8 target and master regulator of de novo transcription of MHCII complex genes (*33*–*35*), as well as *HLA-DMB* and *CD74.* In IRF8-mutant lymphoma models, consistent downmodulation of CD74 and HLA-DM significantly deregulated antigen presentation in an MHCII but not MHCI context (*34*). Mice harboring IRF8-mutant lymphomas displayed larger tumor burden, in association with remodeling of the TME, an immunophenotype that was rescued in vivo by ectopic expression of CD74 and validated in primary human DLBCLs. We concluded that IRF8 mutations are functionally deficient and contribute to DLBCL biology by facilitating immune evasion. Together with the earlier evidence of oncogenic aberrations targeting its locus (*15*–*17*), we propose that IRF8’s role in DLBCL is uniquely multilayered and that both its loss and gain of function may be lymphomagenic.

## RESULTS

### Landscape of IRF8 mutations in DLBCL

**Fig. 1.**
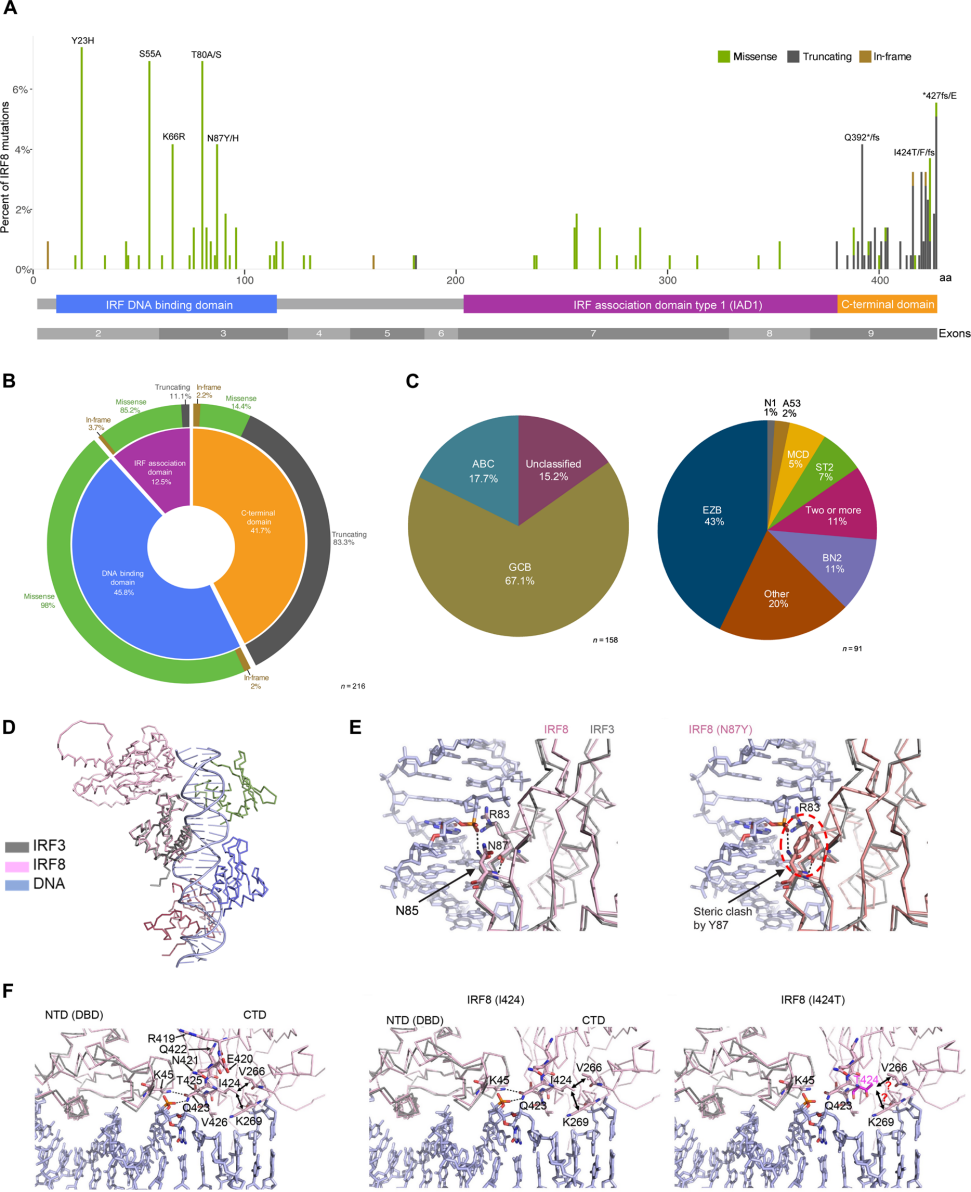
*IRF8* mutations in DLBCL. (**A**) Diagram of 216 *IRF8* gene variants reported in publicly available DLBCL cohorts distributed along the linear protein and its recognized domains; the frequency of occurrence (%) of the variants is indicated by the height of the line, and the type of mutation is color coded based on the mutation class, related to tables S1 to S3. (**B**) Donut-shape graph showing the distribution (%) of the 216 *IRF8* variants shown in (A) based on the mutation class and functional protein domains, related to table S2. (**C**) Distribution of *IRF8* variants based on transcription (left, ABC, GCB, or unclassified; *n* = 158) and genetic-based DLBCL subgroups (right, EZB, BN2, A53, MCD, ST2, N1, other, or two or more groups; *n* = 91), related to table S4. (**D**) Overlay of the DBDs of IRF3 (gray) and IRF8 (pink). DNA-bound to IRF3 DBD is shown in light blue. (**E**) Left: Close-up of the N87 residue in the IRF8 DBD in contact with DNA; corresponding IRF3’s N85 in is also shown. Right: Steric clash by Y87 side chain, highlighted by red circle. (**F**) Left to right: C-terminal tail residues (R419 to V426) may mediate electrostatic interactions with DNA and DBD and are sandwiched between the DBD and CTD. Middle: I424 side chain faces the interior of CTD and may stabilize it via interactions with V266 and K269, and the polar side chain of Q423 may form H bonds to K45 from DBD and the phosphoryl oxygens of the DNA backbone. Right: The polar side chain of mutant T424 may disrupt the hydrophobic interior of CTD and displace Q423 from its original position, resulting in a loss of interaction with DBD and DNA.

Approximately 8% of DLBCLs harbor *IRF8* mutation [*n* = 244 from 3187 previously reported DLBCL sequences; table S1 (*7*, *12*, *13*, *36*–*41*)]. The mutations cluster into two recognized IRF8 domains, DNA binding and protein interaction domains [IRF association domain (IAD)] but also in a less well-defined C-terminal region of the protein. Missense variants predominate in the DNA binding and IAD, whereas most of the mutation in the C-terminal tail are truncating (Fig. 1, A and B, and table S2). In all instances in which variant allele frequency could be defined, the *IRF8* mutations were found to be monoallelic (table S3). Most of the IRF8-mutant DLBCLs are of the germinal center B-cell (GCB)-like subtype and EZB category (Fig. 1C and table S4). IRF8 is also frequently mutant in follicular lymphoma (*42*, *43*), Burkitt lymphoma (*44*, *45*) and to lesser extent in marginal zone lymphoma (*46*) (fig. S1 and table S5), all with a mutation pattern similar to that found in DLBCL (table S6). To gain initial insights into the putative mechanisms by which IRF8 mutations may deregulate protein function, we performed a comparative structural analysis using experimental structures of the DBD of the homologous IRF3 [Protein Data Bank (PDB): 2PI0] bound to a double-stranded DNA (*47*) (fig. S2). No experimental structure is yet available for partial or full-length IRF8; thus, we used a three-dimensional model of IRF8 as predicted by AlphaFold (*48*) . The AlphaFold model shows a bilobal architecture of IRF8 with its N-terminal DNA binding (DBD; amino acids 1 to 116) connected via a flexible linker (amino acids 117 to 169) to the “C-terminal domain” (CTD), which is composed of a well-defined protein-protein interaction (amino acids 170 to 382) and a still uncharacterized C-terminal region (amino acids 383 to 426) (fig. S2). Next, we overlayed the IRF3 and IRF8 DBDs (Fig. 1D), which enabled us to examine IRF8’s protein-protein and protein-DNA interactions in detail and model the putative mechanism of protein dysfunction associated with the mutations (S55A, N87Y, Q392X, D400G, and I424T) that we characterized in vitro and in vivo (see below). Upon superimposing the IRF3-DNA structure with the IRF8 model, we found that the polar side chain of N87 is within the H-bonding distance of the DNA backbone, akin to N85, the equivalent residue in IRF3. A bulkier and hydrophobic side chain of N87Y mutant will lose this critical H bond and electrostatic interaction with DNA. Moreover, a Y at position 87 can sterically clash with the side chain of R83, an amino acid required for charge-charge interaction with DNA (Fig. 1E). Another DBD mutation that we studied in vitro is S55A; S55 locates within the α2 helix of the IRF8 DBD (fig. S3). Its side chain stabilizes the loop1 between α2 and β1. This loop enters the minor groove of DNA. An alanine at position 55 may result in loss of this stabilizing interaction and negatively affect DNA binding (fig. S3). The CTD of IRF8 is predicted to primarily promote interaction with other proteins (*49*). However, the AlphaFold model of the full-length IRF8, and its comparative analyses with the DNA-bound IRF3 structure, revealed the orientation of IRF8’s CTD close to the upstream portion of DNA and suggested that the very C-terminal tail (amino acids 419 to 426) sandwiches between DBD and CTD, thus acting as a hinge to stabilize the two domains and bring the CTD close to DNA (Fig. 1F). Close examination of I424 showed that its side chain faces the interior of CTD to stabilize it via hydrophobic interactions with V266 and the aliphatic portion of the side chain of K269 (Fig. 1F). In addition, the polar side chain of Q423 faces outward to form bidentate H bonds to K45 from DBD and the phosphoryl oxygens of the DNA backbone. We predict that the polar side chain of the I424T mutant that we examined in vitro and in vivo will disrupt the hydrophobic interior of CTD. As a result, Q423 may be displaced from its original position, resulting in a loss of interaction with DBD and DNA. Modeling of the truncated IRF8 Q392X, tested in vitro and in vivo (see below), shows that it may lose potential DNA- and interdomain-stabilizing contacts mediated by the C-terminal tail (fig. S4). The final missense CTD mutation that we examined in vitro is D400G. An overlay of the AlphaFold IRF8 model and IRF3 CTDs reveals that D400 lies close to the putative protein-protein interface of IRF8, in an “outer loop”; a G at position 400 may influence the flexibility of this region affecting the potential protein-protein interaction (fig. S5).

### Impact of IRF8 mutations on DLBCL fitness

**Fig. 2.**
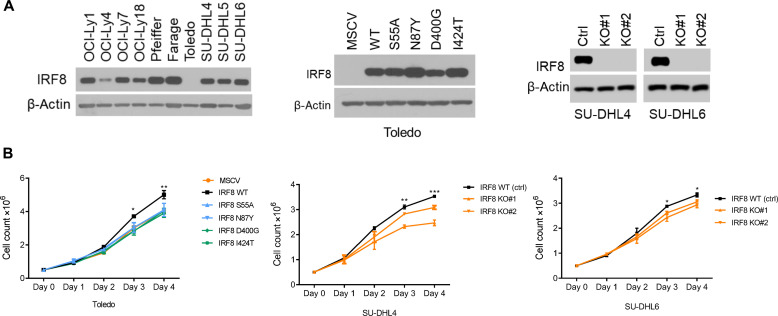
IRF8 influence on DLBCL growth. (**A**) Left to right: Western blot analyses of IRF8 expression in parental DLBCL cell lines, in the Toledo cell line stably expressing and empty vector (MSCV), IRF8 WT or four mutant isoforms (middle), and in two DLBCL cell lines with CRISPR-Cas9–based KO of IRF8. (**B**) Left to right: Cell growth pattern, determined with automated fluorescent cell counter, for the cell models described in (A). Data are means ± SD of three biological replicates. *P* values, WT versus each mutant or WT versus KO were calculated with two-sided Student’s *t* test, *P < 0.05, **P < 0.01.

Earlier, we reported on a small subset of DLBCL with a t(14;16) (q32;q24) juxtaposing the 5′ regulatory regions of the *IRF8* gene to immunoglobulin heavy chain (IgH) enhancers (Eα and Eμ) (*15*). We confirmed that this archetypal gain of function rearrangement resulted in elevated IRF8 expression in primary DLBCL, which in turn conferred a survival benefit to DLBCL cell line models, a finding validated by others (*50*). A putative oncogenic *IRF8* super-enhancer has also been reported (*16*, *17*). Therefore, we first examined whether the IRF8 mutations could enhance DLBCL fitness. To generate suitable models for downstream examination of IRF8 mutations, we screened a panel of DLBCL cell lines and found that Toledo did not express IRF8. Therefore, we created Toledo cells expressing IRF8 wild type (WT) or a series of frequent mutant variants, which map to distinct IRF8 domains (S55A, N87Y, D400G, and I424T) (Fig. 2A). In addition, we generated IRF8 KO models in two additional DLBCL cell lines, SU-DHL4 and SU-DHL6 (Fig. 2A). We confirmed that these DLBCL models express PU.1, an important component of the IRF8 functional complex (fig. S6) (*51*). We next tested whether genetic modulation of IRF8-modified DLBCL growth. In comparison to WT IRF8, expression of each IRF8 mutant examined negatively affected DLBCL growth (Fig. 2B). In addition, in agreement with earlier evidence for the growth promoting effect of WT IRF8 overexpression (*15*), we found that IRF8 KO limited DLBCL growth (Fig. 2B). We concluded that in the DLBCL models tested, IRF8 mutations did not stimulate cell growth and were more akin to KO models, which led us to consider the possibility that these variants may represent a cancer cell intrinsic defect that primarily influences the lymphoma microenvironment.

### Impact of IRF8 mutations on its transcriptional activity

**Fig. 3.**
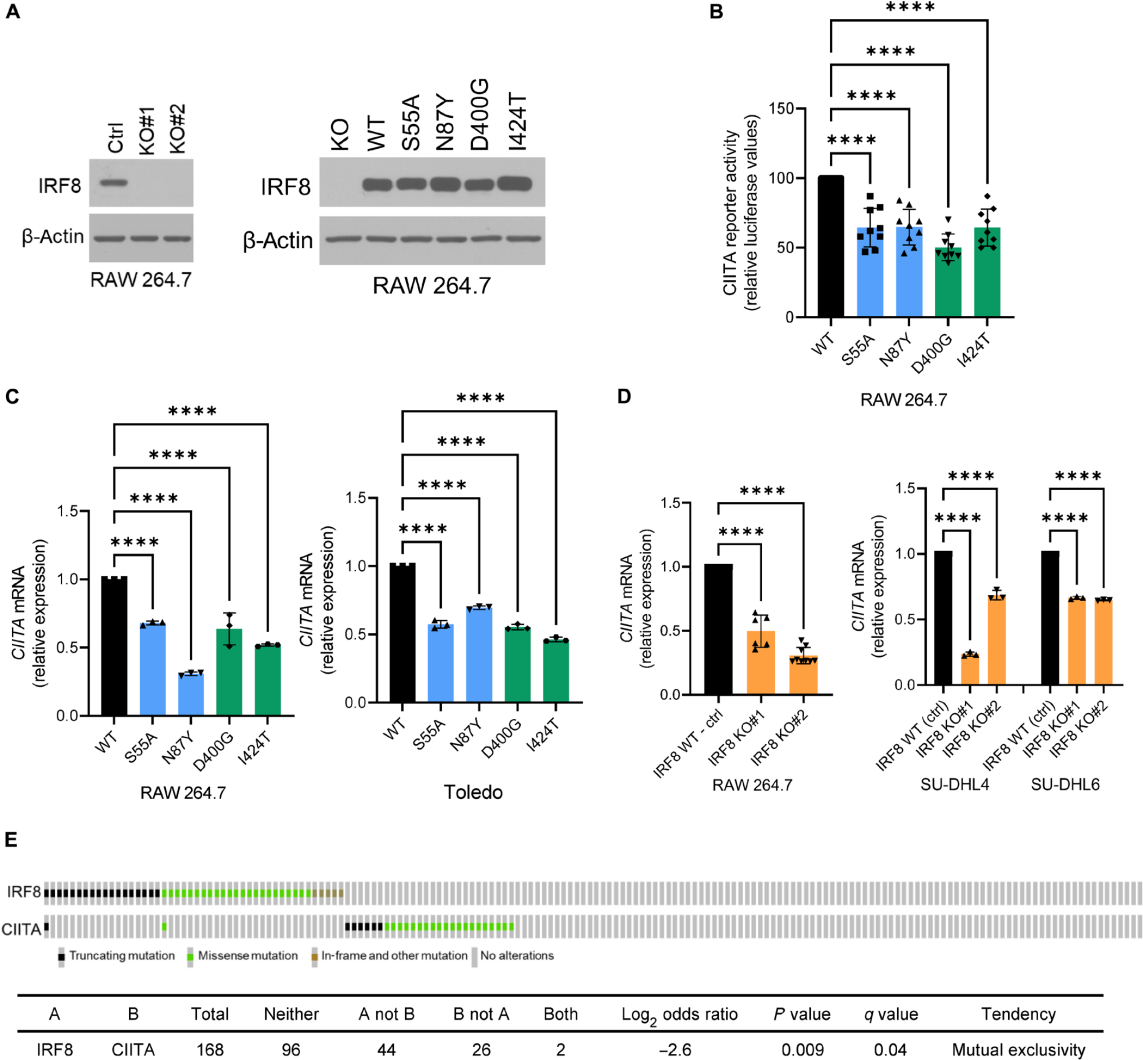
IRF8 modulation of CIITA. (**A**) Left to right: Western blot analyses of IRF8 expression in RAW 264.7 with IRF8 KO or stably expressing IRF8 WT and four mutant isoforms. (**B**) Relative luciferase CIITA reporter activity in RAW 264.7 cells expressing IRF8 WT or mutant. Data are means ± SD of three biological replicates each performed in technical triplicates (all nine data points shown). Statistical analysis is from one-way analysis of variance (ANOVA) with Bonferroni posttest, ****P < 0.0001. (**C**) Relative *CIITA* mRNA levels in RAW 264.7 and Toledo models expressing IRF8 WT or mutant. (**D**) Relative *CIITA* mRNA levels in RAW 264.7, SU-DHL4 and SU-DHL6 models of IRF8 KO. In (C) and (D), data are means ± SD. Statistical analyses are from one-way ANOVA with Bonferroni posttest, ****P < 0.0001. All assays performed in two to three biological replicates, each with technical triplicates. (**E**) Oncoprint display of comparative distribution of *IRF8* and *CIITA* gene mutations in DLBCLs; total number of samples, mutations distribution, pairwise log2 odds score, and P and Q values of the correlation between the co-occurrence (positive score) or mutually exclusive (negative score) are shown in table format. Symbols are color coded based on the mutation class.

IRF8 regulates a broad array of genes involved in antigen presentation, including CIITA (*30*–*33*, *51*), a transcriptional coactivator and master regulator of MHCII gene expression (*35*), and consequently antigen processing, loading, and display on the cell surface (*34*). To examine the impact of the IRF8 mutations toward this target, we first created IRF8 KO and “rescue” models (human IRF8 WT, S55A, N87Y, D400G, and I424T) in the RAW 264.7 murine macrophage cell line, an interferon-γ (IFN-γ)–responsive model, often used to functionally investigate the IRF proteins (*52*) (Fig. 3A). These stable cell models were transfected with CIITA WT promoter or a CIITA construct with point mutations in the Ets-IRF composite element (EICE) site (a gift from K. Wright, Moffit Cancer Center), which has been previously shown to abrogate the recruitment of the IRF8/PU.1 complex to DNA (*51*), and their luciferase activity was measured. We found that the CIITA reporter activity was significantly lower in cells expressing any of the IRF8 mutants in comparison to IRF8 WT (Fig. 3B). In agreement with the reporter assay data, *CIITA* expression was significantly higher in IRF8 WT than in IRF8-mutant RAW 264.7 and human DLBCL Toledo cells (Fig. 3C and fig. S7), or in IRF8 WT versus KO in RAW 264.7, SU-DHL4, and SU-DHL6 cells (Fig. 3D), findings confirmed at protein level (fig. S8). We then examined a cohort of primary human DLBCL (*n* = 168) and *IRF8* and *CIITA* mutations were found to be mutually exclusive (Fig. 3E). *IRF8* mutations in DLBCL were mutually exclusive with mutation in a series of genes that are directly (*CIITA*, *CD74*, *HLA-DMB*, *B2M*, *HLA-B*, and *HLA-C*) or indirectly (*CREBBP* and *EP300*) involved with antigen presentation and/or remodeling of the TME (fig. S9); mutations in *IRF8* and *HLA-A*, *HLA-DMA*, or *EZH2*, also had a mutually exclusive tendency, although not meeting statistical significance (fig. S9). We caution that variants in *CD74* and *HLA-DM* are uncommon in DLBCL and remain to be functionally characterized. We concluded that similarly to *CIITA*, *EZH2*, and *CREBBP* mutations (*6*–*8*), IRF8 variants may add to the pathogenesis of B cell lymphomas by impairing antigen presentation in the context of MHCII.

### Effects of IRF8 mutations on antigen presentation

**Fig. 4.**
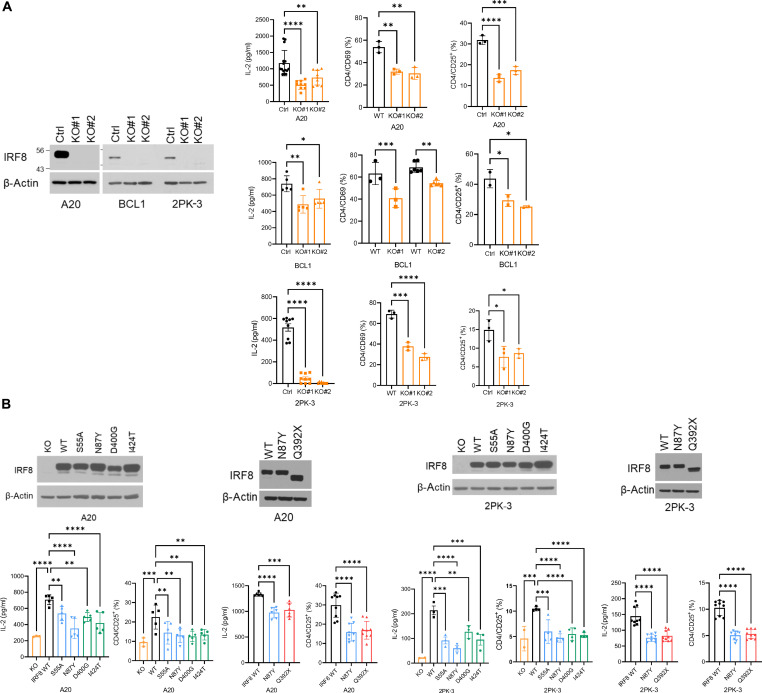
IRF8 impact on antigen driven CD4 activation. (**A**) Left: Western blot of IRF8 KO models in mouse cell lines A20, BCL1, and 2PK-3; ctrl is empty vector. Right: (left to right) IL-2 levels in the conditioned media and percentage of CD4/CD69 and CD4/CD25^+^ expression in DO-11.10 cells cocultured with IRF8 WT or KO antigen-presenting cell (APC) models. (**B**) Top: Western blot of IRF8 A20 (left) or 2PK-3 (right) KO models “rescued” with stable expression of IRF8 WT or mutant. Bottom: IL-2 levels in the conditioned media and percentage of CD4/CD25^+^ expression in DO-11.10 cells cocultured with IRF8 KO, WT and missense (left) or nonsense (right) mutant models. In (A) and (B), data are means ± SD of three biological replicates. *P* values are from one-way ANOVA, Bonferroni or Fisher’s LSD posttest, *P < 0.05, **P < 0.01, ***P < 0.001, ****P < 0.0001.

To test the idea that IRF8’s role in DLBCL biology may be related to a defective antigen processing/presentation, we first created IRF8 KO models in three murine B cell lymphoma cell lines—A20, 2PK-3, and BCL1 (Fig. 4A). Subsequently, we used DO-11.10 murine CD4 cells [a T cell hybridoma specific for chicken ovalbumin (OVA), a gift from P. Marrack, National Jewish, Denver, CO, and J. Maynard, UT Austin, Austin, TX] to test the ability of IRF8 WT or KO lymphoma cells to present antigen and, in the context of MHCII, activate CD4 T cells. IRF8 KO cells were significantly less capable of eliciting a robust CD4 response to OVA presentation, as defined by interleukin-2 (IL-2) secretion, and CD69 or CD25 cell surface expression in CD4 cells (Fig. 4A and figs. S10 and S11). These effects were not detected in the absence of OVA (or B lymphoma cells), mitigating concerns about a putative effect of IRF8 KO lymphoma cells on T cell activation outside the antigen presentation axis (fig. S12). These data also confirmed, and expanded on, earlier reports demonstrating the ability of the A20 murine B cell lymphoma cells to effectively present antigen and elicit an immune response (*53*, *54*). To establish the scope of defect in antigen presentation associated with IRF8 KO, we also tested the MHCI-CD8 axis. Here, we isolated T cells from the OT-I T cell receptor (TCR) transgenic mice, which recognizes OVA residues 257 to 264 in a MHCI-restricted context (*55*), and quantified the resulting CD8 activation/differentiation in vitro by measuring their cytotoxicity toward isogenic IRF8-WT or KO murine B cell lymphoma cells. OVA presentation by the lymphoma cells readily activated the OT-I CD8 cells, as measured by secretion of granzyme B and CD8-mediated cytotoxicity (fig. S13). However, contrary to the differential CD4 activation, isogenic IRF8 WT or KO lymphoma cells equally induced CD8-mediated cytotoxicity (fig. S13). Next, we rescued IRF8 expression (WT, S55A, N87Y, Q392X, D400G, and I424T) in the IRF8 KO A20 and 2PK-3 B cell lymphoma models (Fig. 4B) and used the DO-11.10 CD4 assay to test the impact of IRF8 mutations on antigen presentation/T cell activation (here, no analysis of CD8 OT-I cells was warranted given that IRF8 KO did not affect CD8 activation). We consistently found that following OVA loading, A20 or 2PK-3 expressing IRF8 WT induced a significantly more robust CD4 activation, measured by IL-2 secretion and CD25 expression, than each of the IRF8 missense or truncating mutants (Fig. 4B and fig. S14). Notably, IRF8 residues targeted by missense mutations (S55, N87, D400, and I424) are fully conserved between human and mouse, increasing confidence on the correlation between output data from either model system. We concluded that in murine models of B cell lymphomas examined in vitro, IRF8 regulates antigen OVA-driven T cell activation in the context of MHCII but not MHCI.

### Mediators of IRF8 effects on antigen processing/presentation

**Fig. 5.**
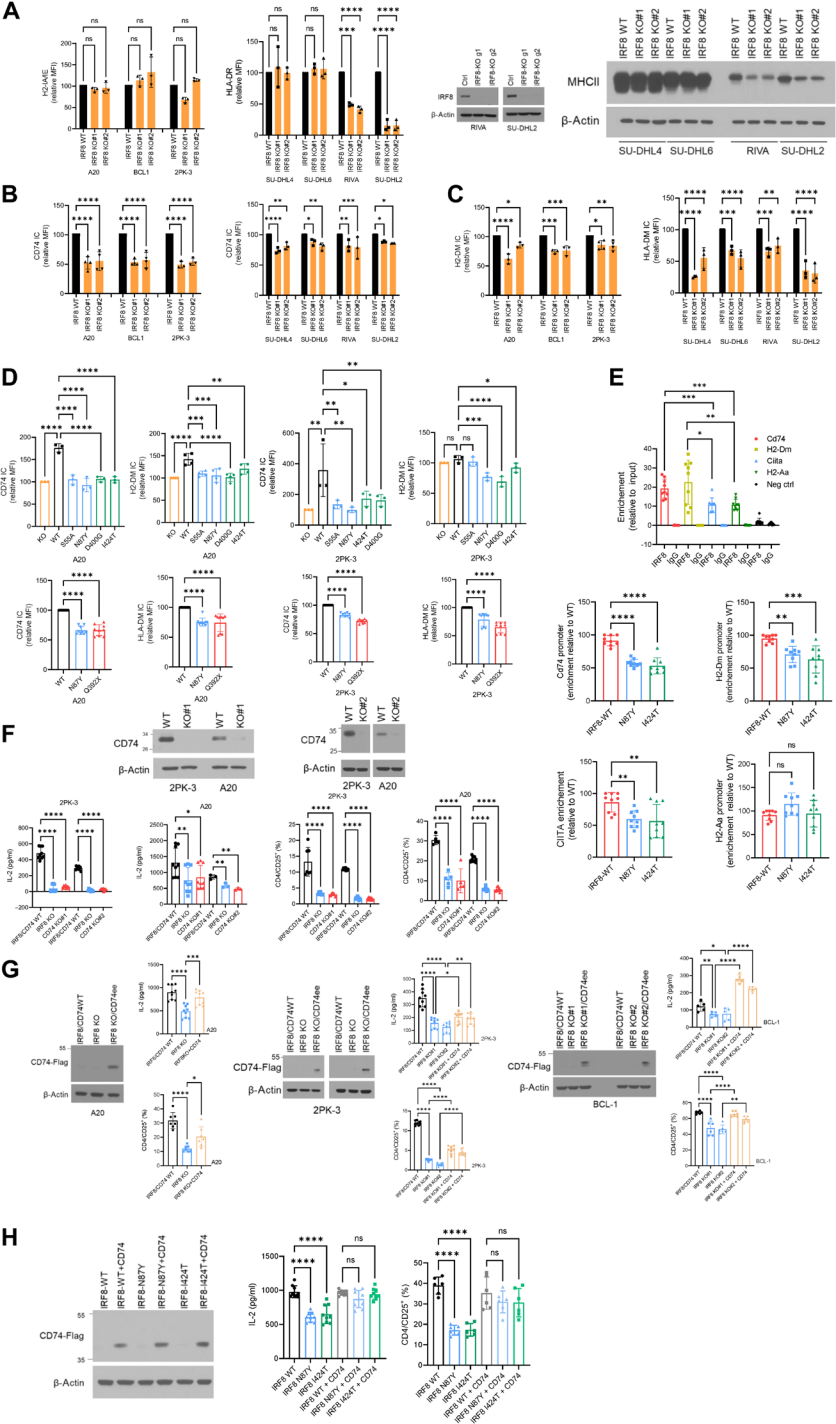
IRF8 control of components of the MHCII complex. (**A**) FACS analysis of H2-IA/IE (left) and HLA-DR (right) in models of IRF8 KO; WB of IRF8 in RIVA and SU-DHL2 KO models, and WB of MHCII in all IRF8 KO models in DLBCL. (**B**) FACS of CD74 in models of IRF8 KO. (**C**) FACS of H2-DM and HLA-DM in IRF8 KO models. (**D**) Left: FACS of CD74 and H2-DM in the IRF8 KO A20 lymphoma model “rescued” with IRF8 WT or missense or nonsense mutants (top and bottom). Right: FACS of CD74 and H2-DM in the IRF8 KO 2PK-3 lymphoma model “rescued” with IRF8 WT or missense and nonsense mutants (top and bottom). (**E**) Top: ChIP-qPCR of IRF8 binding to the indicated promoters – controls are IgG pull down, and a genomic region without a predicted IRF8 binding site (neg ctrl). Bottom: ChIP-qPCR of IRF8 WT, N87Y, or I424T binding to the *Cd74*, *H2-Dm*, *Ciita*, or *H2-Aa* promoters. (**F**) Top: WB of CD74 in 2PK-3 and A20 CD74-KO models. Bottom: IL-2 levels and % of CD4/CD25^+^ cells in IRF8/CD74 WT, IRF8 KO, or CD74 KOs models. (**G**) Left to right: A20, 2PK-3, and BCL1 models of IRF8 KO with CD74 ectopic expression (ee). WB of CD74-FLAG, IL-2 levels and % of CD4/CD25^+^ cells in IRF8/CD74 WT, IRF8 KO, or IRF8KO + CD74. (**H**) Left: WB of CD74-FLAG in IRF8 WT, N87Y, and I424T A20 models. Right: IL-2 levels and % of CD4/CD25^+^ cells in IRF8 WT, N87Y, and I424T (−/+ CD74 ectopic expression) models. Data are means ± SD of three biological replicates. FACS displayed as relative mean fluorescence intensity (MFI). *P* values are from ANOVA, with Bonferroni or Fisher’s LSD posttest, or two-sided Student’s *t* test. *P < 0.05, **P < 0.01, ***P < 0.001, ****P < 0.0001.

The defective activation of CD4 (but not CD8) T cells by IRF8-mutant B cell lymphomas, together with the well-recognized role of MHCII deficiency in DLBCL biology (*56*), suggested that IRF8 variants may deregulate genes and processes that are involved in antigen loading/processing and/or presentation. To examine this possibility, we first expanded the panel of DLBCL models and generated IRF8 KO models in two additional activated B-cell (ABC)-like cell lines, RIVA (RI-1) and SU-DHL2 (Fig. 5A). Similar to the findings in SU-DHL4 and SU-DHL6 (Fig. 2B), we found that IRF8 KO had only a modest impact on the growth of these DLBCLs (fig. S15). To probe deeper into the mechanistic basis for the potential impact of IRF8 on antigen presentation, we next measured the expression of HLA-DR (mouse H-2 IA/IE) in these multiple models. We found that in most instances, MHCII expression on the cell surface was unchanged by IRF8 KO, except for in the cell lines RIVA and SU-DHL2, which also express baseline HLA-DR at much lower levels (Fig. 5A). As the IRF8-deficient A20, BCL1, and 2PK-3 cells were defective in activating CD4 cells (Fig. 4, A and B), we considered the possibility that CD74 (Ia antigen-associated invariant chain, Ii) and/or HLA-DM, critical intracellular regulators of antigen processing/loading in a MHCII context, which are also transcriptional targets of both CIITA and IRF8 (*30*, *32*, *33*, *57*), may be modified by IRF8 KO or mutation. Using intracellular fluorescence-activated cell sorting (FACS) analyses, we detected a significant suppression of CD74 and HLA-DM (H2-DM) in all seven IRF8 KO models that we created (Fig. 5, B and C, and fig. S16). In the mouse models, the effects of IRF8 were more robust toward CD74, whereas in human cells, HLA-DM suppression was more marked. Then, we queried the impact of IRF8 WT or mutant expression on CD74 and H2-DM levels. In the A20 and 2PK-3 mouse models, the expression of CD74, as well as H2-DM was significantly higher in IRF8 WT than in IRF8 missense or nonsense mutant–expressing cells (Fig. 5D and fig. S17). Likewise, in the IRF8-null Toledo DLBCL cell line, the expression of CD74 and HLA-DM was significantly higher in IRF8 WT than mutant cells (fig. S18). The effects of IRF8 on CD74 and HLA-DM expression suggest that in IRF8-mutant tumors, correct antigen loading in the MHCII complex may be impaired in at least two nodes. In brief, the abnormally lower expression of CD74/invariant chain (Ii), which physiologically associates with MHCII αβ dimers, could result in incorrect loading of endogenously derived peptides at the endoplasmic reticulum. Through sequential proteolysis of the remaining li in IRF8-mutant cells, the CLIP (class II–associated invariant chain peptide) fragment is generated and remains bound to MHCII (to prevent premature peptide loading), until it is dislodged by HLA-DM allowing for proper binding of mature antigen [reviewed in (*34*)]. Thus, the lower abundance of HLA-DM in IRF8-mutant cells may inefficiently dislodge CLIP and blunt the loading of the correct antigen into the mature MHCII for cell surface presentation. If this latter assumption is correct, then in IRF8 KO or mutant cells, there will be more MHCII-CLIP on the cell surface. Using FACS, we validated this prediction in two human DLBCL models (SU-DHL4 IRF8 KO, Toledo IRF8 WT, and mutants) with detectable CLIP levels on the cell surface (fig. S19). We note that an antibody that detects CLIP in mouse cells is not available, so these quantifications could not be extended to the IRF8-modified murine B cell lymphoma models. All changes in expression denoted above were also captured at mRNA levels (fig. S20). We concluded that in B cell lymphoma models, IRF8 KO and/or expression of mutant variants decrease CD74 and HLA-DM expression, mainly deregulating intracellular antigen processing and loading, rather than MHCII expression on the cell surface.

Although *CD74*, *HLA-DM*, *HLA-DR*, and *CIITA* have all been reported as direct targets of IRF8 (*30*–*33*, *51*), an observation that we validated preliminarily by exploring publicly available Chromatin immunoprecipitation sequencing (ChIP-seq) data (figs. S21 and S22), we sought to confirm these earlier data by specific ChIP–quantitative polymerase chain reaction (qPCR) and, in the process, attempted to gain additional insight on the impact of IRF8 mutations on its binding to target promoters. We started by mining the ReMap22 resource (https://remap.univ-amu.fr/) in the University of California Santa Cruz (UCSC) genome browser to map putative IRF8 binding sites to the promoters of these four genes. Manual curation allowed us to identity canonical IRF8 binding sites in all promoters. Next, using ChIP-qPCR, we confirmed that IRF8 WT binds to promoters of all investigated genes but found that the binding was significantly stronger to the regulatory regions of *Cd74* and *H2-Dm* than of *Ciita* and *H2-Aa* (Fig. 5E). Capitalizing on the isogenic models of B cell lymphomas expressing IRF8 WT, N87Y, and I424T that we developed, we then tested whether these IRF8 mutants lose their promoter binding capacity. We found that in comparison to IRF8 WT, the mutants displayed significantly less efficient binding to *Cd74*, *H2-Dm*, and *Ciita* but not to the *H2-Aa p*romoter (Fig. 5E). These data agree with the more uniform and pronounced suppression of CD74 and HLA-DM found in IRF8-mutant B cell lymphomas. At the moment, it is not clear why the binding of IRF8 mutants to the *H2-Aa* promoter was not significantly impaired, nor why IRF8-associated modulation of CIITA did not consistently impair MHCII expression on the cell surface.

In murine B cell lymphoma models, which allow for in vitro (CD4 DO-11.10 cells) and in vivo functional examination of defective antigen presentation, CD74 appear to be the main “target” of IRF8 effects (Fig. 5, B to E). Thus, to validate the relevance of this interplay, we used CRISPR-Cas9, and two distinct guide RNAs, to KO CD74 expression in the A20 and 2PK-3 B cell lymphoma models and found that similarly to loss of IRF8, KO of CD74 resulted in significantly diminished CD4 activation (Fig. 5F and fig. S23). These data reinforce the essentiality of Ii in preventing premature loading of antigen peptide into the MHCII complex toward proper cell surface presentation (*34*) and eventual CD4 T cell activation and start to point to a mechanism by which IRF8 dysfunction may facilitate immune escape. Next, we ectopically expressed mouse Cd74 (p41 isoform) in the IRF8 KO A20, 2PK-3, and BCL1 B cell lymphoma models and showed that this genetic modulation was sufficient to rescue the defective OVA-elicited CD4 activation (IL-2 secretion and CD25 expression) associated with IRF8 KO (Fig. 5G and fig. S24). Last, to mitigate concerns that the ectopic expression of CD74 may enhance CD4 activation irrespective of IRF8 status, and to define whether CD74 can rescue the IRF8-mutant phenotype, we tested CD74 effects on B cell lymphoma models expressing IRF8 WT, the DBD mutant N87Y, or the CTD mutant I424T. Reassuringly, we found that ectopic CD74 expression significantly increased OVA-driven CD4 activation in IRF8 N87Y and I424T, but it had a negligible effect on IRF8-WT cells (Fig. 5H and fig. S25). We concluded that CD74 is an important mediator of IRF8 dysfunction in B cell lymphomas.

### In vivo examination of IRF8-mutant B cell lymphomas

**Fig. 6.**
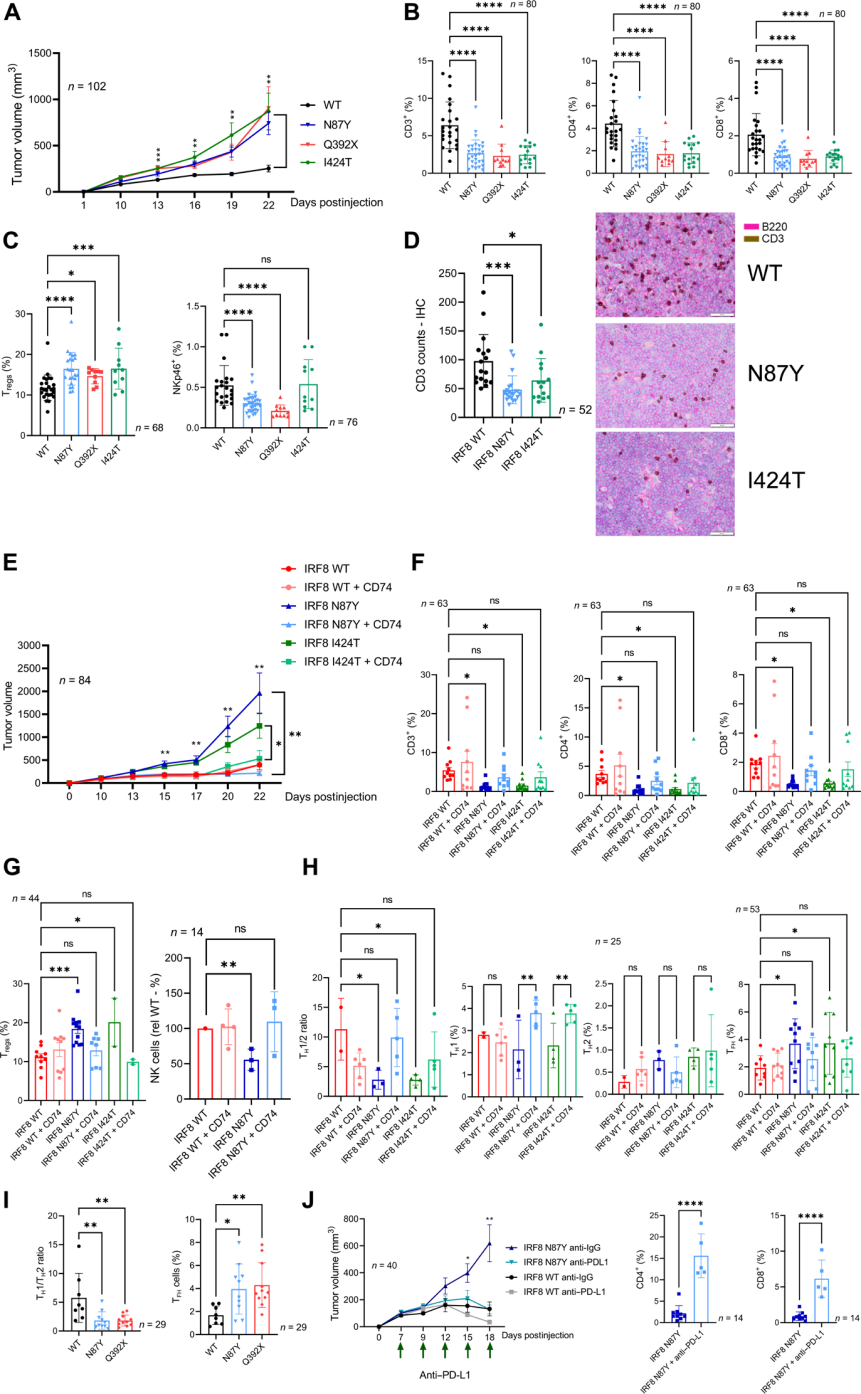
IRF8 effects on B cell lymphoma aggressiveness and immune microenvironment. (**A**) Growth curve of lymphomas expressing IRF8 WT, N87Y, Q392X, or I424T. (**B**) FACS-based quantification of CD3, CD4 and CD8 T cells in the TME of IRF8 WT or mutant lymphomas. (**C**) FACS-based quantification of T_regs_ and NK cells in the TME of IRF8 WT or mutant lymphomas. (**D**) IHC-based quantification of T cell infiltrate in B cell lymphomas expressing IRF8 WT, N87Y, or I424T. Representative staining (B220, pink; CD3, brown) is shown to the right, scale bar is displayed. (**E**) Growth curve of lymphomas expressing IRF8 WT, IRF8 N87Y or IRF8 I424T (−/+ CD74 expression). (**F**) FACS-based quantification of CD3, CD4 and CD8 in the TME of IRF8 WT or mutant lymphomas (−/+ CD74 expression). (**G**) FACS-based quantification of T_regs_ and NK cells in the TME of IRF8 WT or mutant lymphomas (−/+ CD74 expression). (**H**) T_H_1/T_H_2 ratio, T_H_1, T_H_2, and T_FH_ cells in the TME of IRF8 WT or mutant lymphomas (−/+ CD74 expression). (**I**) T_H_1/T_H_2 ratio and T_FH_ cells in the TME of IRF8 WT, missense (N87Y) or truncating (Q392X) mutant lymphomas. (**J**) Growth curve of lymphomas models expressing IRF8 WT or N87Y in mice treated with control antibody or anti–PD-L1 antibody; FACS-based quantification of CD4 and CD8 in IRF8 N87Y lymphomas treated with control or anti–PD-L1 antibody. For all panels, data are means ± SD of multiple independent cohorts (*n* indicated in the figure). *P* values are from one-way ANOVA with Fisher’s LSD posttest, Mann-Whitney test, or two-sided Student’s *t* test; *P ≤ 0.05, **P ≤ 0.01, ***P ≤ 0.001, ****P ≤ 0.0001.

Our in vitro data suggested that expression of mutant IRF8 in B cell lymphomas deregulates antigen processing/loading and ultimately presentation in an MHCII context thus eliciting a subpar CD4 response. Here, we investigated whether these in vitro findings translated into an IRF8 status–driven remodeling of the TME and whether it influenced tumor growth in vivo. To that end, we examined cohorts of syngeneic B cell lymphoma developed with A20 cells expressing IRF8 WT, N87Y, Q392X, or I424T (thus covering the two functional domains/clusters of IRF8 mutation in DLBCL, as well as missense and truncating mutations) grown in syngeneic BalbC mice (attempts to engraft 2PK-3 cells in BalbC mice were unsuccessful). In all assays, each mouse was injected subcutaneously with 5 million A20 cells, tumor volume was quantified at defined intervals and immune landscape examined by FACS immediately following mice euthanasia. In the TME, we initially quantified the total T cell infiltrate (CD3), the CD4^+^ and CD8^+^ subpopulations, regulatory T cells (T_regs_) (CD4^+^/CD127^−^, CD25^+^/intracellular Foxp3^+^), NK cells (NKp46^+^, i.e., CD335^+^), monocyte/macrophages (CD11b^+^), and the myeloid-derived suppressor cells (MDSCs) (CD11b^+^, Gr1^+^). Mice harboring lymphomas expressing IRF8 N87Y, Q392X, and I424T displayed significantly increased tumor growth (Fig. 6A). Consistently, a significant depletion of T cells (CD4^+^ and CD8^+^) was detected in the TME of lymphomas driven by an IRF8-mutant allele, irrespective of the variant analyzed (Fig. 6B and figs. S26 and S27). In addition, T_regs_ were significantly enriched in IRF8 N87Y, Q392X, and I424T lymphomas and NK cells depleted in N87Y and Q392X (Fig. 6C), while the monocyte/macrophage and MDSC infiltrates were quantitatively indistinguishable between IRF8 WT and mutant lymphomas (fig. S26). The T cell depletion in the TME of IRF8-mutant lymphomas was confirmed using IHC (Fig. 6D). The impact of IRF8 status on the in vitro growth pattern of these A20 models was unremarkable, with no significant difference between IRF8 WT, N87Y-, Q392X-, or I424T-expressing cells (fig. S28). Together, these data confirm that the role of IRF8 mutations is recognized principally in vivo, furthering the concept that immune escape, rather than a lymphoma cell intrinsic effect, is at the core of IRF8 dysfunction in B cell lymphomas. The differences noted in respect to tumor burden and the TME infiltrate were not related to the levels of ectopic expression of IRF8 WT or mutants, which were tightly controlled (Fig. 4B). Moreover, in agreement with the suggestion that IRF8 may be an essential gene in lymphoma (*37*), which could also explain why IRF8 mutations are monoallelic, A20 IRF8 KO cells failed to fully engraft in the syngeneic BalbC mouse (fig. S29).

Next, we investigated whether the IRF8-mutant phenotypes could be corrected by ectopic expression of CD74 in vivo. Tumor growth was significantly suppressed in IRF8-mutant lymphomas coexpressing CD74, while no difference was detected between IRF8 WT and IRF8 WT + CD74 (Fig. 6E); once more, only negligible differences were detected in in vitro growth rates supporting the role of the microenvironment in the differences detected in vivo (fig. S30). This reduced tumor aggressiveness was accompanied, and possibly secondary to, a marked remodeling of the TME, which after CD74 ectopic expression was not significantly different between IRF8 WT and IRF8 mutant lymphomas. In brief, ectopic coexpression of CD74 increased CD3, CD4, CD8, and NK numbers, and decreased T_reg_ abundance in IRF8 N87Y and I424T lymphomas (Fig. 6, F and G, and fig. S31). In an attempt to define the entire spectrum of IRF8 mutant–associated changes in subpopulations of CD4 T cells, we expanded the investigation to T helper 1 (T_H_1), T helper 2 (T_H_2), T follicular helper (T_FH_), and T_H_17 cells. In IRF8 N87Y- and I424T-driven lymphomas, we detected a significantly smaller T_H_1/T_H_2 ratio, which was corrected to the IRF8 WT “baseline” by CD74 expression (Fig. 6H and fig. S32). Furthermore, we found a significant increase in T_FH_ cell infiltrates in IRF8-mutant lymphomas, which was also normalized by CD74 coexpression (Fig. 6H and fig. S33). The accumulation of T_FH_ cells and aberrant T_H_1/T_H_2 polarization was also readily detectable in the TME of IRF8 Q392X–expressing lymphomas (Fig. 6I and fig. S34), suggesting that missense and nonsense (truncating) mutations promote similar deregulation. In contrast, we found that the IRF8 status does not influence T_H_17 infiltration in the TME (fig. S27).

A recent report showed that IRF8 expression in tumor-associated macrophages (TAMs) promoted T cell exhaustion and that, in agreement with our findings in B cell lymphoma models, IRF8 was required for TAMs to properly present cancer cell antigens (*58*). T cell exhaustion is often a harbinger for diminished immune checkpoint therapy (*3*). Therefore, we decided to examine whether the IRF8 mutant–associated remodeling of the TME and attendant growth advantage that we detected in B cell lymphoma models, was still amenable to correction with checkpoint inhibitors of the PD-1/PD-L1 pathway. To test this possibility, we designed a treatment trial in which, following tumor engraftment, mice harboring IRF8-WT or IRF8-N87Y lymphomas were dosed with an anti–PD-L1 antibody or control antibody every 72 hours. Anti–PD-L1 treatment was effective in inhibiting the highly aggressive IRF8 N87Y mutant, with accompanying increase in the CD4/CD8 infiltrate (Fig. 6J). Expression of mutant IRF8 did not modify PD-L1 expression in this lymphoma model, which is negative for PD-1 (fig. S35).

We concluded that missense and truncating IRF8 mutation rewires the lymphoma microenvironment toward a broad procancer profile. This perturbation increases tumor aggressiveness of IRF8-mutant lymphomas, which is clinically and immunologically corrected by CD74 expression or anti–PD-L1 treatment.

### Impact of IRF8 mutation on the microenvironment of primary human DLBCL

**Fig. 7.**
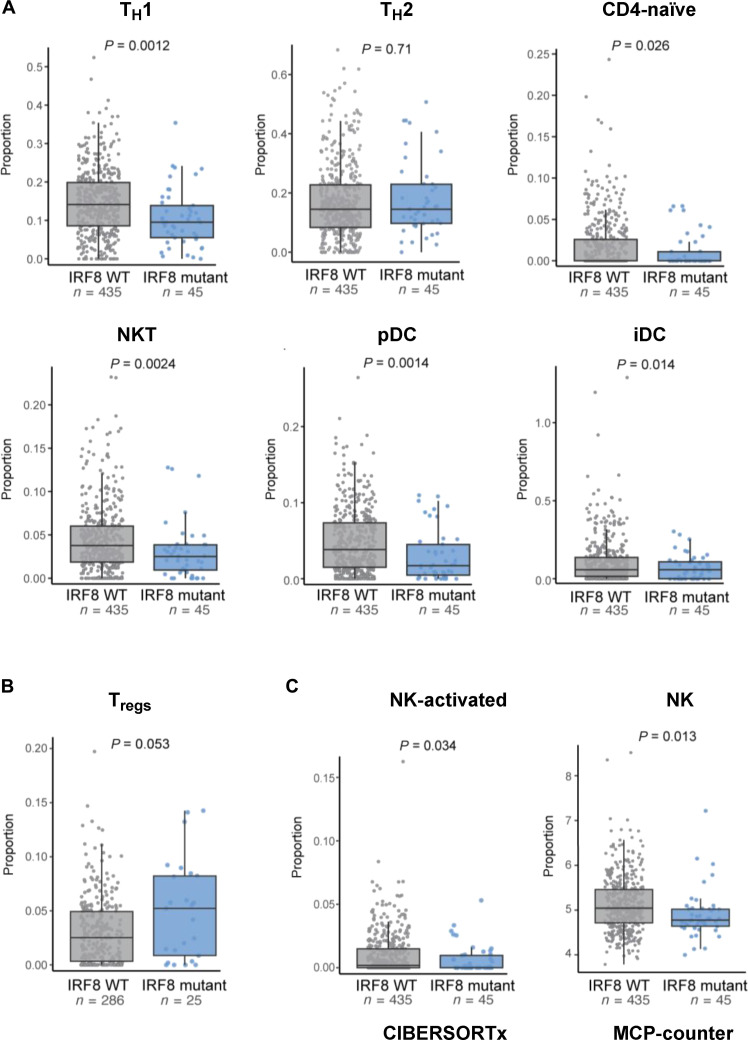
Immune composition of the microenvironment of IRF8-mutant human primary DLBCLs. (**A**) From left to right, top to bottom. xCELL-defined score estimation of T helper cell (T_H_1 and T_H_2), naïve CD4, NKT, pDCs, and immature DCs (iDCs) in IRF8 WT (*n* = 435, gray box plot) or IRF8-mutant (*n* = 45, blue boxplot) DLBCLs. (**B**) Estimated T_reg_ proportions in IRF8 WT (*n* = 286 gray box plot) or IRF8 mutant (*n* = 25, blue box plot) measured using CIBERSORTx. (**C**) Left to right: Estimated proportions of NK activated (CIBERSORTx) or estimated scores of total NK cells (MCP-counter) in IRF8 WT (*n* = 435, gray boxplots) or IRF8-mutant (*n* = 45, blue boxplots) DLBCLs. In all panels, the boxplots show median and interquartile range. *P* values are from two-sided Student’s *t* test.

To test whether the IRF8 mutant–driven remodeling of the lymphoma microenvironment found in mouse models of B cell lymphoma could be recapitulated in primary human DLBCL, we used deconvolution of bulk RNA sequencing (RNA-seq) data (dbGaP Study Accession: phs001444.v2.p1). This cohort of 480 DLBCLs was first reanalyzed for IRF8 mutation calling, and a total of 45 lymphomas were found to harbor an IRF8 variant allele (table S7). Next, we used the xCell algorithm (*59*) to identify putative distinctions in the nonmalignant cell component of IRF8 WT and mutant DLBCLs. In agreement with the mouse B cell lymphoma models, IRF8-mutant DLBCLs displayed significantly diminished signatures of NK cell infiltrate as well as selected CD4 subpopulations, including T_H_1, but not T_H_2 (Fig. 7A). In addition, the TME of IRF8-mutant DLBCLs was also significantly depleted of plasmacytoid DCs (pDCs), and of the related interstitial DCs (iDCs). NKT, T_H_1, and pDC all secrete proinflammatory cytokines, chiefly among them IFN-γ (*60*–*62*). Still, using xCell, we did not detect significant changes in T_regs_ in IRF8-mutant primary DLBCL (fig. S36), which were consistently detected in the A20 BalbC models (T_FH_ cells are not uniquely identified with xCell). This result was somewhat unexpected because recently, IRF8 mutation in DLBCL was identified as a putative marker for poor response to CD19-CART cells (*63*). Moreover, in that study, it was postulated that this worse outcome could be related to an increase in T_regs_ in the microenvironment of IRF8-mutant DLBCL, which therein was detected by deconvolution of bulk RNA-seq signatures using another algorithm, CIBERSORTx (*64*). Thus, we reanalyzed the primary DLBCL data with CIBERSORTx, and in a cohort that excluded tumors with mutations in other genes known to deregulate antigen presentation (CIITA, B2M, CREBBP, EP300, and EZH2), we detected an increase in T_regs_ (*P* = 0.05) in IRF8-mutant DLBCLs (Fig. 7B and fig. S37). These data also mitigate concerns that, because of the high frequency of IRF8 mutations in the EZB subgroup (Fig. 1C), part of the IRF8 signature may derive from co-occurring EZH2 mutation. Using CIBERSORTx, and also adding a third algorithm, MCP-counter (*65*), we confirmed depletion of NK cells as the most reproducible change in the TME of IRF8-mutant human DLBCL (Fig. 7C and fig. S38). Notably, T_H_1 cells and pDC are not uniquely identified subpopulations in deconvolution performed with CIBERSORTx or MCP-counter; thus, the xCELL data on those subsets could not be cross validated. We concluded that in the highly heterogenous setting of primary DLBCL, the presence of an IRF8 mutation associates with a TME signature characterized by depletion of a series of proinflammatory/anticancer cell types (of the innate and adaptive immune system) and a potential increase in the suppressive T_regs_. This profile partially recapitulates the microenvironment remodeling detected in the mouse model of IRF8 WT or mutant B cell lymphoma that we developed.

## DISCUSSION

Here, we showed that B cell lymphomas carrying *IRF8* gene mutations, missense or truncating, are better equipped to evade the immune system than those with IRF8 in a WT configuration. Mechanistically, this escape is mediated, at least in part, by a defective expression of IRF8 targets that tightly control antigen loading into the MHCII complex, including the CD74/invariant chain (Ii) and HLA-DM, as well as CIITA, the master regulator of MHCII associated antigen presentation (*34*) and, in a minority of models, cell surface HLA-DR. We confirmed that IRF8 binds to the promoters of these genes and showed that IRF8 mutation impairs its binding to the regulatory regions of *Cd74*, *H2-Dm*, and *Ciita*.

In our model system, ectopic expression of CD74 was sufficient to rescue the immune and clinical phenotypes detected in IRF8-mutant B cell lymphomas, but it did not significantly change the immune landscape or growth pattern of IRF8 WT B cell lymphomas. These data place IRF8 at a control hub for antigen loading and reinforces the concept that these processes must be executed with precision, lest antigen presentation is defective, and, in a cancer context, immune evasion is elicited. Fortuitously, we detect a significant impact of IRF8 KO or mutation in an assay (CD4 DO-11.10 cells activation) in which the antigen loaded into the lymphoma cells was already processed (the OVA peptide). These findings, together with defective expression of CD74 and HLA-DM, and the CD74-mediated rescue of IRF8-mutant lymphomas, suggest that of the many steps involved in the antigen processing/presentation cascade, IRF8 may impinge primarily on loading, and possibly not on acquisition, tagging, proteolysis, trafficking, and display (*34*).

The mutation pattern of *IRF8* in DLBCL (and related B cell malignancies) is similar to that of *CREBBP*, *EP300*, *KMT2D*, and *TET2*, among other genes (*12*, *13*, *36*, *37*). The variants are predominantly missense or truncating, often hemizygous, clustered in important domains of the protein, and functionally hypomorphic. A model of haploinsufficiency can be ascribed to this pattern. However, we suggest that IRF8 may be unique in its lymphomagenic potential because it has also been associated with a variety of oncogenic structural defects [translocation, amplification, and super-enhancer deregulation (*15*–*17*)] in which excess of IRF8 expression may contribute to lymphoma development and progression. In agreement with this postulate, in an unbiased CRISPR screen in DLBCL cell lines, IRF8 was identified as an “essential oncogene” (*37*), and we found the IRF8 deletion abolishes B cell lymphoma growth in vivo. Together, we propose that both gain or loss of IRF8 function play a role in B lymphoma biology, possibly by influencing cell intrinsic processes and immune evasion, respectively.

In mice, the IRF8-mutant driven remodeling of the lymphoma microenvironment was diverse but consistently reflected a procancer profile (decrease in CD4, CD8, NK, and T_FH_1 and increase in T_regs_ and T_FH_ cells) (*62*). The initiating event, we postulate, was the defective antigen loading (and its eventual display on the cell surface) because ectopic expression of CD74 restored the entire repertoire of changes to an “IRF8 WT baseline.” Of the immune perturbations that we detected in the in vivo model of IRF8-mutant B cell lymphoma, previous associations existed with NK cells depletion and T_reg_ accumulation. In respect to the former, IRF8 mutations (some of which are also present in DLBCL) were found in familial cases of human NK deficiency (*23*). In those instances, biallelic germline IRF8 variants were associated with decreased NK numbers, as well as cell defective NK cell maturation and function. In addition, in a mouse of conditional Irf8 deletion in NK cells, IRF8 was found to be essential for NK cell proliferation, expansion, and adaptive cell responses (*66*). However, in these two instances, IRF8 is either mutated in the germ line or specifically deleted in developing NK cells, thus not fully recapitulating our model (or primary DLBCLs), wherein only the B cell lymphoma cell is IRF8 mutant. Loss of IRF8 can also impair NK function in a cell-extrinsic manner, including defective cytokine secretion by IRF8 null DC (*67*, *68*). Thus, in the context of IRF8-mutant B cell lymphoma, it is possible that still to be identified secreted factors may limit NK recruitment to the tumor milieu. A similar process may be operational toward the pDC, which although not measured in the in vivo mouse model, were significantly depleted in the IRF8-mutant primary DLBCLs. We propose a circuitry wherein the poor antigen presentation by IRF8-mutant lymphomas (due to deregulated CD74/antigen loading) blunts the activation and differentiation of naïve CD4^+^ T cells toward the T_H_1 phenotype, decreasing the secretion of proinflammatory cytokines such as IFN-γ, which in turn limits the recruitment of NK, NKT, and pDC cells, all themselves significant sources of IFN-γ and tumor necrosis factor α (*60*, *61*), thus further suppressing the beneficial inflammatory profile of the TME.

The potential link between IRF8 and T_regs_ emerged more recently, when IRF8 mutations in relapsed DLBCL was identified as a putative marker for poor response to CD19-CAR T cells, possibly in association with an increase in T_regs_ in the lymphoma microenvironment (*63*). Our in vivo model of IRF8-mutant B cell lymphoma validated this initial observation and linked it to defective antigen loading into MHCII complexes because it could be rescued with CD74 ectopic expression. Further, using the same deconvolution algorithm used in the CAR-T study, we detected an increase in T_regs_ in a subset of IRF8-mutant DLBCLs. Last, we also detected a significant increase in T_FH_ cells in the mouse models of IRF8-mutant B cell lymphoma, which was corrected by CD74 ectopic expression. This finding made immediate sense given the role of these cells in supporting B cell expansion in germinal centers (*69*), their role in the lymphomagenesis associated with Cathepsin S– and *TNFRSF14*-mutant lymphomas (*70*, *71*), and their positive association with poor prognosis in B cell lymphomas (*72*). However, this finding was not validated in primary DLBCL tumors, although this examination was limited by the fact that only one of the three algorithms that we used to deconvolute the bulk RNA-seq data identifies T_FH_ cells as a discrete subpopulation. Further studies in primary DLBCLs are needed to confirm or refute our mouse model data.

By computational modeling of IRF8 structure and building on the solved structure of the related IRF3 protein in complex with DNA, we gained insight into the mechanisms by which IRF8 mutations may deregulate protein architecture and function. Unexpectedly, these preliminary observations implied that in addition to the well-defined N-terminal DBD, the C-terminal tail of IRF8 may also contact the DBD/DNA. This unexpected finding may provide clues to the similarity in phenotype that we detected between DBD and C-terminal tail mutants (N87Y and I424T, respectively), and it could help explain the high frequency of truncating mutations clustered at the C terminus of IRF8.

Our study has limitations. The impact of IRF8 mutation on HLA-DM expression appears to be stronger in human DLBCL than in murine B cell lymphomas whereas CD74 defect is clearer in the latter. The selection to examine further CD74, instead of HLA-DM, was dictated by the model system but it is possible that in a human system, ectopic expression of HLA-DM may also rescue the IRF8-mutant phenotype. In addition, it remains to be elucidated why the impact of IRF8 on CIITA activity does not consistently translate into a decrease in MHCII on the cell surface. The fact that IRF8 mutants do not consistently lose binding to HLA-DR promoter may be part of the explanation. At the moment, we cannot exclude the possibility that expression of partner proteins, cytokine signaling/B cell activation status, and/or chromatin accessibility may also be implicated in this phenomenon. In addition, although CIITA has a dominant role in the control of MHCII expression on the cell surface, the impact of IRF8 on CIITA expression may not be sufficient to modify HLA-DR. Also, the IRF8-mutant models that we examined in vitro and in vivo are monoallelic, and it is possible that the presence of the WT allele may uncover further aspects of the IRF8 role in B cell lymphomas. Nonetheless, all comparisons were made to a similarly generated IRF8 WT model, mitigating concerns that IRF8 dosage, instead of the nature of the IRF8 isoform expressed, accounted for the results. In summary, with this work, we added IRF8 to the list of genes that when mutated contribute to lymphomagenesis at least in part by facilitating escape from immunosurveillance.

## MATERIALS AND METHODS

### Cell lines

The DLBCL cell lines SU-DHL4 (male), SU-DHL6 (male), Toledo (female), SU-DHL2 (female), RIVA (also known as RI-1, female), the mouse B cell lymphoma cell line A20 (Balb/c), 2PK-3 (Balb/c), the B cell leukemia/lymphoma cell line BCL1 (Balb/c), and the mouse macrophage cell line RAW 264.7 were cultured at 37°C in 5% CO_2_ in RPMI 1640 medium (Invitrogen) containing 10% (v/v) fetal bovine serum (FBS). The human embryonic kidney (HEK) 293 T cells (Thermo Fisher Scientific) were maintained in Dulbecco’s modified Eagle’s medium (Mediatech) with 10% FBS, as we described (*73*). The identity of the cell lines was confirmed by variable number of tandem repeats analysis and verified online at the Deutsche Sammlung von Mikroorganismen und Zellkulturen (DSMZ) and American Type Culture Collection (ATCC) cell banks and tested for Mycoplasma. All cell lines were preexistent in the investigator’s laboratory and/or were earlier obtained from ATCC, DSMZ or, Thermo Fisher Scientific.

### Establishment of genetic models

#### Human DLBCL cell lines

IRF8 was knocked out in SU-DHL4, SU-DHL6, SU-DHL2, and RIVA IRF8 using CRISPR-Cas9 technology. In brief, two distinct guide RNAs (table S8) were independently cloned into lentiCRISPR v2 plasmid, which was cotransfected with the PAX2 and pMD2.G plasmids into HEK-293 T cells to generate lentivirus, as we described (*74*). Subsequently, the cell lines were transduced twice by spinoculation, selected with puromycin selection and clones generated by limiting dilution (SU-DHL4 and SU-DHL6) or polyclonal populations with complete KO (SU-DHL2 and RIVA) examined in downstream assays, as we reported (*75*). The DLBCL cell line Toledo, which does not express endogenous IRF8, was transduced with empty murine stem cell virus (MSCV)–enhanced green fluorescent protein (eGFP) or IRF8 WT and mutants S55A, N87Y, D400G, and I424T, all in a MSCV-eGFP backbone. In brief, IRF8 WT or mutant was generated by reverse transcription polymerase chain reaction (RT-PCR) and site-directed mutagenesis, as we described (*76*), and Sanger sequence validated. These constructs were cotransfected with vesicular stomatitis virus glycoprotein and pKAT into HEK-293 T to produce retrovirus. The retrovirus was transduced twice into Toledo cells by spinoculation, followed by GFP sorting using FACS.

#### Mouse cell lines

IRF8 KOs were generated in the macrophage cell line RAW 264.7, in the B cell lymphoma cell lines A20 and 2PK-3 and in the B cell leukemia/lymphoma cell line BCL1 (all from ATCC), as described above for human cell lines, except for the use of four independent guide RNAs (table S8). In each cell line, at least two KO clones (obtained by limiting dilution) derived from different guide RNAs were used in downstream assays. In addition, A20 and 2PK-3 IRF8 KO clones were transduced with human IRF8 WT or mutants (S55A, N87Y, Q392X, D400G, and I424T) cloned into a MSCV-eGFP backbone, followed by FACS to purity (>95% GFP^+^ cells), as described above for human cell lines. CRISPR-Cas9 technology, with guide RNA cloned into lentiCRISPR v2 plasmid, was also used to KO cd74 in A20 and 2PK-3 cell lines. Last, murine CD74 (p41 isoform) cloned into a lentivirus backbone with an MSCV promoter (pLV[Exp]-Neo-MSCV>FLAG/mCd74, Vector Builder Inc.) was transduced in A20, BCL1, and 2PK-3 Irf8 KO cells, as well as A20 irf8 KO cells stably expressing IRF8 WT, IRF8 N87Y, and IRF8 I424T. All KO and ectopic expression models were confirmed by sequencing and/or Western blotting.

### Mice and lymphoma development in vivo studies

Six-week-old male and female BALB/cJ mice were purchased from the Jackson Laboratory (strain no.: 000651). Each mouse was injected subcutaneously with 5 million A20 cells murine B lymphoma cell line A20, as we described (*77*). Six independent cohorts were generated (#1 = 25 mice, #2 = 25 mice, #3 = 40 mice, #4 = 20 mice, #5 = 60 mice, #6 = 30 mice). The A20 cell models used were generated by a CRISPR-Cas9–mediated KO of mouse Irf8 (lentiCRISPR-V2 puromycin lentivirus), and subsequent stable ectopic expression of empty MSCV-eGFP, IRF8 WT, N87Y, Q392X, or I424T, alone or together with murine CD74 (p41 isoform) cloned into a lentivirus backbone with an MSCV promoter (pLV[Exp]-Neo-MSCV>FLAG/mCd74, purchased from Vector Builder Inc.). The mice were monitored daily and starting 10 days after cell injection, the subcutaneous tumors were measured every 72 hours using an electronic caliper, as we described (*78*). In one cohort, mice harboring lymphomas expressing IRF8 WT or N87Y were randomized to receive five doses of anti-mouse PD-L1 (clone 10 F.9G2, catalog no. BE0101, Invivomab) or rat IgG2b isotype control (catalog no. BE0090, Invivomab), administered intraperitoneally (200 μg per injection) at 2- to 3-day intervals. Following mice euthanasia, all the collected tumors were weighed, measured, and photographed; and single cells were immediately generated for downstream FACS analysis. A local colony of TCR (Vα2 and Vβ5) transgenic mice (OT-I) (strain no.: 003831, Jackson Laboratories) was maintained by breeding homozygous transgenic mice. Male and female mice, 8 to 12 weeks old, were euthanized, spleens harvested, and T lymphocytes isolated, and CD8 cells differentiated in vitro for antigen presentation assays (see below). All the animal procedures were approved by the Animal Care and Use Committee of the UT Health San Antonio.

### Data retrieval and somatic variants selection

The data used in this study were obtained from the Genomic Variation in Diffuse Large B Cell Lymphomas project (project ID NCICCR-DLBCL) available on the genome data commons (GDC) data portal (dbGaP Study Accession: phs001444.v2.p1). The .bam files obtained from the GDC data portal had already been aligned to the GRCh38.p0 reference genome using BWA, the Mark Duplicates, and base quality score recalibration workflows. The variants in the aligned files were called and selected as previously reported (*13*) with the modifications listed below. In brief, we defined the genomic regions of interest, used the VarScan2 software (*79*), and selected the parameters of a read count greater than or equal to 3, and a variant read frequency greater than or equal to 0.1 to select relevant variants. To identify variants that had the highest impact on gene function, we implemented a series of filtering steps. We applied specific exclusion criteria to filter out variants with a population frequency above 0.0001 in the Genome Aggregation Database (gnomAD). We restricted our analysis to missense or nonsense point mutations, truncating mutations, and exonic indels. In addition, we considered the variant selections made previously (*13*) to finalize our list of somatic variants for downstream analysis. Variant allele frequency was obtained from VarScan2 or cBioportal cohorts listed in table S1. Classification in COO groups or subgroups were assigned from the original reports (table S1) or by the LymphGen data portal (https://llmpp.nih.gov/lymphgen/lymphgendataportal.php?version=2.0#).

### Mutual exclusivity analysis

We performed a mutual exclusivity analysis using the Oncoprint function available in cBioPortal (*80*). We first selected a set of genes of interest based on their known relevance in DLBCL. We then used the Oncoprint function to visualize the mutual exclusivity patterns of these genes in our selected dataset. We defined mutual exclusivity as the absence of co-occurrence of mutations between two or more genes within a sample. We calculated the statistical significance of the mutual exclusivity using the Fisher’s exact test and corrected for multiple testing using the Benjamini-Hochberg procedure. We considered a stringent *q* value (false discovery rate) of <0.05 as statistically significant.

### ChIP-seq data analysis

We retrieved publicly available CIITA and IRF8 ChIP-seq data in human and mouse cell lines from Gene Expression Omnibus website, including GSE52941 for human Raji cell line, and GSE128166/GSE104921 for mouse B lymphocytes. After adaptor sequence was removed, all reads were aligned to human genome (hg19) or mouse genome (mm9) using bowtie with “–best –strata –m 1” parameters. Duplicated reads were eliminated for subsequent analysis. Model-based Analysis of ChIP-seq 2 was used to call peaks comparing immunoprecipitated chromatin with input chromatin using standard parameters and a *q* value cutoff of 1 × 10^−5^. Peaks that overlapped with the blacklist regions from UCSC were removed. All ChIP-seq data were visualized in Integrative Genomics Viewer. The reads were extended to 200 nt in the 5′-to-3′ direction and normalized to 10 million uniquely mapped reads per experiment.

### Reporter assay

The CIITA reporter constructs (−709 to +100 of CIITA promoter region), cloned into pgL3 in WT configuration or with point mutations in the EICE site, were a gift from K. Wright, Moffit Cancer Center, and had been characterized earlier (*51*). Their identity was confirmed by Sanger sequencing (EICE WT site GGTTTTCACTTC, mutant GCAGTTCACTTC). The CIITA WT or EICE mutant promoter vectors were independently cotransfected with pCMV β-gal plasmid into RAW 264.7 cells seeded at 5 × 10^5^ in 12-well plates. The RAW 264.7 cells were first subjected to CRISPR-Cas9–mediated KO of Irf8 (CRISPR-V2 puromycin lentivirus) and subsequently transduced to stably express human IRF8 WT or mutant (S55A, N87Y, D400G, and I424T) in the MSCV-eGFP retrovirus, as we reported (*81*). Twenty-four hours after transfection, the RAW 264.7 cells were washed with ice-cold phosphate-buffered saline (PBS) and harvested in 250 μl of reporter lysis buffer (Promega luciferase assay system) with gentle shaking for 15 min at room temperature. The lysate mixture was spun at 14,000 rpm for 30 s, and the supernatant was collected for the luciferase assay. Briefly, 20 μl of lysate was added to a 1.5-ml Eppendorf tubes containing 100 μl of luciferase assay reagent II (Promega luciferase assay system, catalog no. E1500), and the mixture was pipetted in and out three times. The luminescence reading was performed in Modulus single tube multimode reader (Turner Biosystems). The β-galactosidase activity was measured in 30 μl of lysate incubated with 20 μl of reporter lysis buffer and 50 μl of 2X β-gal Enzyme Assay buffer (Promega, catalog no. E2000). The absorbance was read in iMark microplate reader (BioRad), in 96-well plates, following incubation at 37°C for 2 hours and addition of 150 μl of stop solution (1 M sodium carbonate).

### Antigen presenting assay (DO-11-10 CD4 T cells)

Mouse B cell lymphoma B cell lines, A20, 2PK-3, and BCL1 were plated at 5 × 10^4^ cells per well per 100 μl of culture media in 96-well plates, followed by “antigen loading” with the addition of the OVA peptide (OVA-323-339) (catalog no. 12787-01, Bio-Synthesis) for 3 hours. A20 and BCL1 cells were “loaded” with 5 μg/ml, whereas 2PK-3 cells were loaded with OVA (10 μg/ml). Cells were then washed with growth medium and cocultured with DO-11.10 CD4 T cells, as reported (*54*); 8 × 10^4^ of DO-11.10 cells in 200 μl of culture media were added to the A20 and BCL1 cells, whereas 1.3 × 10^5^ of DO-11.10 cells were added to 2PK-3 cells per well. The plates were centrifuged at 500*g* for 5 min to initiate contact between the cells and placed at 37°C in a CO_2_ incubator for multiple time points. In brief, after 6 hours in coculture, supernatants from the A20 and DO-11.10 cells were collected for IL-2 detection by enzyme-linked immunosorbent assays (ELISAs) using commercially available sandwich-based kit (R&D Systems, catalog no. M2000), according to the manufacturer’s instructions. After 24 hours in cococulture, these cells were harvested and analyzed for surface expression of CD4 and the T cell activation markers CD69 and CD25 by flow cytometry as described below. Supernatants from BCL1 and 2PK-3 cells in coculture with DO-11.10 were collected after 24 hours of incubation and analyzed for IL-2 detection by ELISA. At the same time, cells were harvested for surface expression of CD4, CD69, and/or CD25.

### Isolation/activation of CD8 T cells from OT-I mice and in vitro cytotoxicity assay

Spleens were collected from 8- to 12-week-old TCR (Vα2, Vβ5) transgenic mice (OT-I) (strain no.: 003831, Jackson Laboratories) and T lymphocytes purified using Easy Sep Mouse T cell isolation kit (STEMCELL Technologies, catalog no. 19851A), as we described (*82*). To generate CD8^+^ T lymphoblasts responsive to OVA peptide (OVAp; SIINFEKL OVA257–264, catalog no. S7951, Sigma-Aldrich), T cells were incubated for 48 hours with OVAp (10 ng/ml), and mouse recombinant IL-2 (50 U/ml) (catalog no. 575402, Biolegend) was added for a period of five additional days, as described (*83*), at which time FACS analysis confirmed that 99.9% of the cells were CD8^+^. Subsequently, these activated CD8^+^ T lymphoblasts were cocultured with IRF8 WT or KO mouse B cell lymphoma cell lines (A20, 2PK-3, and BCL1). In brief, lymphoma cell lines were pulsed with 1 μM OVAp for 2 hours, washed twice with PBS, and cocultured with mice CD8^+^ T cells at an effector-to-target ratio of 5:1 for 6 hours at 37°C, after which the cytotoxicity toward IRF8 WT or KO lymphoma cells was assessed by FACS using 7-aminoactinomycin D (7-AAD) (BD Biosciences, catalog no. 51-68981). Controls of non-OVAp pulsed B cell lymphomas were included in all instances. All assays were subjected to three biological replicates.

### Granzyme B detection assay

Conditioned media from coculture of OT-I activated CD8^+^ T cells with mouse B cell lymphoma (OVAp pulsed and nonpulsed controls) were assessed for the concentrations of granzyme B by ELISA using mouse granzyme B specific kit (RayBiotech, catalog no. ELM-GranzymeB-1), according to the manufacturer’s instructions. In brief, after 6 hours of coculture, cell supernatants were diluted 1/100 in an assay diluent buffer provided and loaded on an ELISA plate precoated with mouse granzyme B antibody for 2.5 hours at room temperature. Plates were washed followed by 1.5-hour incubation with secondary biotinylated anti-mouse granzyme B antibody. Horseradish peroxidase (HRP)–conjugated streptavidin was then added to the plates for an hour at room temperature. A colorimetric signal was developed on the enzymatic reaction of HRP with a chromogenic substrate, 3,3′,5,5′-tetramethylbenzidine for 30 min. After stopping the reaction with 0.2 M sulfuric acid, the values were determined by measuring the optical density of ELISA plate wells at 450 nm. Concentrations were calculated in picograms per milliliter on the basis of the standard curve generated. All assays were subjected to three biological replicates.

### Flow cytometry - cell surface or intracellular staining

#### Cell line models

One million viable human DLBCL and mouse B lymphoma cell lines were counted and resuspended in 100 μl of FACS buffer (2% FBS and 1 mM EDTA in PBS). Cells were stained with the respective fluorochrome-conjugated antibodies for detection of HLA-DR, H2-IA/IE, CLIP, PD-1, and PD-L1 surface proteins and incubated for 30 min at 4°C. Cell were then washed with FACS buffer and resuspended in FACS buffer containing 4′,6-diamidino-2-phenylindole (DAPI) (5 mg/ml, diluted 1:5000) for live/dead cell exclusion. For intracellular detection of HLA-DM, H2-DM, or Li invariant chain/CD74, cells were fixed and permeabilization using the Intracellular Fixation & Permeabilization Buffer kit (eBioscience, catalog no. 88-8824-00) and stained with the corresponding antibodies.

#### In vivo lymphoma microenvironment

To quantify and characterize the immune infiltrates in the syngeneic lymphoma engraftment models, tumors were harvestedt, and single-cell suspensions were generated, as reported (*84*). Two million viable cells were resuspended in 100 μl of FACS buffer with 1 μl of Fc Block (anti-mouse Fc block CD16/32, catalog no. 101302, Biolegend) for 20 min at 4°C. Cells were then washed with FACS buffer and stained with surface markers antibodies to detect the following populations: (i) T cell subpopulations (CD3, CD4, and CD8 surface markers), (ii) monocyte/macrophage subpopulations (CD11b surface marker), (iii) NK cell population (Nkp46 surface marker), (iv) MDSC population (double-positive CD11b, Gr1 surface markers), (v) T_H_1 cell population (CD4-, CXCR3-, and CCR5-positive surface markers), (vi) T_H_2 cell population (CD4-, CCR3-, and ST2-positive surface markers), and (vii) T_FH_ cell population (CD4-, CXCR5-, and PD-1–positive surface markers). Cells were washed with FACS buffer and resuspended in FACS buffer containing a viability dye 7-AAD (catalog no. 51-6898, BD Bioscience) or DAPI (catalog no. 62248, Thermo Fisher Scientific) (1:5.000 dilution), according to the fluorophores used. Fluorescence compensation was performed using UltraComp eBeads (catalog no. 01-2222-42, Invitrogen). For ex vivo detection of T_regs_, 6 million viable single cells harvested from mouse lymphomas were surface stained with BV421 anti-mouse CD4, antigen-presenting cell anti-mouse CD127 (IL-7Rα), and BV650 anti-mouse CD25, followed by fixing/permeabilization using the True-Nuclear transcription factor buffer set (Biolegend) according to the manufacturer’s instructions. Cells were next stained with phycoerythrin anti-mouse FoxP3 antibody. CD4^+^/CD127^−^ cells were analyzed for CD25 and intracellular FoxP3. A preincubation with Fc blocking antibody was performed. Instrument compensation was performed with UltraComp eBeads. T_H_1, T_H_2, and T_FH_ cell populations were also detected with a combination of cell surface markers and intracellular transcription factor staining (T_H_1 = Tbx21; T_H_2 = GATA3; T_FH_ = BCL6). T_H_17 cells were detected with CD4, ccr6 (cell surface), and RORg (intracellular). In brief, cells were stained with surface markers, washed, and resuspended in PBS with fluorescent reactive fixable viability dye (LIVE/DEAD Fixable Blue Dead Cell Stain Kit, for ultraviolet excitation, Invitrogen, catalog no. L34961). This was followed by fixing/permeabilization using the True-Nuclear transcription factor buffer set and stain with the corresponding transcription factor antibody. T_H_1 cells were analyzed for CD4/CXCR3/Tbx21, T_H_2 for CD4/ST2/GATA3, T_FH_ for CD4/CXCR5/BCL6, and T_H_17 for CD4/CCR6/RORgt.

Antibodies used for flow cytometry were the following: anti-human HLA-DR and anti-human HLA-DM, (catalog no. 361610 and catalog no. 358004, Biolegend), anti-human CD74 (catalog no. FAB35901N, R&D Systems); anti-mouse H2I-A/I-E, (catalog no. 10763), anti-mouse CD3 (catalog no. 100206), anti-mouse CD8a (catalog no. 100712), anti-mouse CD11b (catalog no. 101208), anti-mouse Nkp46 (catalog no. 137612), anti-mouse CD195 (CCR5) (catalog no. 107011), anti-mouse CD279 (PD-1) (catalog no. 135224), anti-mouse CD185 (CXCR5) (catalog no. 145506), anti-mouse IL-33Rα (ST2) (catalog no. 146607), anti-mouse CD193 (CCR3) (catalog no. 144527), anti-mouse CD4 (for T_reg_ quantification, catalog no. 116023), anti-mouse FoxP3 (catalog no. 126403), anti-mouse CD25 (catalog no. 102037), anti-mouse CD127 (IL-7Rα) (catalog no. 135011), anti-mouse Fc block CD16/32 (catalog no. 101302), all from Biolegend; anti-mouse H2-DM (catalog no. 624048), anti-mouse CD4 (catalog no. 563232), anti-mouse CD25 (catalog no. 553866), anti-mouse CD183 (CXCR3) (BDB755832), all from BD Biosciences; anti-mouse CD74 (FAB7478A, R&D Systems), anti-mouse Ly-6G/Gr1 (catalog no. 17-9668-82, eBioscience). anti-mouse RORgt (catalog no. BDB562607, (BD Bioscience), anti-mouse GATA3 (catalog no. 50604769), anti–T-BET (catalog no. 50170980), anti-Human/mouse BCL6 (catalog no. 50604576), anti-mouse CD196 (catalog no. NC1898560); anti-mouse PD-1 (catalog no. 135224), anti-mouse PD-L1 (catalog no. 17-5982-82), anti-mouse B220 (catalog no. 103208), anti-mouse CD69 (catalog no. 104514), all from Biolegend.

All flow cytometry analyses, were performed on a BD LSR II (Becton Dickinson), equipped with a BD FACSDiva software v8.0.1. (BD Bioscience), except for T_H_1 cells which were analyzed in a BD FACSCelesta instrument. All subsequent compensation and gating were performed with FlowJo analysis software (BD LifeSciences). Gating strategies were set using the appropriate isotype control for each fluorochrome-conjugated staining antibody used. All isotype control antibodies were from BD Biosciences and used at 1:500 dilution.

### Protein isolation and Western blotting

Mouse and human whole cell lysates were extracted with a buffer containing 1% NP40, 50 mM tris (pH 8.0), 150 mM NaCl, 10% glycerol, 2 mM EDTA, and 10× concentration of Halt Protease and Phosphate Inhibitor Cocktail (Thermo Fisher Scientific) for 30 min on ice and spun at 13,000 rpm for 15 min at 4°C. Protein samples were quantified using Protein Assay Dye Reagent (Bio-Rad). Before electrophoresis, samples were denatured in 5× laemmli buffer (8% SDS, 0.4 M DTT, 0.2 M tris-HCl, 4.3 M glycerol, and 6 mM bromophenol blue) and separated by SDS–polyacrylamide gel electrophoresis (SDS-PAGE), transferred to immobilon-P polyvinylidene difluoride (PVDF) membrane (Millipore) at 100 V for 1 to 2 hours at 4°C. Membranes were blocked for 1 hour and probed with 5% nonfat dry milk with the following primary antibodies: anti-IRF8 [clone D20D8, catalog no. 5628, Cell Signaling Technology and clone E-9, catalog no. sc-365042, Santa Cruz Biotechnology)], anti-CD74 (sheep anti-mouse cd74, polyclonal antibody, catalog no. AF7478, R&D Systems), anti-PU1 (clone 9G7, catalog no. 2258, Cell Signaling Technology), anti-FLAG (clone 9A3, catalog no. 8146, Cell Signaling Technology), anti–MHC class II (clone LGII-612.14, catalog no. 68258, Cell Signaling Technology), anti-CIITA antibodies (rabbit polyclonal, catalog no. 3793, Cell Signaling Technology, and mouse monoclonal, clone 7-1H, sc-13556, Santa Cruz Biotechnology), and anti–β-actin (#A2228, Sigma-Aldrich). Membranes were developed with the SuperSignal West Pico PLUS Chemiluminescent Substrate (Thermo Fisher Scientific) and captured on x-ray film. PVDF membranes were stripped with OneMinute Western blot Stripping Buffer (#GM6001, GM Biosciences) and reprobed with relevant antibodies for loading control.

### ChIP-qPCR assay

A20 B cell lymphoma cells, expressing IRF8 WT, IRF8 N87Y, or IRF8 I424T were fixed, 1% formaldehyde (Thermo Fisher Scientific, catalog no. 28908) for 10 min at room temperature and neutralized with 125 mM glycine (Thermo Fisher Scientific, catalog no. BP381-5) for 5 min, followed by washes in cold PBS. The cell pellet was resuspended in 500 μl of SDS lysis buffer [50 mM tris (pH8.0), 1% SDS, and 10 mM EDTA] with 10 μl of 50× protease inhibitor (Roche, catalog no. 04693116001). Chromatin was sheared on the ice using QSONICA Sonicator (model #431C2, Qsonica, LLC). The sonicated mix was centrifuged at 15,000*g* for 10 min, and the supernatant diluted at 1:10 to ChIP dilution buffer [16.7 mM tris-HCl (pH 8.1), 167 mM NaCl, 1.1% Triton X-100, and 1.2 mM EDTA] with protease inhibitor, and incubated for 16 hours at 4°C with an anti-IRF8 antibody [ICSBP (E-9) X, mouse monoclonal IgG, catalog no. Sc-365042X, Santa Cruz Biotechnology] or normal mouse IgG (Santa Cruz Biotechnology), prebound to Dynabeads protein G (catalog no. 10003D, Invitrogen). The immunoprecipitation mixture beads were pulled down using a magnet, supernatant removed, followed by sequentially washing with low salt immune complex wash buffer [20 mM tris (pH 8.0), 150 mM NaCl, 1% Triton X-100, and 2 mM EDTA], high-salt immune complex wash buffer [20 mM tris (pH 8.0), 500 mM NaCl, 1% Triton X-100, and 2 mM EDTA], and LiCl immune complex wash buffer [10 mM tris (pH 8.0), 0.25 M LiCl, 1% NP-40, 1% deoxycholic acid, and 1 mM EDTA], and TE [10 mM tris (pH 8.0) and 1 mM EDTA]. The beads were next incubated in 0.1 M NaHCO_3_ and 1% SDS for 30 min at room temperature to elute the chromatin DNA. Cross-link reversion was performed at 65°C for 4 hours, and proteins were removed using proteinase K at 45°C for 1 hour. The naked DNA was extracted, treated with ribonuclease A, and recovered in molecular biology grade water. qPCR was performed in Applied Biosystems Quantstudio 5 Real-Time PCR System Thermal Cycling Block (Applied Biosystems) in 10 μl of reactions. Primer sequences are listed in table S8.

### Immunohistochemistry of murine lymphomas

Xenograft tissue specimens were fixed in formalin, processed, and embedded in paraffin, and 4-μm serial sections were obtained. One section was stained with hematoxylin and eosin and the slides were reviewed by the pathologists (K.N.H. and J.P.R.) to confirm the presence of malignant lymphoma. Automated immunohistochemical stains were performed on Roche Ventana Discovery (Roche Diagnostics). B220 and CD3 dual stain was performed following antigen retrieval in CC1, with rat monoclonal anti-B220 (BD Biosciences clone RA3–6B2, 1:200) and detection using Discovery detection and Discovery Purple chromogen (Roche Diagnostics), followed by rabbit monoclonal anti-CD3e (Thermo Fisher Scientific/Invitrogen, clone SP7, 1:200) and detection using Discovery HRP and 3,3'-diaminobenzidine (DAB) chromogen (Roche Diagnostics). Single CD3 stain was performed following antigen retrieval in CC1, with rabbit monoclonal anti-CD3e (Thermo Fisher Scientific/Invitrogen, clone SP7, 1:200) and detection using Discovery HRP and DAB chromogen (Roche Diagnostics). All immunohistochemical sections were counterstained with hematoxylin. Dual-stained slides confirmed the B cell nature of the B220^+^ lymphoma cells (purple) which were infiltrated by variable numbers of CD3^+^ T cells (brown/DAB). For optimal resolution, CD3^+^ T cells were counted on CD3 single stained slides. CD3^+^ T cells were enumerated in a blinded fashion by the pathologists (KNH and JPR) by counting all CD3^+^ T cells in representative 40× high-power fields (×400 magnification) in areas which demonstrated sheet-like growth of lymphoma cells without intervening necrosis, fibroadipose tissue, or skeletal muscle. Data are reported as the average number of CD3^+^ T cells per 40× high-power field.

### Protein immunoprecipitation

Murine 2PK-3 B cell lymphoma cells were lysed in ice-cold NP-40 or RIPA lysis buffer completed with Halt protease & Phosphatase inhibitor cocktail (Thermo Fisher Scientific catalog no. 1861281). Protein (1 mg) was incubated at 4C overnight with anti-CIITA (clone 7-1H, mouse monoclonal antibody, catalog no. SC-13556, Santa Cruz Biotechnology) or iso-species mouse control IgG (catalog no. SC-2025, Santa Cruz Biotechnology), followed by 1 hour of incubation at room temperature with Dynabeads Protein G beads (Invitrogen catalog no. 10003D). The complex antigen/antibody/beads was washed three times with NP40 or RIPA lysis buffer at 4°C, eluted with 5× Laemmli buffer by boiling at 100°C for 10 min, and separated on SDS-PAGE, and immunoblotted ON at 4°C with the anti-CIITA antibody (rabbit polyclonal antibody, catalog no. 3793, Cell Signaling Technology).

### In vitro cell growth assay

Models of human DLBCL cell lines (Toledo, SU-DHL4, SU-DHL6, SU-DHL2, and RIVA) and mouse B cell lymphoma cell lines (A20, 2PK-3, and BCL1), expressing an empty control vector, harboring IRF8 KO, or expressing IRF8 WT and mutants (S55A, N87Y, D400G, and I424T), or with CD74 KO or its ectopic expression were analyzed for in vitro cell growth. In brief, cells were seeded at 5 × 10^5^/ml or 2.5 × 10^5^/ml in six-well plates in triplicate, and quantified daily, for 96 hours, using an automated cell counter (Cellometer K2 Fluorescent Cell Counter, Nexcellom). Each assay was performed with three biological replicates.

### RNA isolation and RT-PCR

Cells were harvested and total RNA was isolated using TRIzol (Invitrogen) as we described (*85*). The RNA integrity and purity was determined by gel electrophoresis and by measuring 260/280 and 260/230 ratios in a nanodrop spectrophotometer, and cDNA generate with the High-Capacity cDNA Reverse Transcription Kit (Applied Biosystems). Quantitative RT-PCR were performed in triplicate using iTaq Universal SYBR Green (Bio-Rad) on QuantStudio 5 real-time PCR system (Applied Biosystems). All reactions included a no-reverse transcriptase sample and no-template control (water) to control for genomic DNA amplification or contamination. TATA box–binding protein was used as the internal control. Relative gene expression was calculated using the 2^–ΔΔ^Ct method, as we reported (*86*). (Oligonucleotides sequences are listed in table S8.)

### Estimation of immune-cell fractions based on gene expression

To estimate the relative abundance of cell types that might be present in the bulk RNA-seq of the NCICCR-DLBCL tumor cohort, we used three computational tools based on enrichment or deconvolution of mixed sample population. First, we applied the xCell software, set to the default parameters, to generate enrichment scores for 64 immune and stromal cell types trained from a comprehensive database of reference signatures derived from over 1800 pure cell types to predict the enrichment of these immune cell types in a mixed sample population (*59*). Each cell type enrichment scores were then compared between IRF8 mutant or WT using two-sided Student’s *t* test. We also applied CIBERSORTx, a method based on support-vector regression for the deconvolution of 22 immune cell types using default settings (*64*). In addition, we estimated the relative proportions of immune cell types using the MCP-counter software, which is based on enrichment of marker genes and produces profiles of 11 cell types, including eight immune cells (*65*). Variations in the underlying methodology and reference cell types from these three algorithms provide complementary as well as supportive information.

### Structural modeling

For comparative structural analyses we used a crystal structure of IRF3 DBD/DNA complex (PDB: 2PI0) and AlphaFold model of IRF8 full-length (AF-Q02556-F1). IRF3/DNA complex structure comprises four IRF3 DBDs bound to a complete PRDIII-1 regulatory element of the human IFN-β enhancer. We superposed the DBD of IRF8 over one of the four DNA-bound DBDs of IRF3 in Coot (*87*). The structure figures were prepared using Pymol (Schrodinger Suite).

### Quantification and statistical analysis

Analyses were performed using a one-way analysis of variance (ANOVA), with Bonferroni’s multiple comparison or Fisher’s LSD post hoc test, two-tailed Student’s *t* test, and Mann-Whitney test. *P* < 0.05 was considered significant. Data analyses were performed in the Prism 9 software (version 9.4.1, GraphPad Software Inc.) and Excel (Microsoft 365 – Office).

## Supplementary Materials

### This PDF file includes:

Figs. S1 to S38 Tables S1 to S8 References
