# Supplementary material for: IRF8-mutant B cell lymphoma evades immunity through a CD74-dependent deregulation of antigen processing and presentation in MHCII complexes
Source: Sci Adv. 2024 Jul 12;10(28):eadk2091. doi: 10.1126/sciadv.adk2091 (PMC11244530; doi:10.1126/sciadv.adk2091)
Supplement: Supplementary file 1 — Figs. S1 to S38 Tables S1 to S8 References [file sciadv.adk2091_sm.pdf]

Science Advances
AAAS

Supplementary Materials for

IRF8-mutant B cell lymphoma evades immunity through a CD74-dependent deregulation of antigen processing and presentation in MHCII complexes

Zhijun Qiu *et al.*

Corresponding author: Ricardo C.T. Aguiar, aguiarr@uthscsa.edu

Sci. Adv. **10**, eadk2091 (2024)
DOI: 10.1126/sciadv.adk2091

This PDF file includes:

Figs. S1 to S38
Tables S1 to S8
References

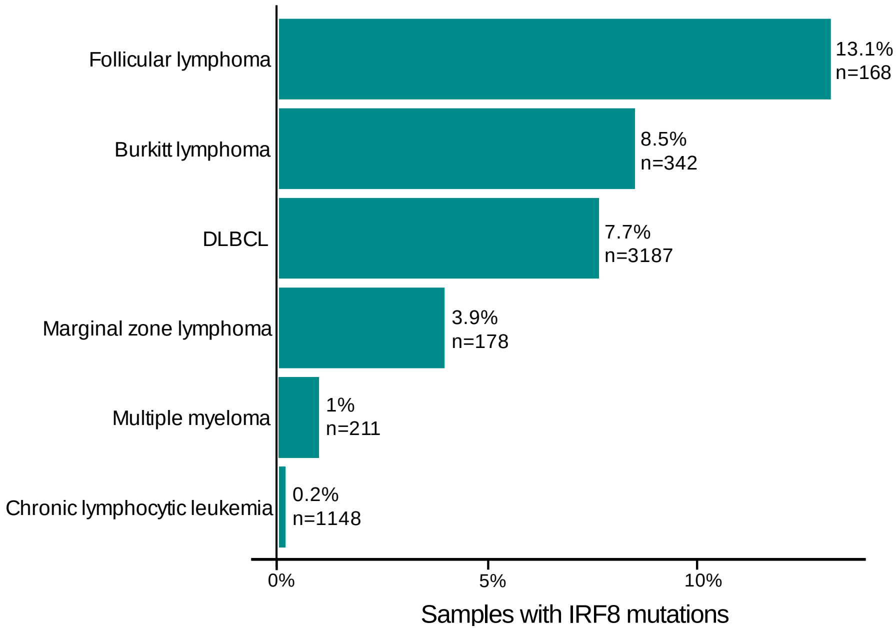

Figure S1. Frequency of *IRF8* variants in mature B-cell malignancies. Data are from publicly available cohorts, shown are the size of the cohorts and % of *IRF8* gene mutations (related to Supplemental Tables 1, 5 and 6).

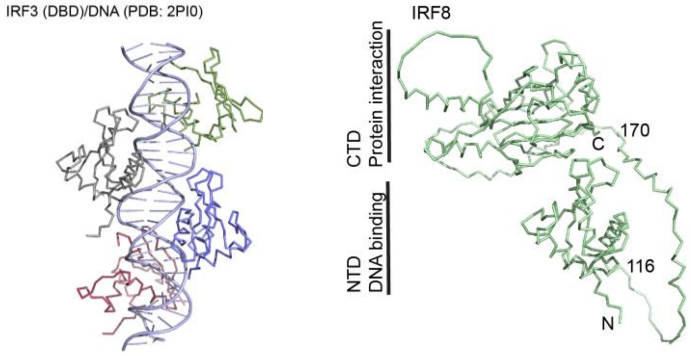

Figure S2. Computational modeling of human IRF8. Left - crystal structure of IRF3 DBD/DNA complex (PDB: 2PI0); **right** - alphaFold model of full-length IRF8. CTD, c-terminus domain; DBD, DNA-binding domain.

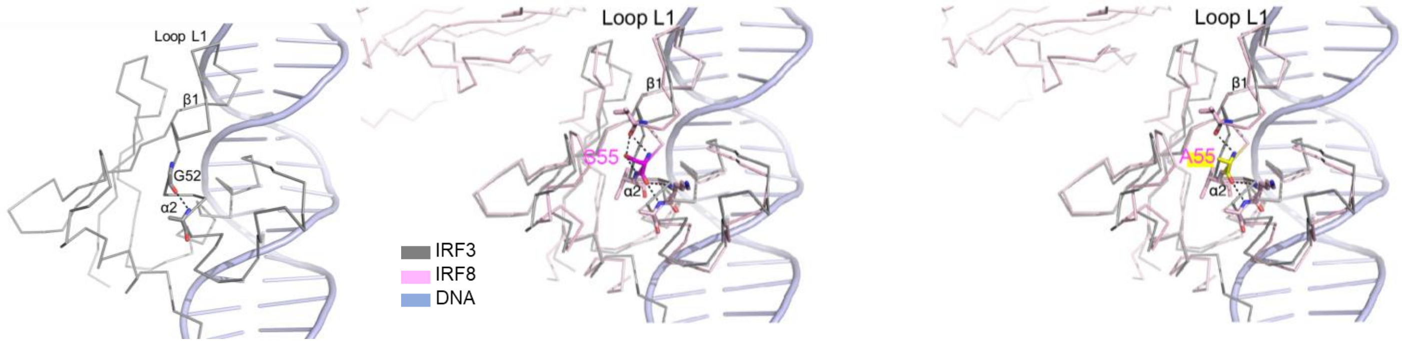

Figure S3. Modeling of IRF8 mutation S55A. Left, solved structure of IRF3/DNA, displaying aa G52, equivalent to S55 in IRF8**. Middle,** overlay of the DNA binding domains of IRF3 (grey) and IRF8 (pink) with close-up in the S55 residue and its relation to the α2 helix of IRF8's DBD and loop1 between α2 and β1 in contact with DNA. **Right,** A55 substitution, which may result in loss of the stabilizing interaction between the amino acid side chain and loop1.

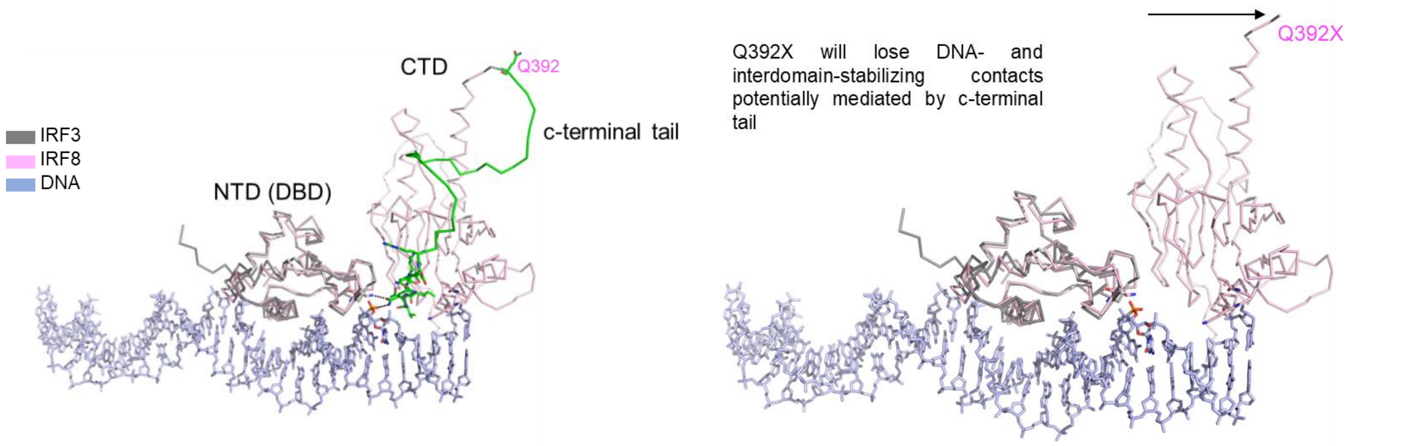

Figure S4. Modeling of IRF8 mutation Q392X. Left, an overlay of DBDs of IRF3 (PDB: 2PI0, grey, protein; blue, DNA) and IRF8 (AlphaFold model, pink) showing Q392 (green stick) and c-terminal tail and its putative contact with DNA. **Right,** an overlay of DBDs of IRF3 DNA and IRF8 showing Q392 truncation and potential loss of DNA and interdomain-stabilizing contacts mediated by c-terminal tail

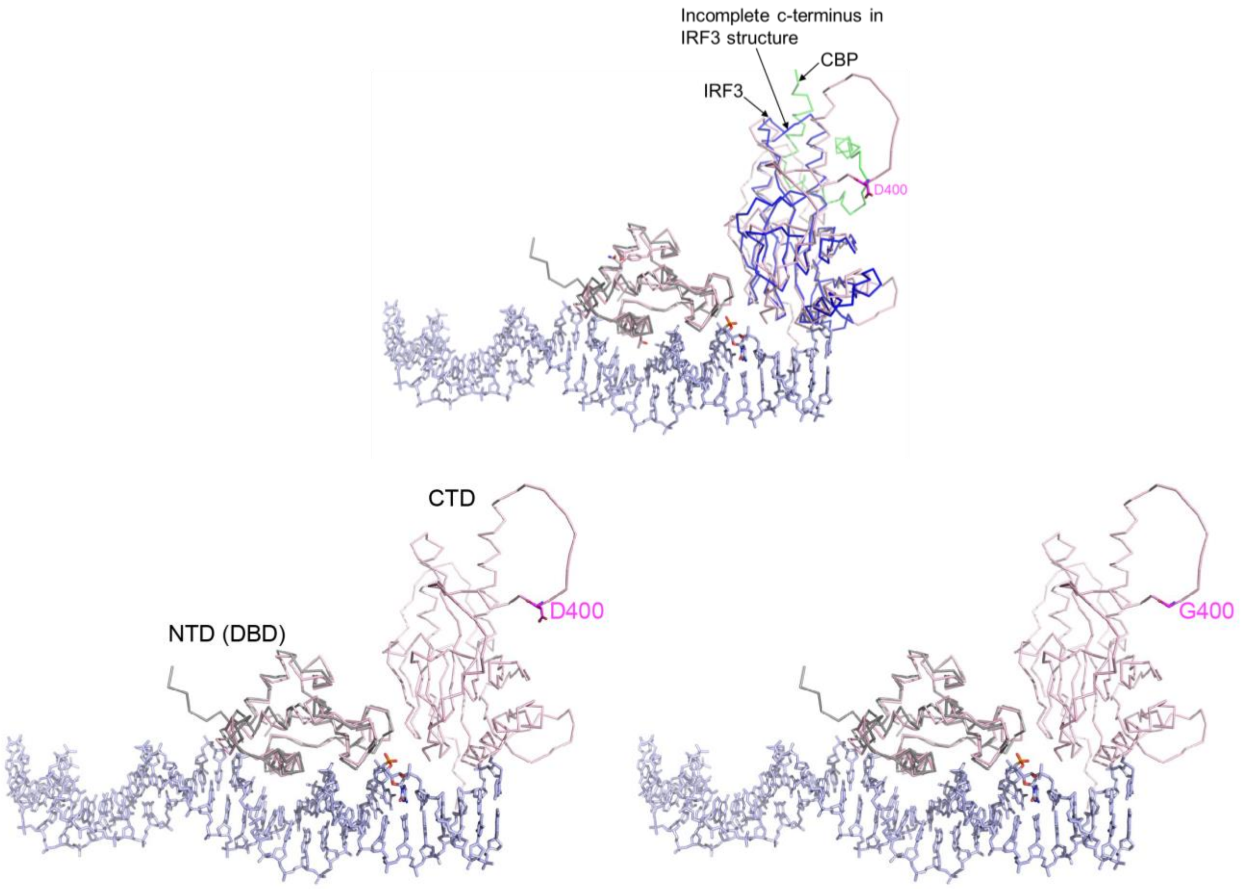

Figure S5. modeling of IRF8 mutation D400G. Top - overlay of the CTDs of IRF3 (blue ribbons) with IRF3's CTD/CBP complex (PDB: 1ZOQ) and IRF8 (pink). The IRF3 CTD lacks the outer loop and c-terminal tail regions in the crystal structure. **Bottom** - an overlay of DBDs of IRF3 (PDB: 2PI0, grey, protein; blue, DNA) and IRF8 (AlphaFold model, pink) showing D400 (magenta stick) (**left**) or G400 (**right**) in an "outer loop".

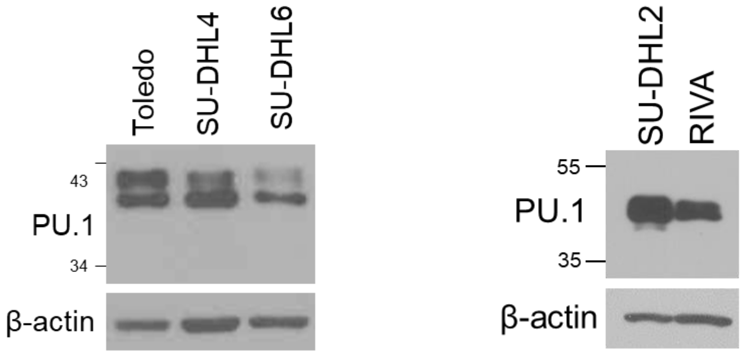

Figure S6. **PU.1 expression.** Western blot analysis of PU.1 expression in human DLBCL cell lines.

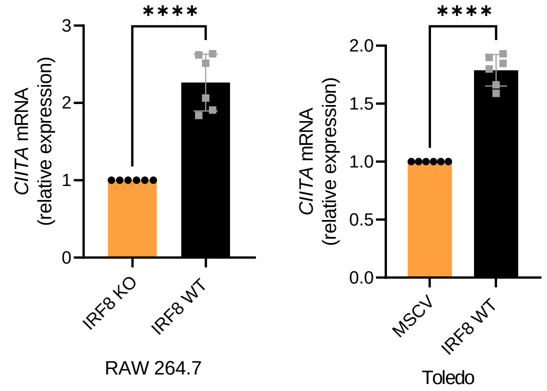

Figure S7. ***CIITA* q-RT-PCR.** *CIITA* mRNA expression in cell lines with IRF8 KO (RAW 264.7, left) or lacking endogenous IRF8 expression (Toledo, right), followed by stable ectopic expression of human IRF8 WT. Data are mean ± SD of a total of four biological replicates; P values are from two-sided Student's t-test (**** = <0.0001).

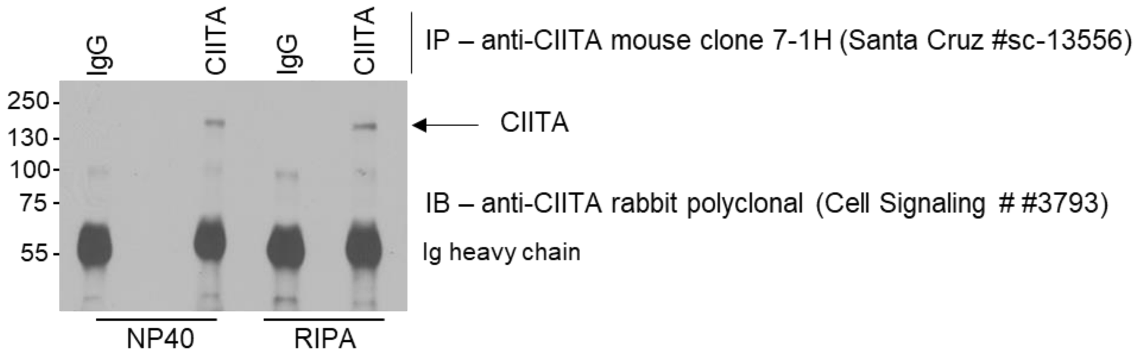

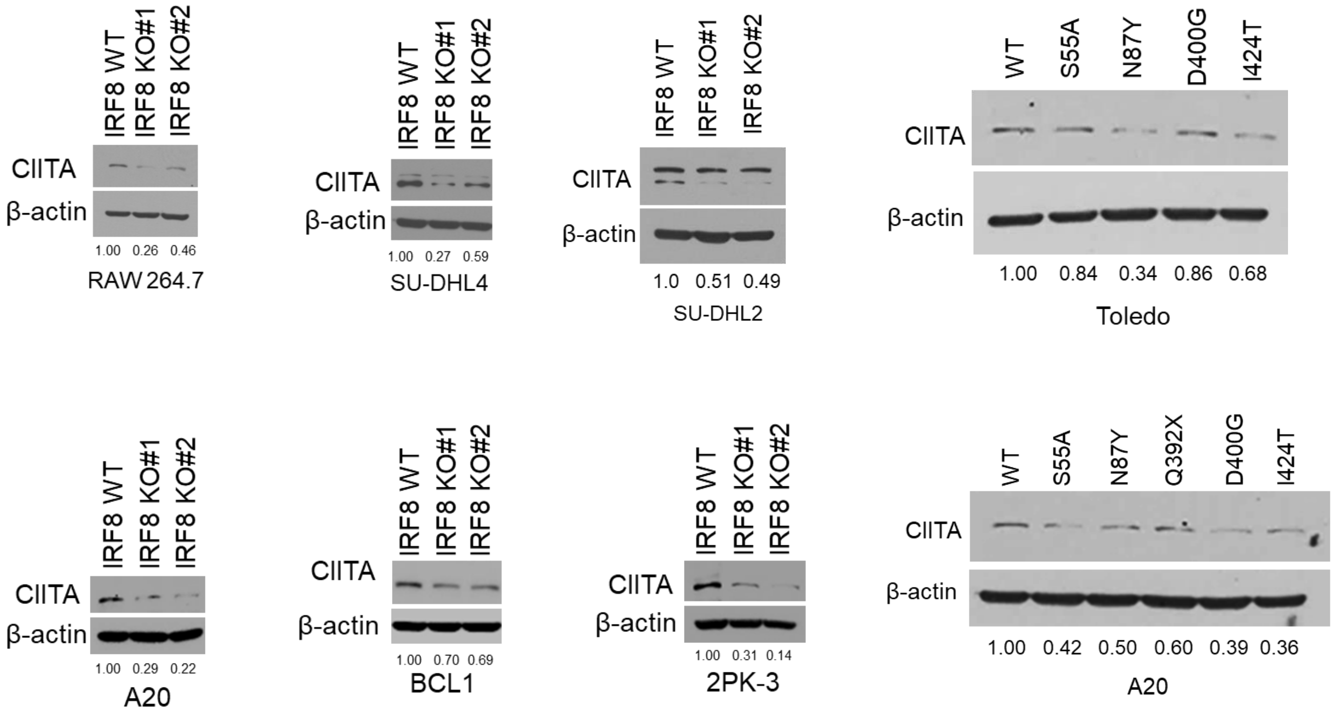

Figure S8. CIITA western blots. Top, immunoprecipitation (IP) of CIITA followed by immunoblotting (IB) with indicated antibodies. Protein isolated from 2PK-3 cell line with two distinct cell lysis buffers (NP-40 or RIPA). **Mid and bottom panels**. Detection CIITA protein levels in multiple genetic models of IRF8 in mouse and human cell lines. Densitometric quantification of CIITA expression is also show.

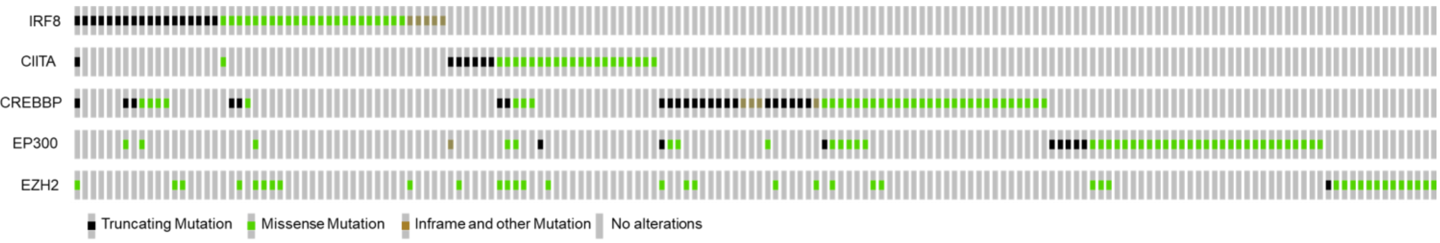

A	B	Total	Neither	A Not B	B Not A	Both	Log2 Odds Ratio	p-Value	q-Value	Tendency
IRF8	CIITA	168	96	44	26	2	-2.6	0.009	0.04	Mutual exclusivity
IRF8	CREBBP	168	69	36	53	10	-1.467	0.012	0.04	Mutual exclusivity
IRF8	EP300	168	74	43	48	3	<-3	<0.001	<0.001	Mutual exclusivity
IRF8	EZH2	168	91	37	31	9	-0.486	0.543	0.679	Mutual exclusivity

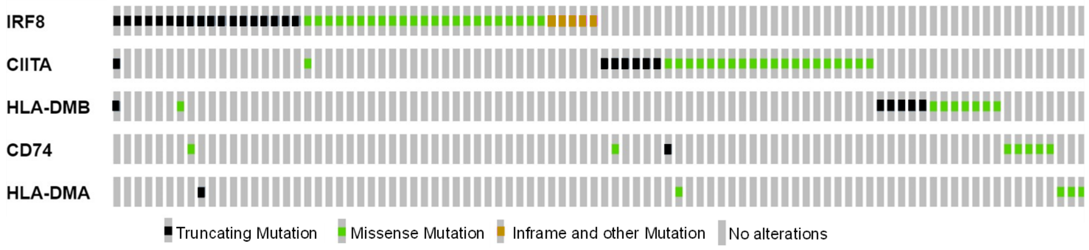

A	B	Total	Neither	A Not B	B Not A	Both	Log2 Odds Ratio	p-Value	q-Value	Tendency
IRF8	CIITA	92	20	44	26	2	<-3	<0.001	<0.001	Mutual exclusivity
IRF8	HLA-DMB	92	34	44	12	2	-2.957	0.007	0.036	Mutual exclusivity
IRF8	CD74	92	39	45	7	1	<-3	0.032	0.107	Mutual exclusivity
IRF8	HLA-DMA	92	42	45	4	1	-2.1	0.361	0.722	Mutual exclusivity

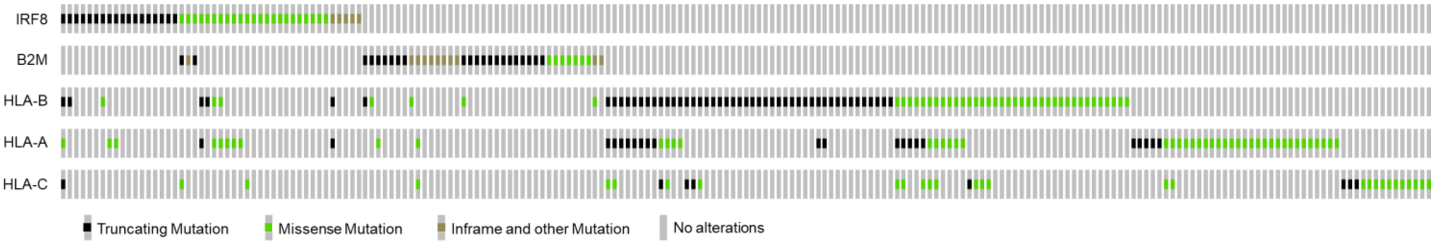

A	B	Total	Neither	A Not B	B Not A	Both	Log2 Odds Ratio	p-Value	q-Value	Tendency
IRF8	B2M	209	126	43	37	3	-2.073	0.011	0.028	Mutual exclusivity
IRF8	HLA-B	209	78	38	85	8	-2.372	<0.001	<0.001	Mutual exclusivity
IRF8	HLA-A	209	104	36	59	10	-1.03	0.077	0.11	Mutual exclusivity
IRF8	HLA-C	209	130	43	33	3	-1.863	0.028	0.047	Mutual exclusivity

Figure S9. **Mutual exclusivity of IRF8 mutation in DLBLC**. **Top** - Oncoprint display of the distribution of IRF8, CIITA, CREBBP, EP300 and EZH2 (genes previously reported to decrease MHC class II expression) mutations in 168 DLBCLs; pairwise log2 odds score, as well as P and Q values of the correlation between the co-occurrence (positive score) or the mutually exclusive presence of mutation (negative score), are shown in a table format. **Middle –** Oncopritnt display of the distribution of IRF8, CIITA, CD74, HLA-DMA and HLA-DMB (MHCII "specific" genes) mutations in 92 DLBCLs; pairwise log2 odds score, as well as P and Q values of the correlation between the co-occurrence (positive score) or mutually exclusive mutation (negative score), are shown in a table format. **Bottom** - Oncoprint display of the distribution of IRF8, B2M, HLA-A, HLA-B and HLA-C (MHCI genes) mutations in 209 DLBCLs; pairwise log2 odds score, as well as P and Q values of the correlation between the co-occurrence (positive score) or mutually exclusive mutation (negative score), are shown in table format. Symbols are color coded for the type of mutation. p or q value <0.05 was considered significant.

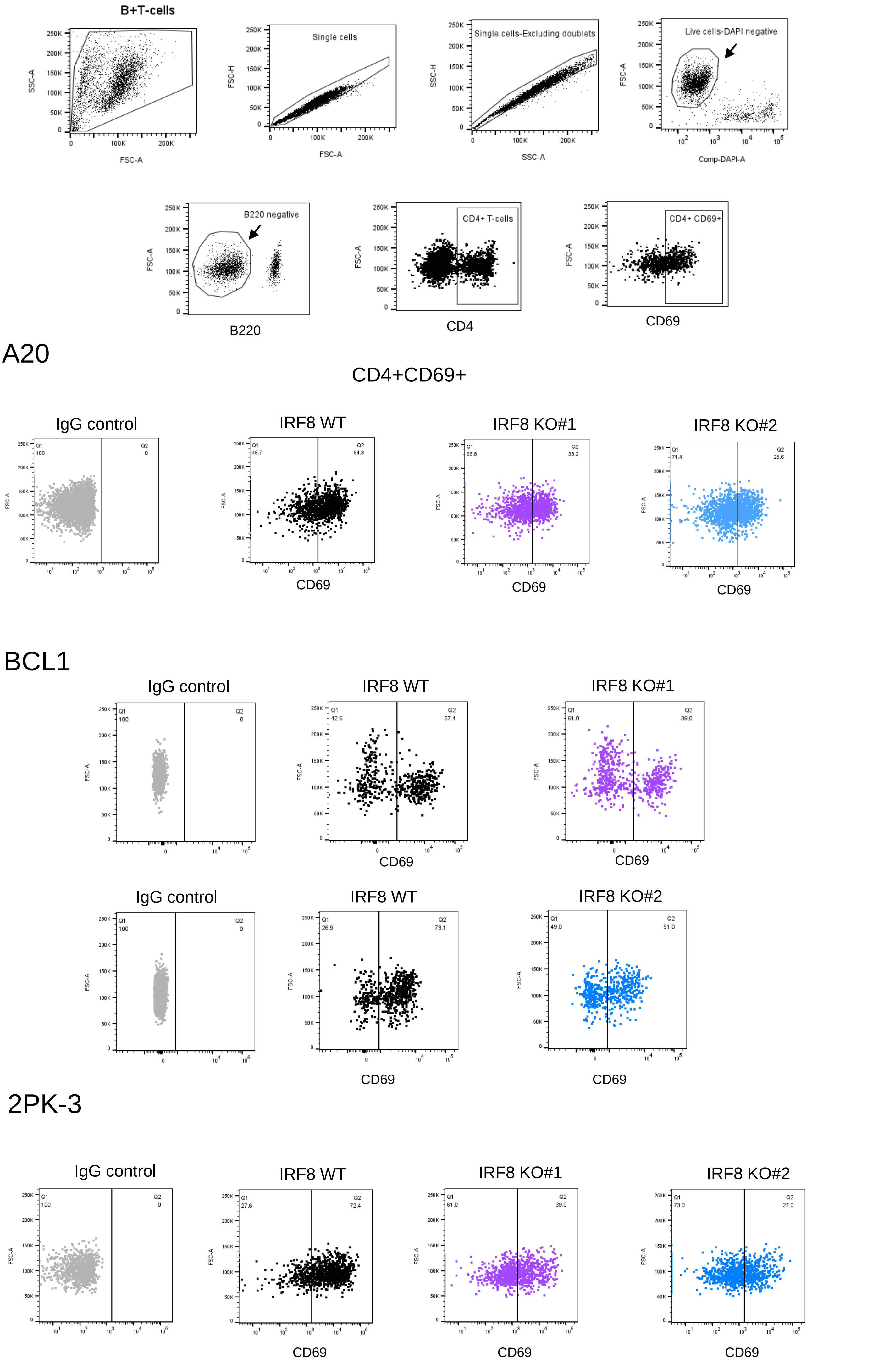

Figure S10. FACS analysis of CD69. Gating strategy and representative displays of CD69 quantification by FACS in CD4+ DO-11.10 murine cells following antigen (OVA) presentation by three B cell lymphoma cells (A20, BCL1, 2PK-3), WT or KO for IRF8 (2 independent guide RNAs, two independent KO clones).

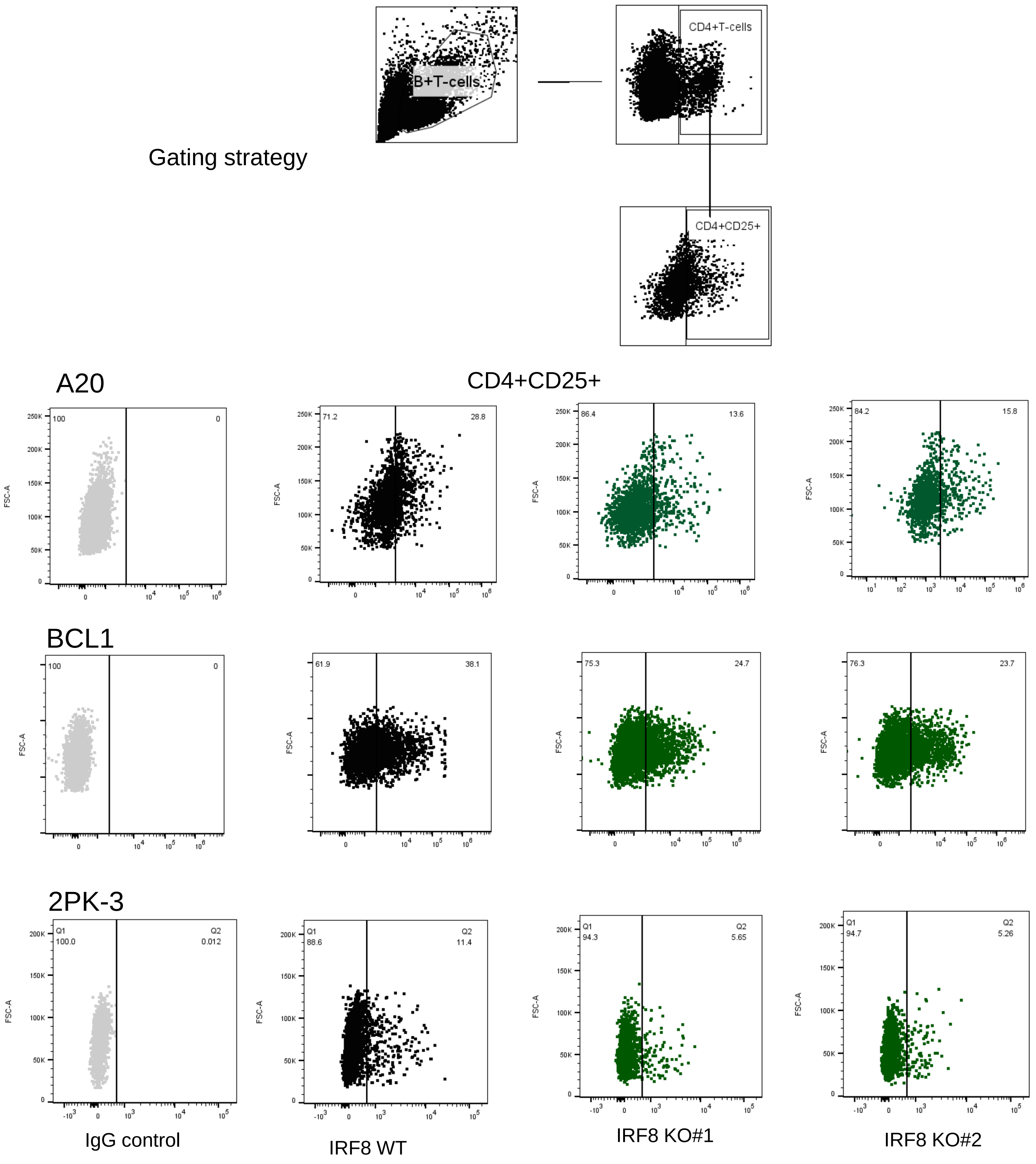

Figure S11. **FACS analysis of CD25**. Gating strategy and representative displays of CD25 quantification by FACS in CD4+ DO-11.10 murine cells following antigen (OVA) presentation by three B cell lymphoma cells (A20, BCL1, 2PK-3), WT or KO for IRF8 (2 independent KO clones).

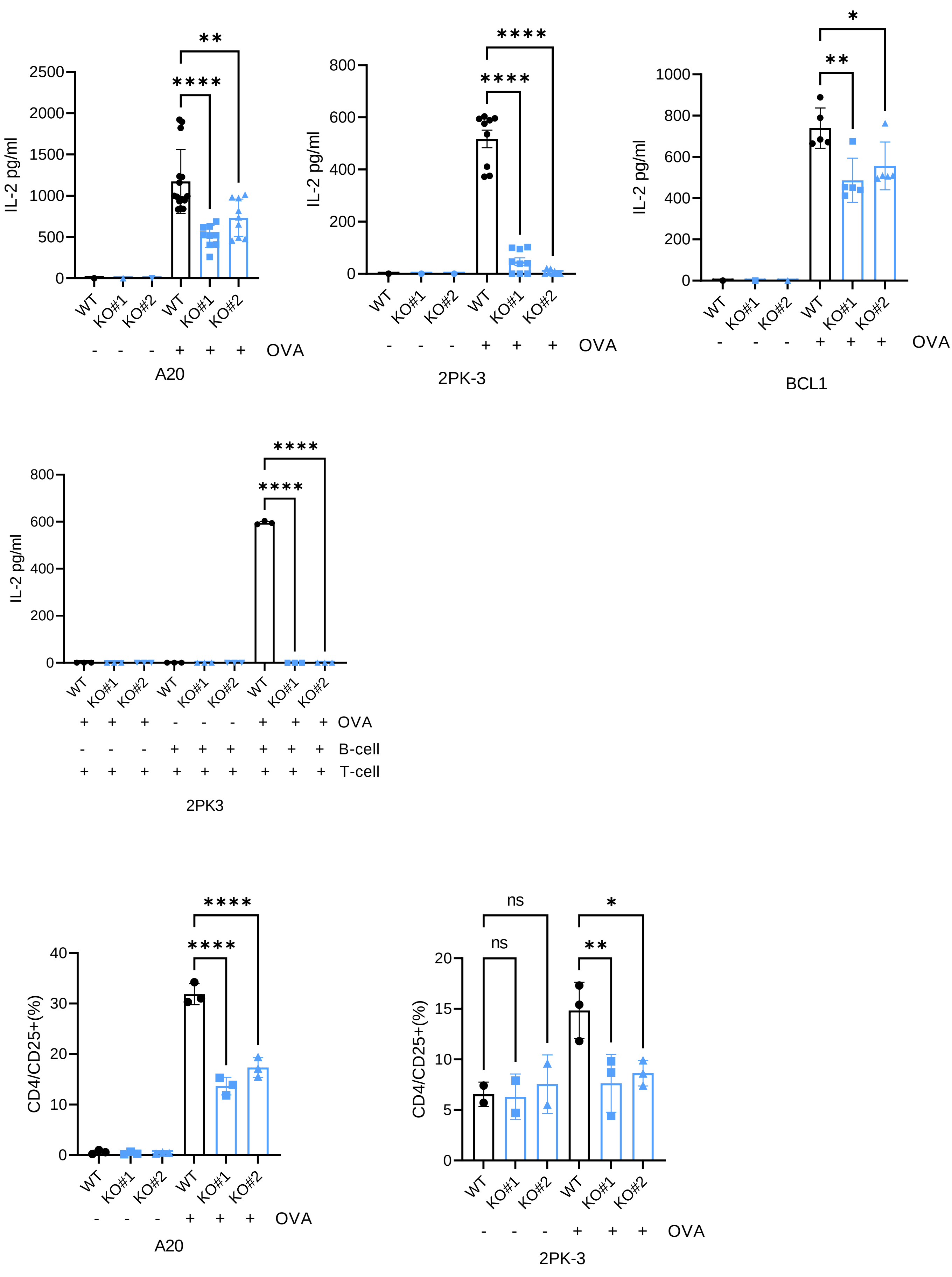

Figure S12. **Activation of CD4+ DO-11.10 cells. Top panels**. IL-2 quantification in conditioned media of CD4+ DO-11.10 cells co-cultured with mouse B cell lymphoma cell lines A20, BCL1 and 2PK-3, each IRF8 WT or KO, "loaded" or not with OVA. **Middle panel**. IL-2 quantification in conditioned media of CD4+ DO-11.10 cells exposed to IL-2 but not co-cultured with 2PK-3 lymphoma cells, or in conditioned media of CD4+ DO-11.10 cells co-cultured with the mouse B cell lymphoma cell line 2PK-3, IRF8 WT or KO, "loaded" or not with OVA. **Bottom panels.** CD25 quantification by FACS in CD4+ DO-11.10 murine cells co-cultured with mouse B cell lymphoma cell lines A20 and 2PK-3, each IRF8 WT or KO, "loaded" or not with OVA. Data are mean ±SD of two to three biological replicates, performed with one, two or three technical replicates. P values are from one ANOVA with Bonferroni post-test; , *(p<0.05), **(p<0.01), ***(p<0.001), **** (p<0.0001).

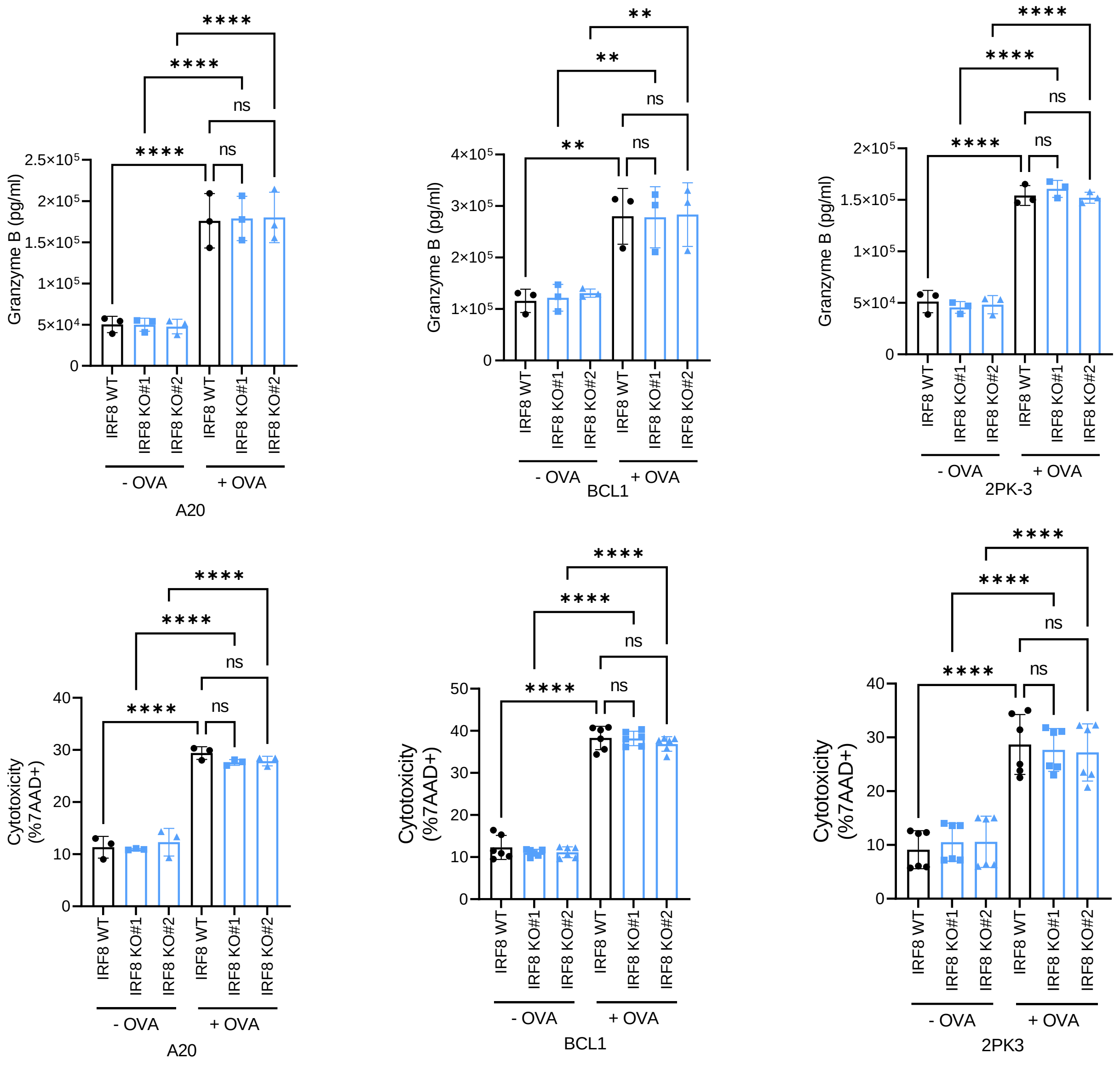

Figure S13. **Activation of CD8 OT-I cells. Top panels**. ELISA-based granzyme quantification in conditioned media of CD8 OT-I cells co-cultured with mouse B cell lymphoma cell lines A20, BCL1 and 2PK-3, each IRF8 WT or KO, “loaded” or not with OVA. **Bottom panels**. FACS-based quantification of 7AAD+ lymphoma cells (A20, BCL1, or 2PK-3), IRF8 WT or KO, “loaded” or not with OVA**.** . Data are mean ±SD of three biological replicates, performed with one, two or three technical replicates. P values are from one-way ANOVA with Bonferroni post-test; , *(p<0.05), **(p<0.01), ***(p<0.001), **** (p<0.0001).

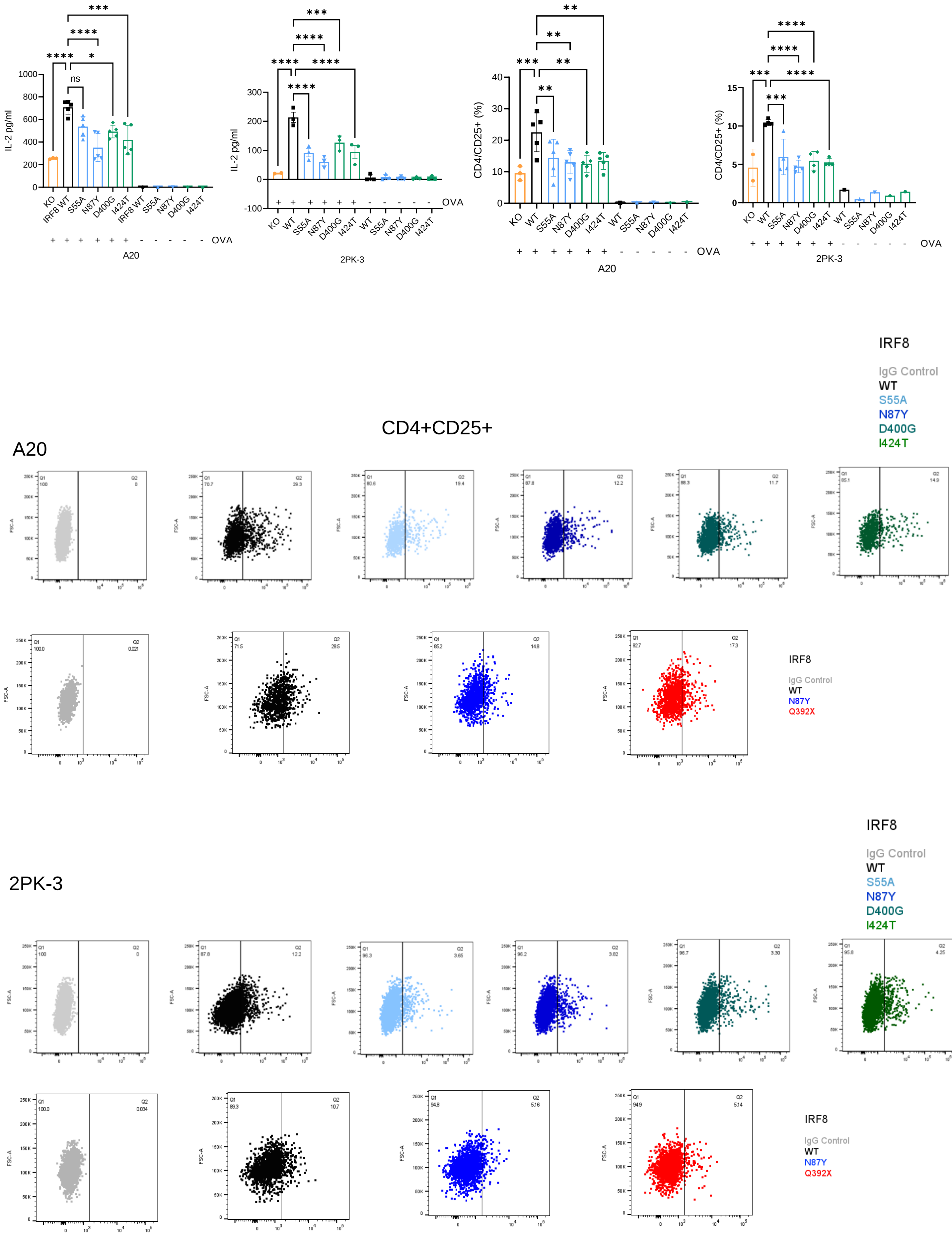

Figure S14. **Activation of CD4+ DO-11.10 cells. Top panels, left to right**. IL-2 quantification in conditioned media of CD4+ DO-11.10 cells co-cultured with mouse B cell lymphoma cell lines A20 (left) or 2PK-3 (right), each expressing IRF8 WT or mutant, “loaded” or not with OVA. CD25 quantification by FACS in CD4+ DO-11.10 murine cells co-cultured with mouse B cell lymphoma cell lines A20 (left) or 2PK-3 (right), each expressing IRF8 WT or mutant, “loaded” or not with OVA. Data are mean ±SD of three biological replicates, performed with one, two or three technical replicates. P values are from one ANOVA with Bonferroni post-test; *(p<0.05), **(p<0.01), ***(p<0.001), **** (p<0.0001). **Middle and Bottom panels.** Representative displays of CD25 quantification by FACS in CD4+ DO-11.10 murine cells following antigen (OVA) presentation by the B cell lymphoma cells A20 and 2PK-3, expressing IRF8 WT or the mutants S55A, N87Y, D400G and I424T, or A20 and 2PK-3, expressing IRF8 WT and the mutants N87Y and Q392X.

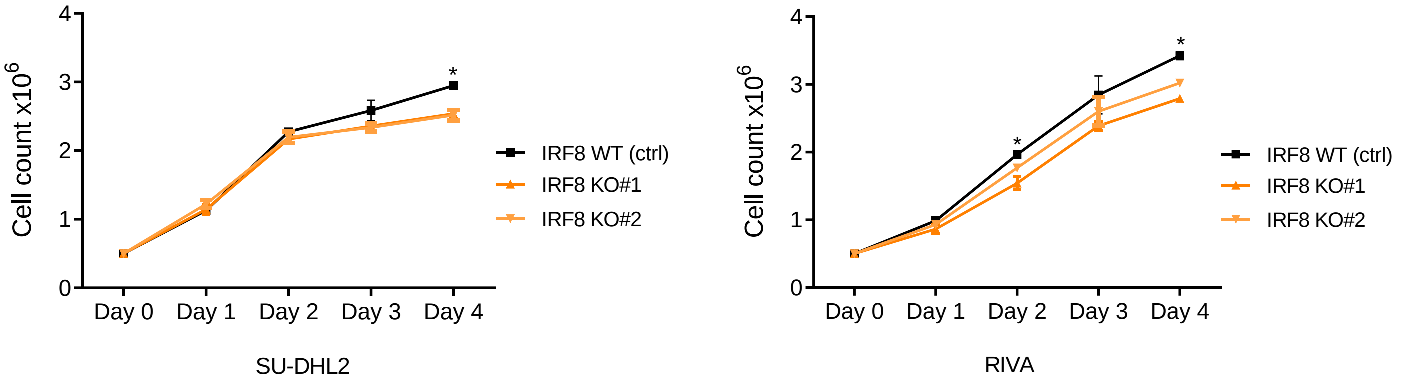

Figure S15. **Cell growth pattern of IRF8 KO DLBCL cell lines**. Growth profile of SU-DHL2 and RIVA determined with automated fluorescent cell counter (related to Figure 5A). Data are mean ± SD of two biological replicates, each performed in triplicate. P values, WT vs. KO were calculated with two-sided Student's t-test *(p<0.05).

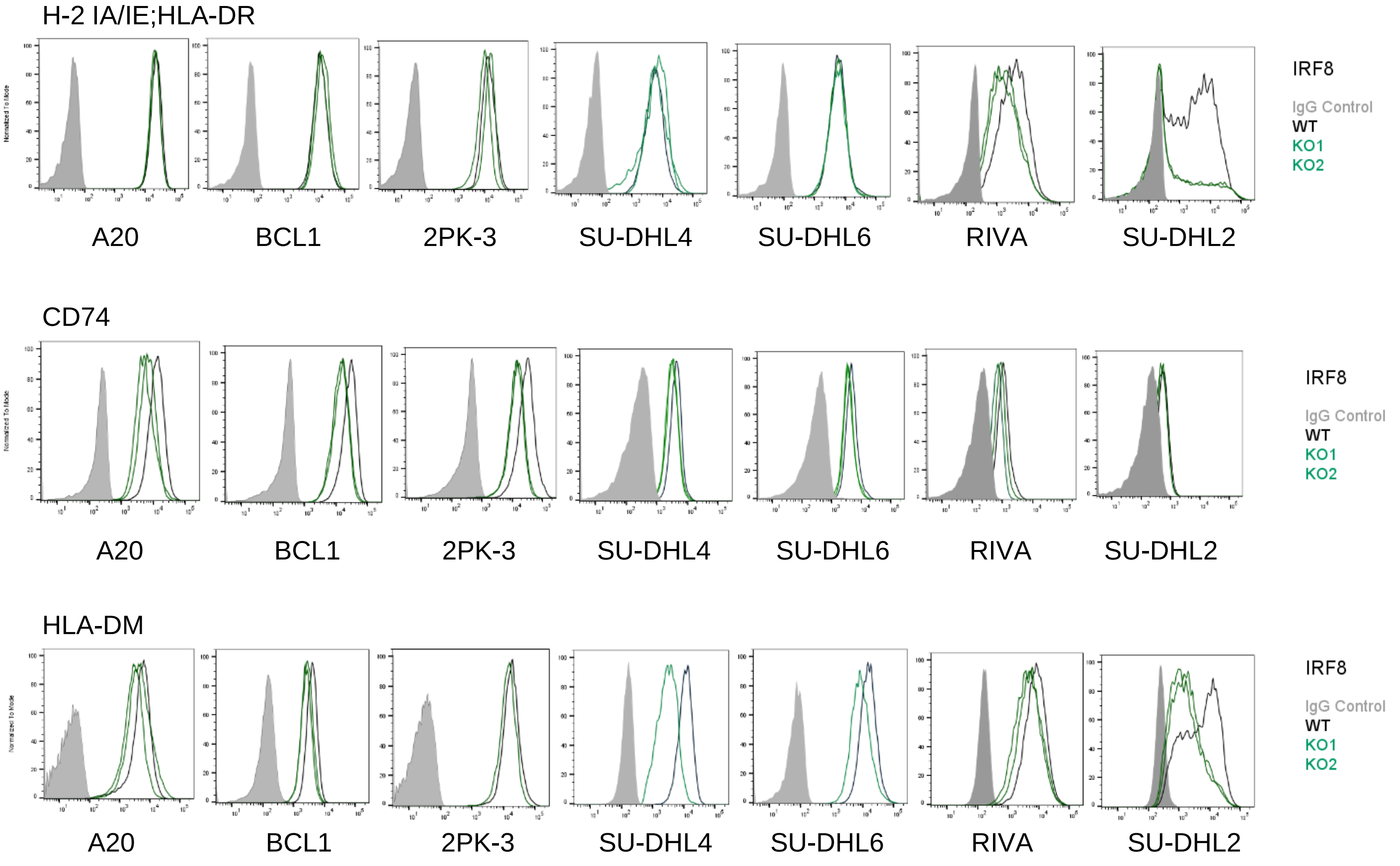

Figure S16. **Representative histograms.** FACS analysis of HLA-DR, CD74 and HLA-DM in IRF8 WT or KO mouse and human B cell lymphoma cell lines.

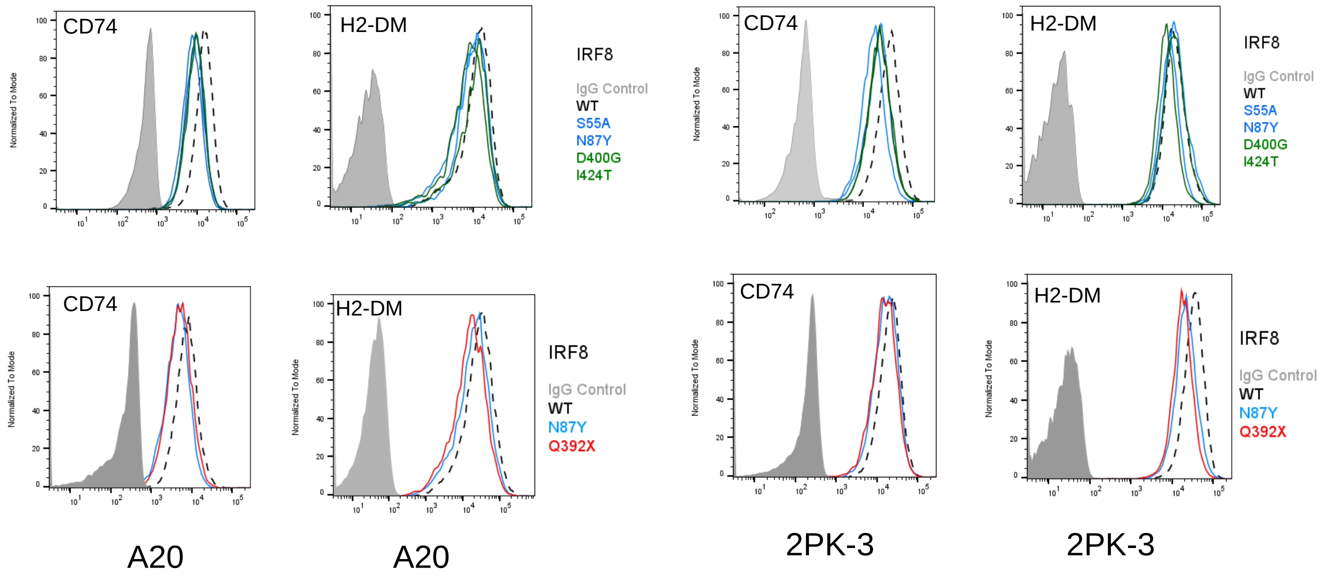

Figure S17. **Representative histograms.** FACS analysis of CD74 and HLA-DM in IRF8 KO murine B cell lymphoma cell lines A20 (left) and 2PK-3 (right) "rescued" with IRF8 WT, S55A, N87Y, Q392X, D400G and I424T mutants.

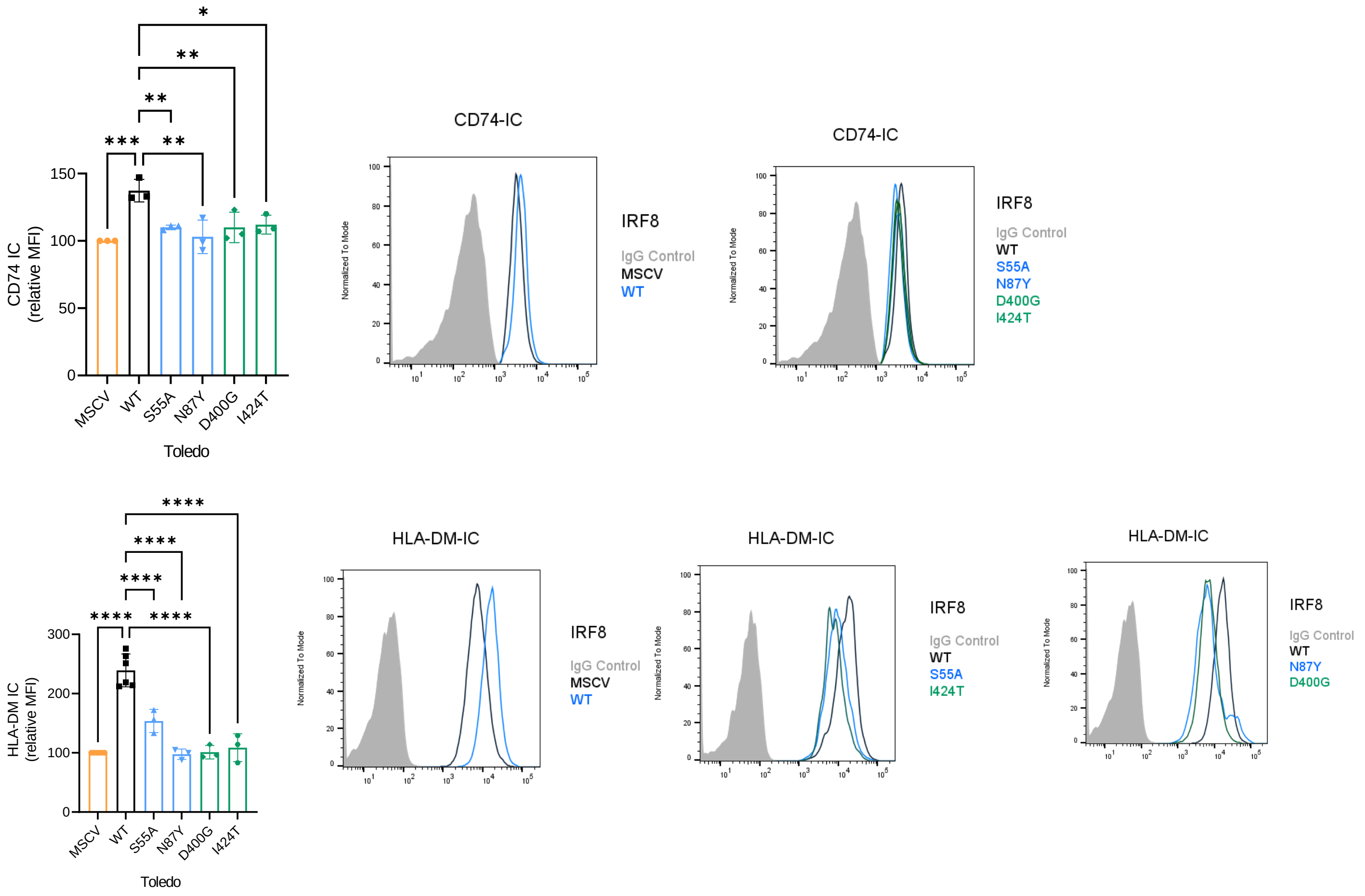

Figure S18. **FACS analysis of CD74 and HLA-DM in DLBLC cell lines.** FACS analysis of intra-cellular (IC) CD74 and HLA-DM in the human DLBCL cell line stably expressing an empty vector (MSCV), IRF8 WT or mutants (relative MFI, three biological replicates). Representative histograms are shown to the right. P values are from ANOVA, with Bonferroni post-test. *(p<0.05), **(p<0.01), ***(p<0.001), **** (p<0.0001).

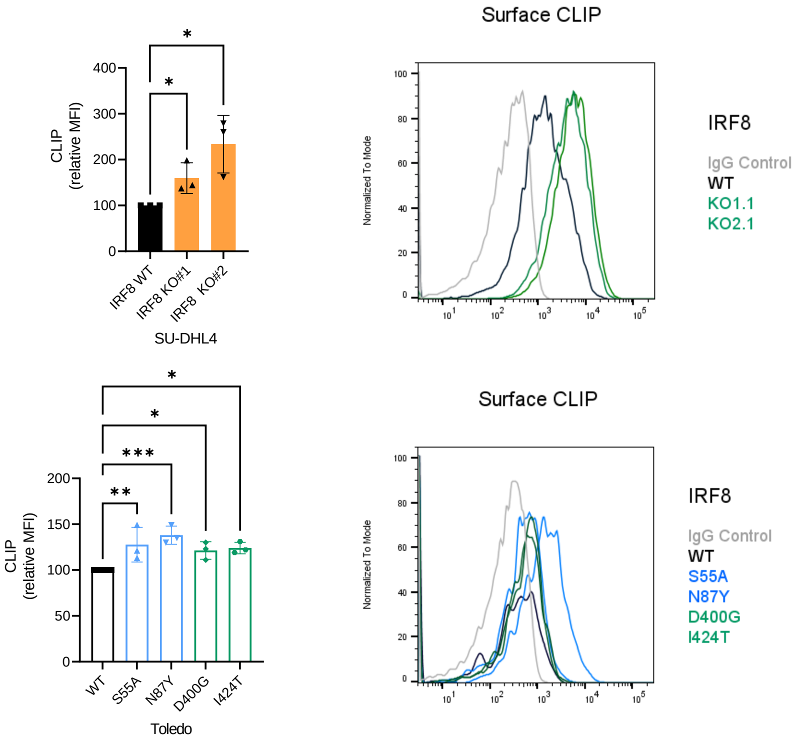

Figure S19. **FACS analysis of CLIP in DLBLC cell lines.** FACS analysis of CLIP expression in the cell surface of SU-DHL4 (IRF8 WT vs. KO) and Toledo (IRF8 WT vs mutant) (relative MFI, three biological replicates). Representative histograms are shown to the right.). P values are from two-sided Student's t-test (SU-DHL4) or one-way ANOVA, with Bonferroni post-test (Toledo), or. *(p<0.05), **(p<0.01), ***(p<0.001), **** (p<0.0001).

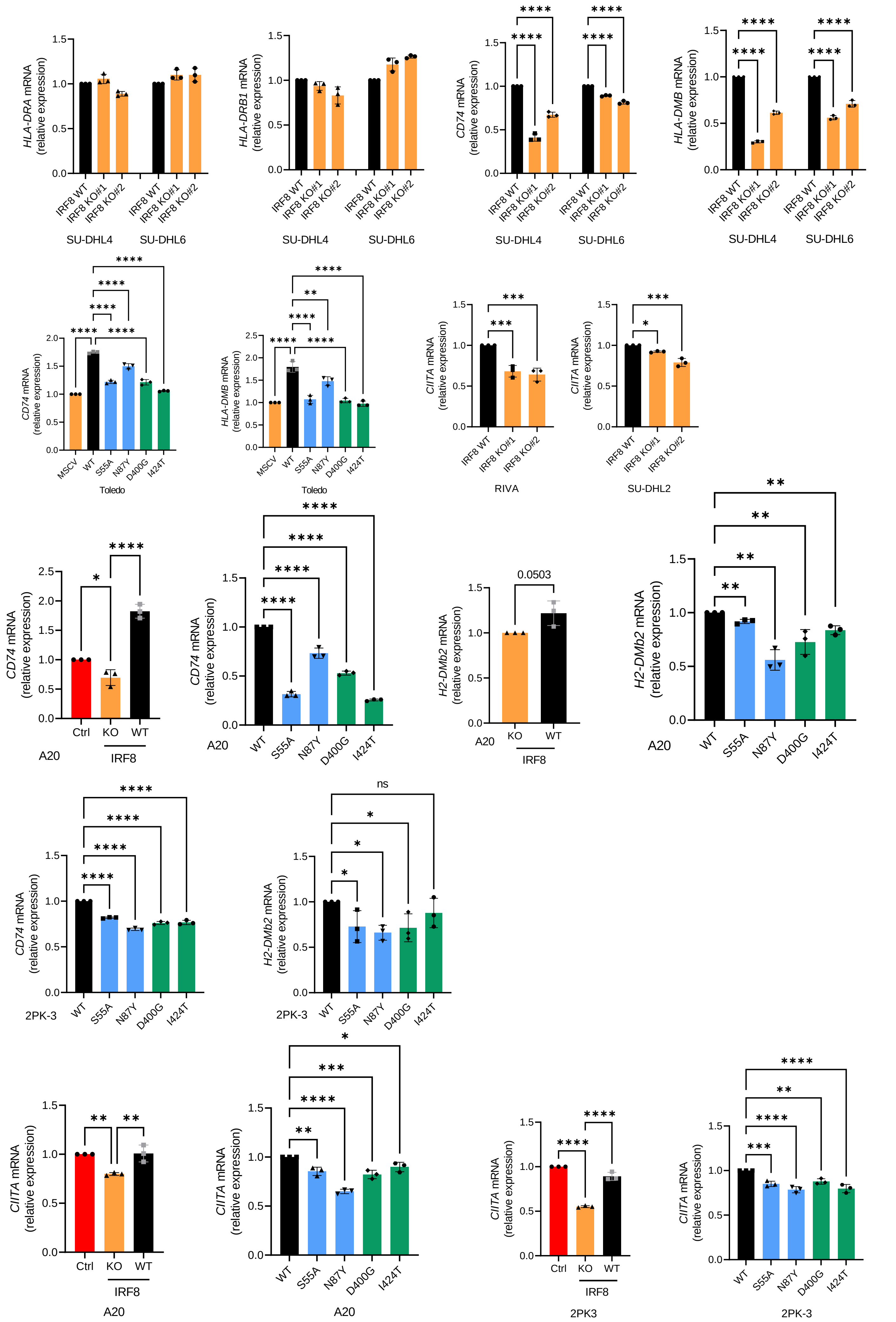

Figure S20. Q-RT-PCRs in IRF8 WT, KO or mutant DLBCL and mouse B cell lymphoma cell lines. Top to bottom. mRNA expression of *HLA-DRA*, *HLA-DRB1*, *CD74* and *HLA-DMB* in IRF8 WT or KO SU-DHL4 and SU-DHL6 cell lines. mRNA expression of *CD74* and *HLA-DMB* in Toledo cell line expressing an empty vector (MSCV), IRF8 WT or S55A, N87Y, D400G, I424T mutants. mRNA expression of *CIITA* in IRF8 WT or KO RIVA and SU-DHL2 cell lines. mRNA expression of *CD74* and *H2-DMb2* in A20 mouse B cell lymphoma cell line expressing an empty vector (ctrl), IRF8 KO, or IRF8 WT, S55A, N87Y, D400G, I424T mutants. mRNA expression of *CD74* and *H2-DMb2* in 2PK-3 mouse B cell lymphoma cell line expressing IRF8 WT, or mutants S55A, N87Y, D400G, I424T. mRNA expression of *CIITA* in A20 and 2PK-3 in mouse B cell lymphoma cell lines expressing an empty vector (ctrl), IRF8 KO, or IRF8 WT, S55A, N87Y, D400G, I424T mutants. Data are mean ±SD of three biological or technical replicates. P values are from one-way ANOVA with Bonferroni or Fisher's LSD post-test, or from two-tailed Student's t-test. *($p<0.05$), **($p<0.01$), ***($p<0.001$), **** ($p<0.0001$).

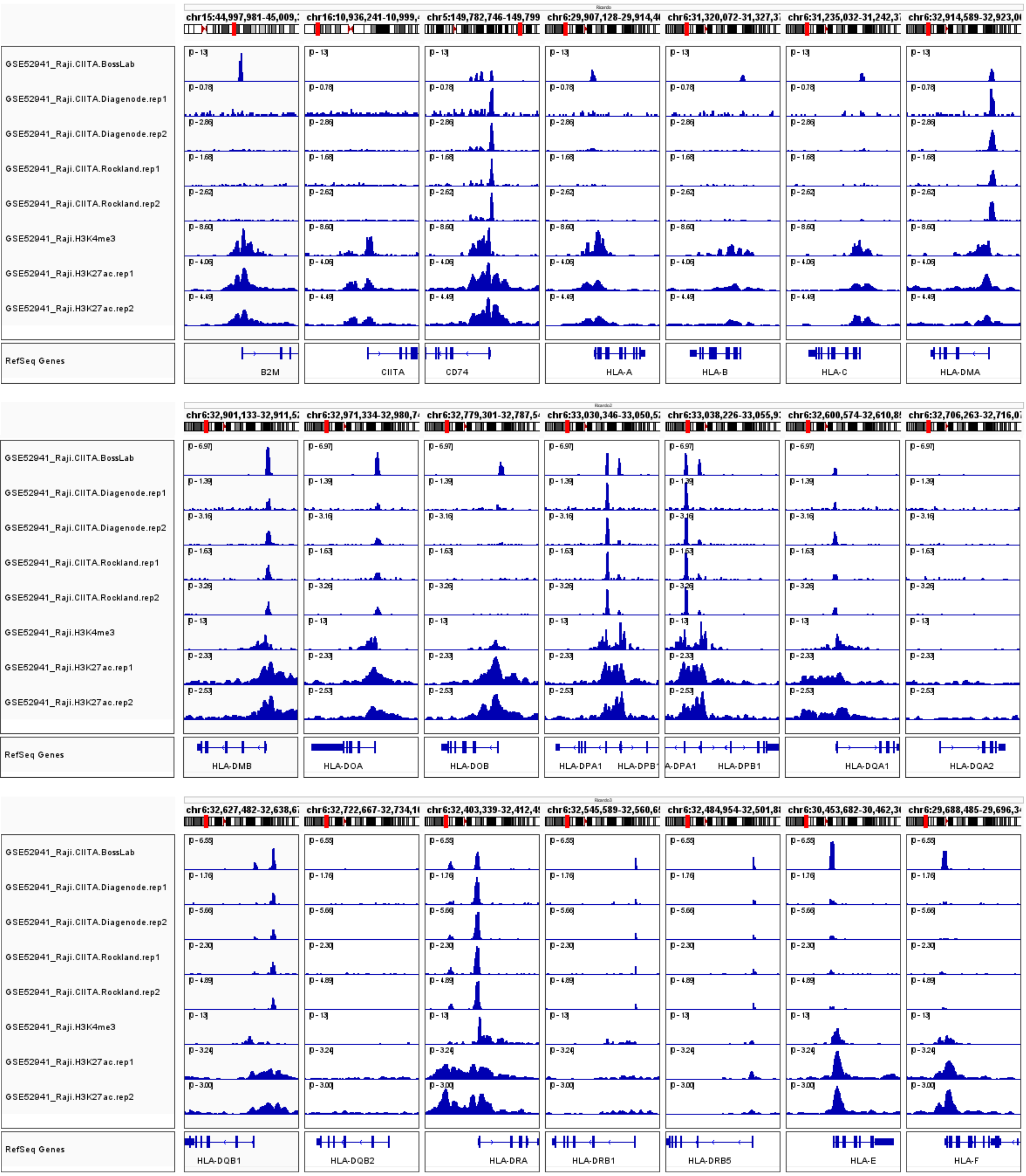

Figure S21 CIITA ChIP-seq data in B-cell lymphoma cell line Raji (GSE52941) – Data shown focus on the promoters of MHCI and MHCII associated genes. The top five tracks display pulldown with three distinct CIITA antibodies and their replicates. The bottom three tracks display histone (H3K4me3 and H3K27ac) modification in those promoter areas. Target promoters/genes are shown at the bottom, chromosomal locations at the top. Peaks for *CD74*, *HLA-DM*, *HLA-DB*, *HLA-DRA* (and *HLA-DPA1/B1*) are particularly well defined and reproducible with all antibodies, and chromatin modification indicative of active transcription.

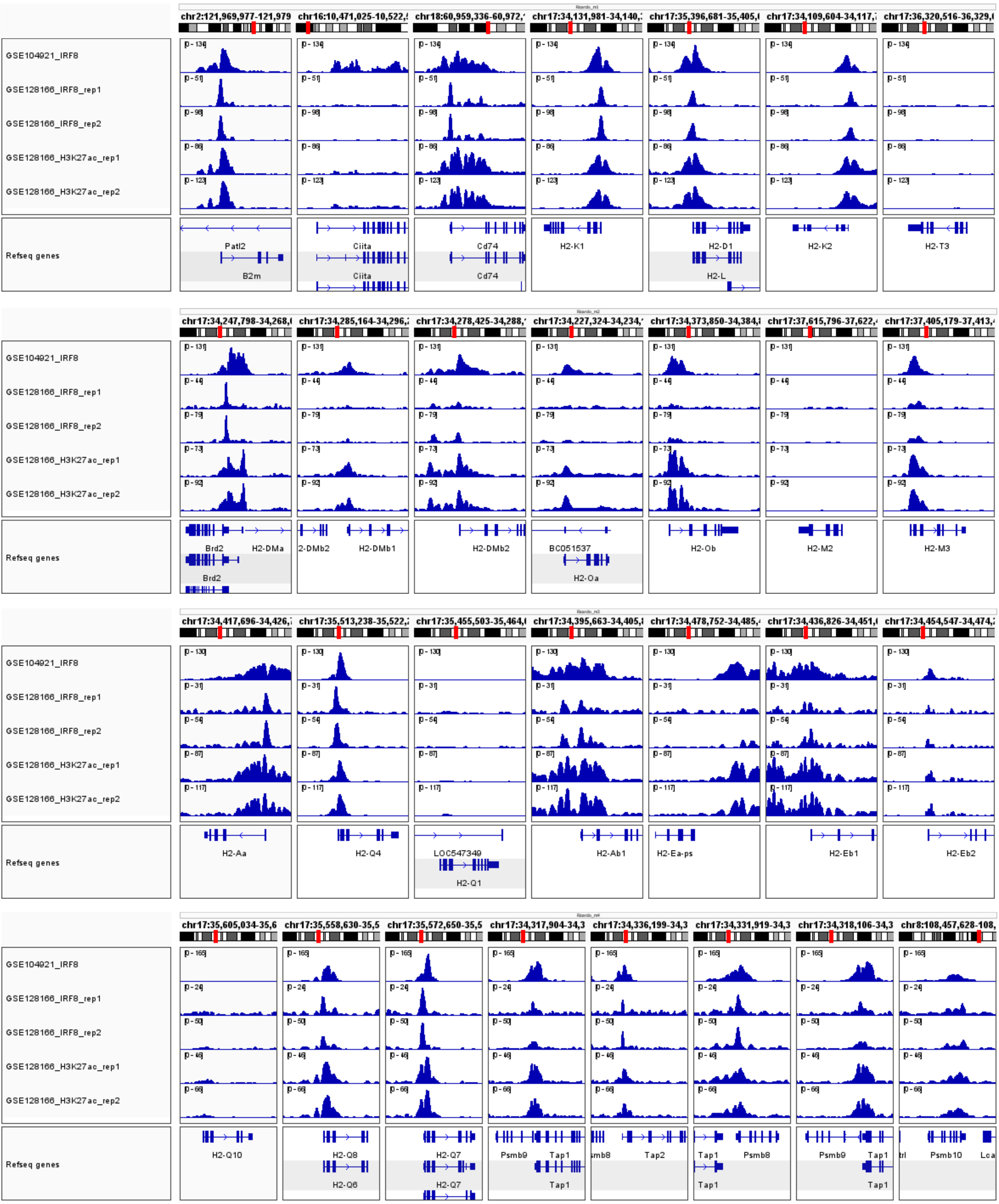

Figure S22. **IRF8 ChIP-seq data in murine mature B-cells (GSE104921, GSE128166) –** Data shown focus on the promoters of MHCI and MHCII associated genes. The top track displays pulldown of IRF8 in murine splenic B cells which were stimulated with F(ab') 2 fragment goat anti–mouse IgM, anti-CD40, IL-2, IL-4 and IL-5. The next four tracks display IRF8 and H3K27ac pulldowns (in replicates) in murine follicular naïve B cells (not activated). Target promoters/genes are shown at the bottom, chromosomal locations at the top. Peaks for *Cd74*, *H2-Dma*, *H2-Dmb2*, *HA-Aa* (as well as many others MHCI and MHCII relevant genes) are particularly well defined, whereas peaks in the *Ciita* promoter are restricted to activated B cells.

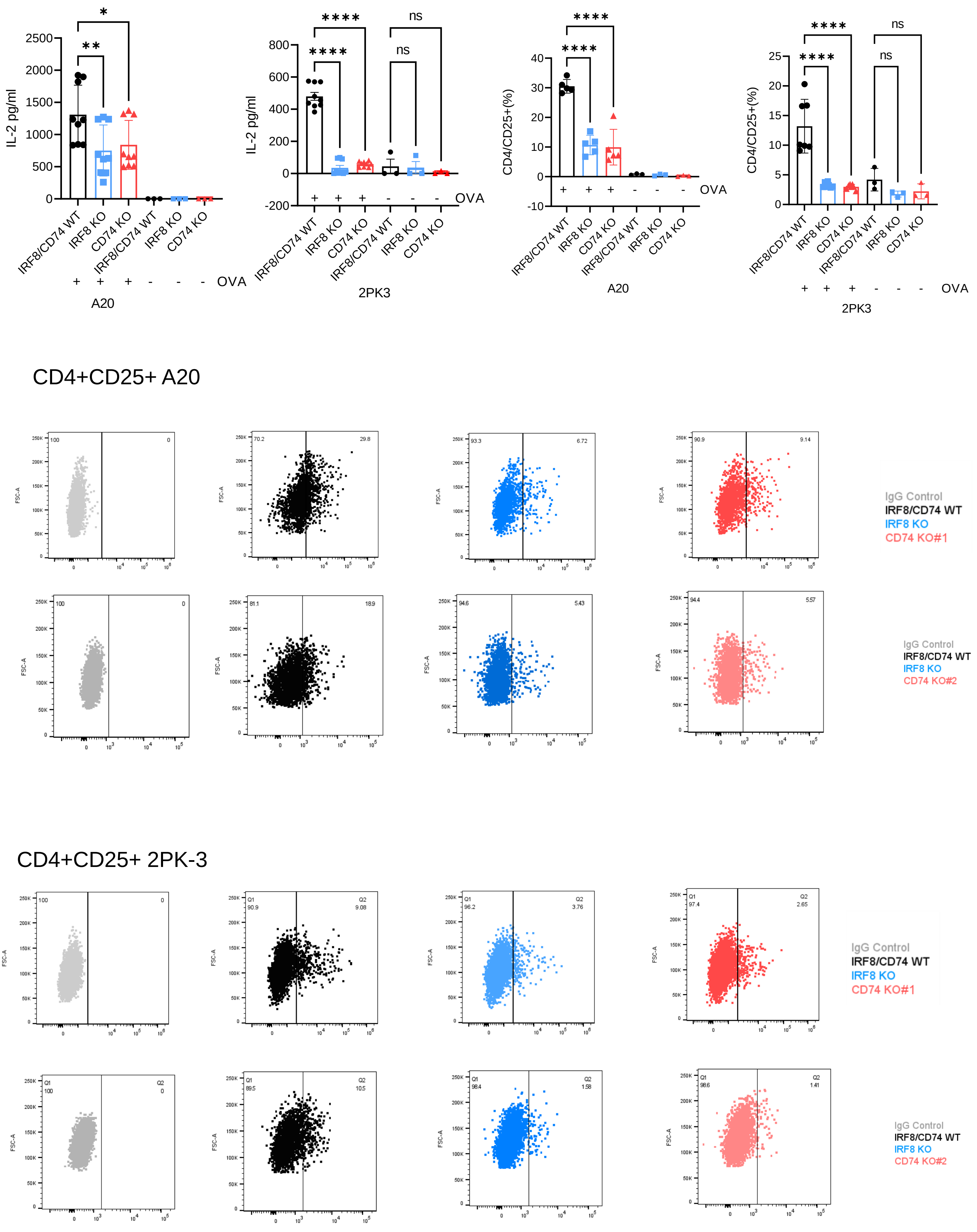

Figure S23. **Activation of CD4+ DO-11.10 cells. Top left panels**. IL-2 quantification in conditioned media of CD4+ DO-11.10 cells co-cultured with mouse B cell lymphoma cell lines A20 (left) or 2PK-3 (right), each expressing IRF8/CD74 WT or each individual KO, "loaded" or not with OVA. **Top right panels.** CD25 quantification by FACS in CD4+ DO-11.10 murine cells co-cultured with mouse B cell lymphoma cell lines A20 (left) or 2PK-3 (right), each expressing IRF8/CD74 WT or each individual KO, "loaded" or not with OVA. Data are mean ±SD of three biological replicates, performed with one, two or three technical replicates. P values are from one ANOVA with Bonferroni or Fisher's LSD post-test; *(p<0.05), **(p<0.01), ***(p<0.001), **** (p<0.0001). **Middle and Bottom panels.** Representative displays of CD25 quantification by FACS in CD4+ DO-11.10 murine cells following antigen (OVA) presentation by the B cell lymphoma cells A20 and 2PK-3, expressing IRF8/CD74 WT or each of the two CD74 KO (KO#1 or KO#2) polyclonal cell populations.

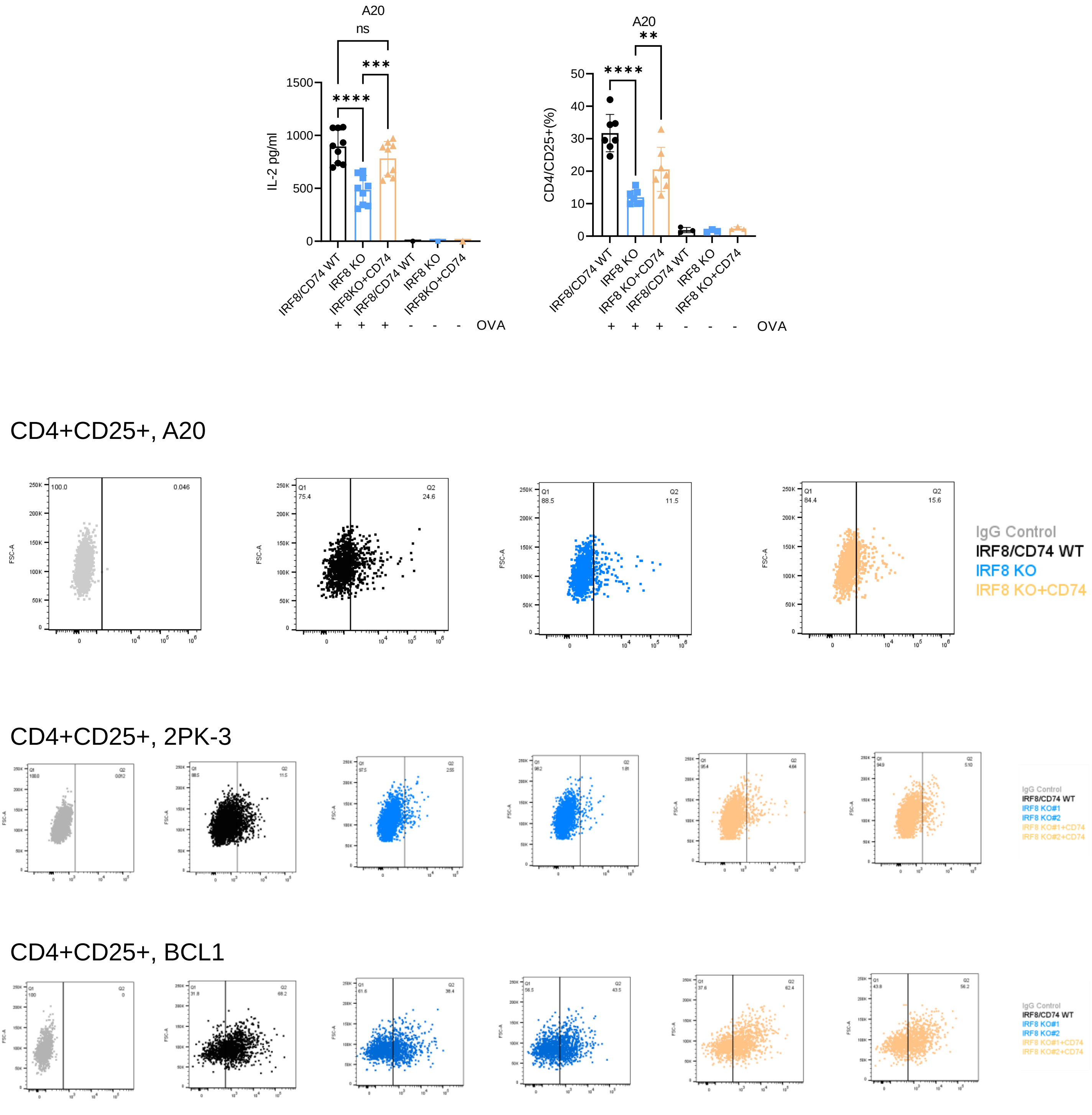

Figure S24. **Activation of CD4+ DO-11.10 cells. Top left bar-graph**. ELISA-based IL-2 quantification in conditioned media of CD4+ DO-11.10 cells co-cultured with the mouse B cell lymphoma cell line A20 expressing IRF8/CD74 WT, IRF8 KO, or IRF8 KO + ectopically expressed CD74, “loaded” or not with OVA. **Top right bar-graph.** CD25 quantification by FACS CD4+ DO-11.10 cells co-cultured with the mouse B cell lymphoma cell line A20 expressing IRF8/CD74 WT, IRF8 KO, or IRF8 KO + ectopically expressed CD74, “loaded” or not with OVA. Data are mean ±SD of three biological replicates, performed with one or three technical replicates. P values are from one-way ANOVA with Bonferroni post-test; **(p<0.01), ***(p<0.001), **** (p<0.0001). **Middle and bottom panels.** Representative displays of CD25 quantification by FACS in CD4+ DO-11.10 murine cells following antigen (OVA) presentation by the B cell lymphoma cell lines A20 , 2PK-3 and BCL1 expressing IRF8/CD74 WT, IRF8 KO or IRF8 KO + ectopically expressed CD74.

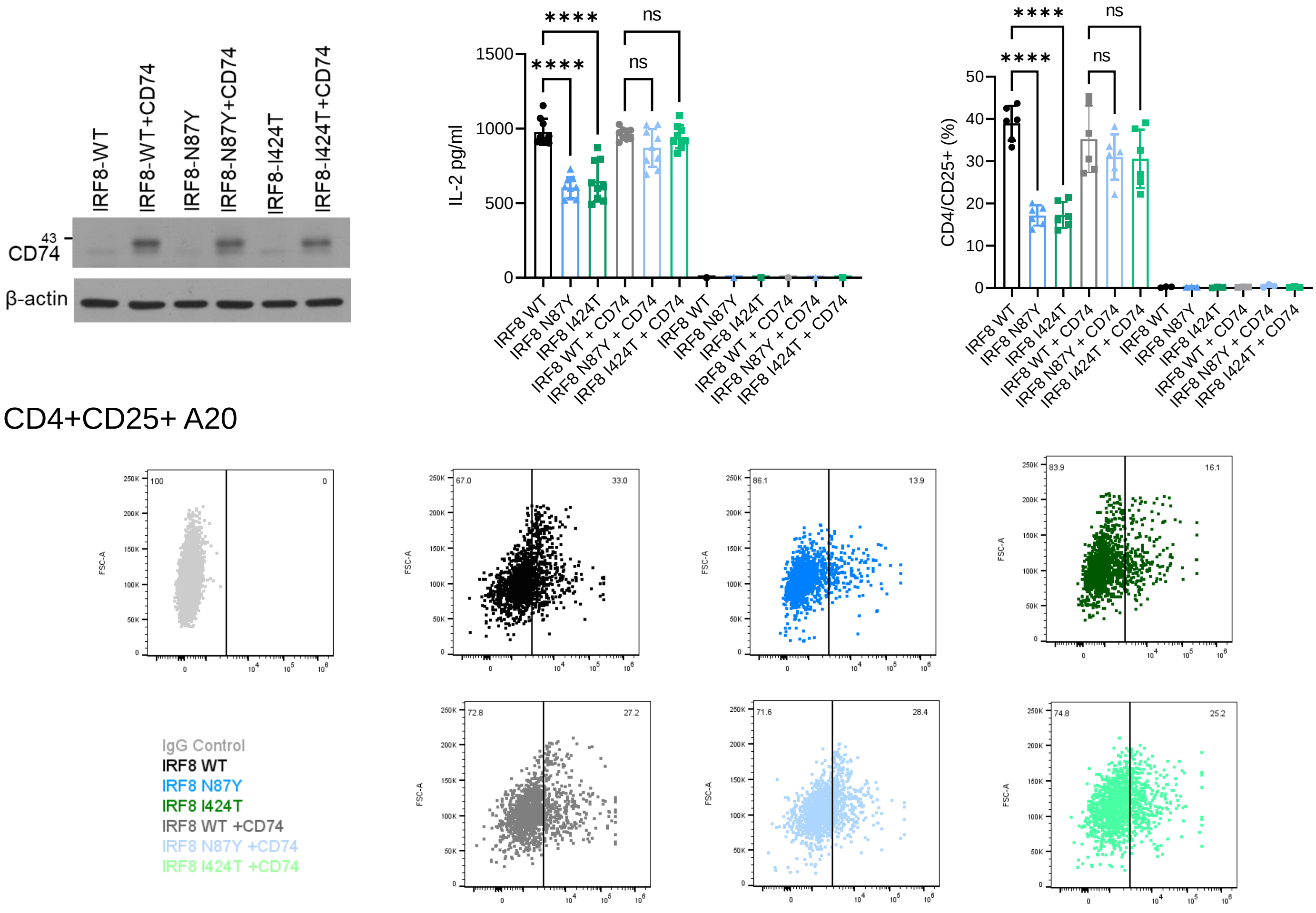

Figure S25. **Activation of CD4+ DO-11.10 cells. Top, left to right**. CD74 WB in A20 models expressing IRF8 WT, N87Y or I424T -/+ CD74. Bar-graphs; **left**, ELISA-based IL-2 quantification in conditioned media of CD4+ DO-11.10 cells co-cultured with the mouse B cell lymphoma cell line A20 expressing IRF8 WT, N87Y or I424T -/+ ectopically expressed CD74, "loaded" or not with OVA, **right,** CD25 quantification by FACS in CD4+ DO-11.10 cells co-cultured with the mouse B cell lymphoma cell line A20 expressing IRF8 WT, N87Y or I424T -/+ ectopically expressed CD74, "loaded" or not with OVA. Data are mean ±SD of three biological replicates, performed with one, two or three technical replicates. P values are from one-way ANOVA with Bonferroni post-test; **** ($p<0.0001$). **Bottom panels.** Representative displays of CD25 quantification by FACS in CD4+ DO-11.10 murine cells following antigen (OVA) presentation by the B cell lymphoma cell line A20 expressing IRF8 WT, N87Y or I424T -/+ ectopically expressed CD74.

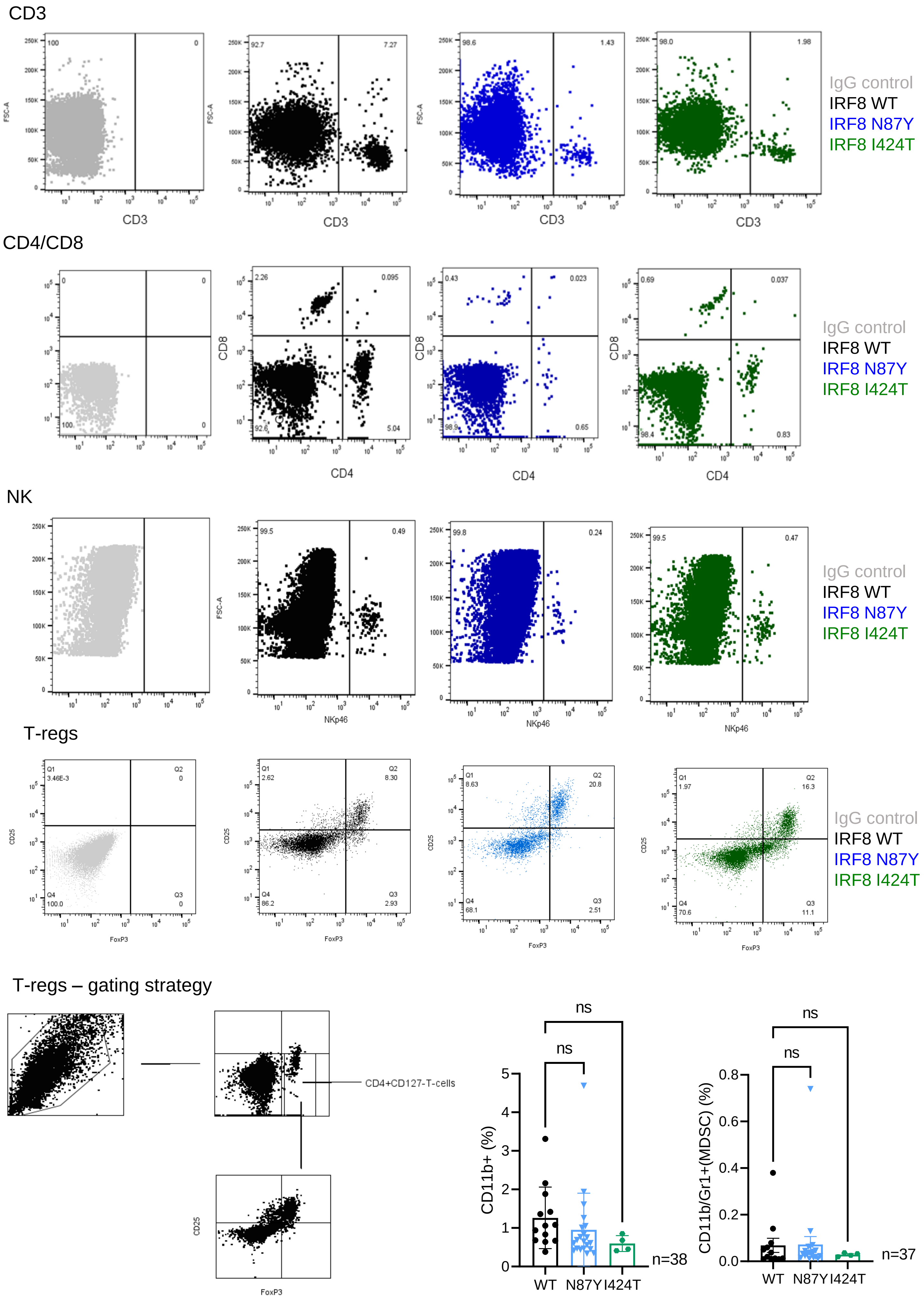

Figure S26. **FACS analysis of lymphoma microenvironment. Top to bottom.** Representative displays of CD3, CD4/CD8, NK, T-regs measurements in the TME of lymphomas expressing IRF8 WT, N87Y or I424T. Gating strategy for T-regs is also. Bar-graphs at the bottom right show the FACS-based quantification of monocytes and MDSC in the TME of lymphomas expressing IRF8 WT, N87Y or I424T. Data shown are mean ± SD of 38 and 37 mice, respectively. Statistical significance was tested with one-way ANOVA and Bonferroni post-test.

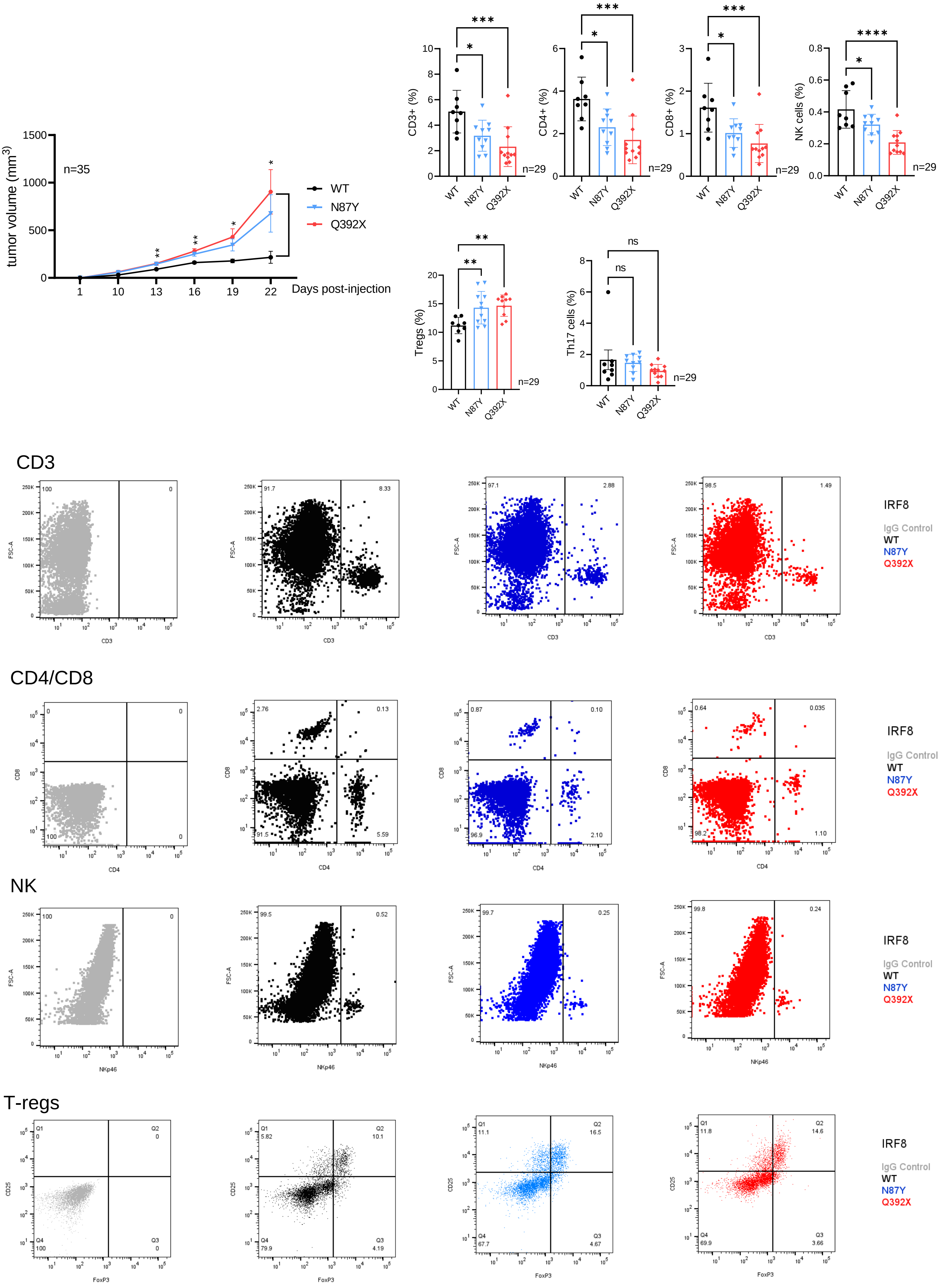

Figure S27. **Effect of IRF8 n-terminal missense versus c-terminal nonsense mutation on B cell lymphoma growth and immune microenvironment. Top left -** growth curve (volume) of lymphomas expressing IRF8 WT, N87Y or Q392X. Data are mean ± SEM; p values are from Mann-Whitney test. **Top right -** FACS-based quantification of CD3, CD4, CD8, NK, T-regs, and Th17 cells in the microenvironment of IRF8 WT or mutant lymphomas. Data shown are mean ± SD of 29 tumors (mice). Statistical significance was tested with one-way ANOVA and Dunnett post-test. **Bottom –** representative displays of CD3, CD4/CD8, NK, T-regs measurements in the TME of lymphomas expressing IRF8 WT, N87Y or Q392X mutants.

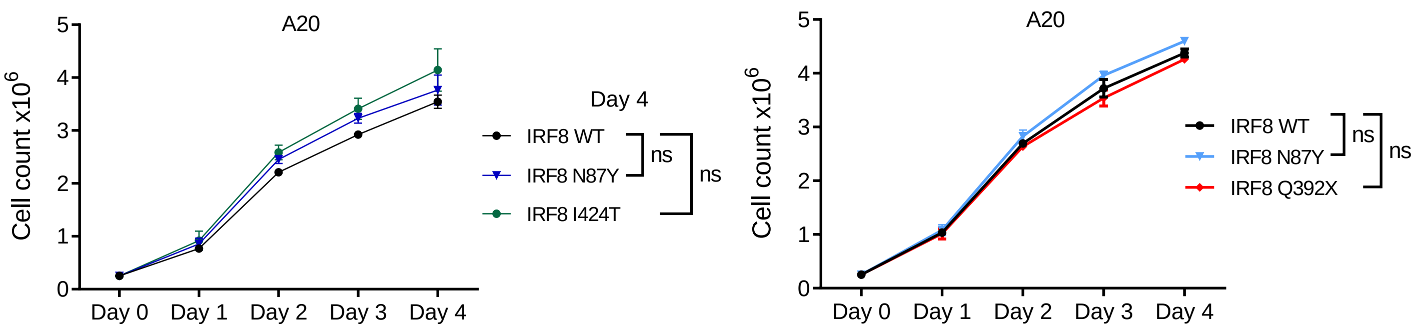

Figure S28. **In vitro growth curve of B cell lymphoma cell line A20** expressing IRF8 WT, N87Y or I424T mutants (left) or IR8 WT, N87Y or Q392X mutants (right), determined with automated fluorescent cell counter. Data are mean ± SD of three biological replicates. P value (non-significant, ns) is from two-sided Student's t-test, calculated in WT vs. each mutant, at the day 4 time point.

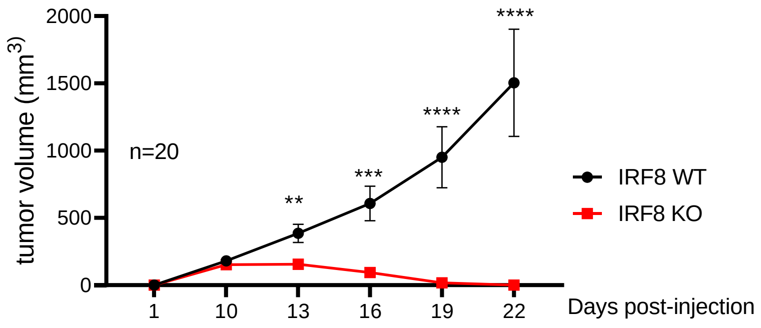

Figure S29. **In vivo growth curve (tumor volume)** of IRF8 WT or KO A20 lymphoma cells injected in BALB/c mice. Data are mean ± SEM of two independent cohorts; p values are from two- sided Student's t-test. **(p<0.01), ***(p<0.001), **** (p<0.0001).

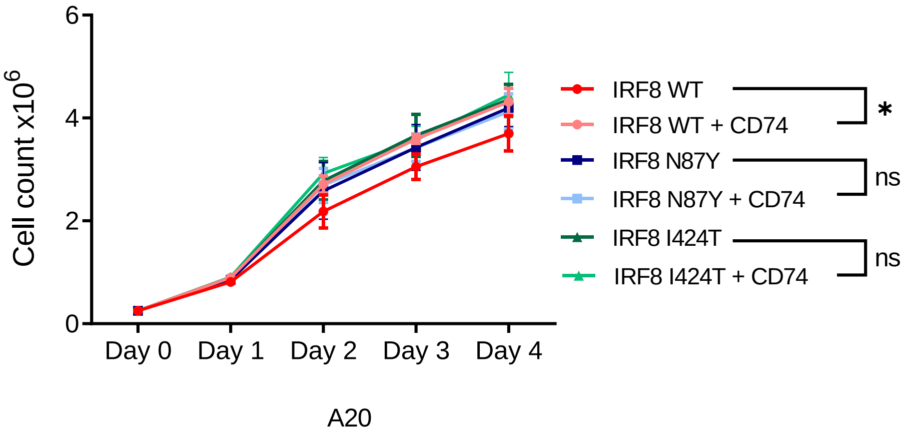

Figure S30. **In vitro growth curve of B cell lymphoma cell line A20** expressing IRF8 WT, N87Y or I424T mutants, -/+ CD74 stable ectopic expression, determined with automated fluorescent cell counter. Data are mean ± SD of three biological replicates. P values are from two-sided Student's t-test, calculated at the day 4 time point.

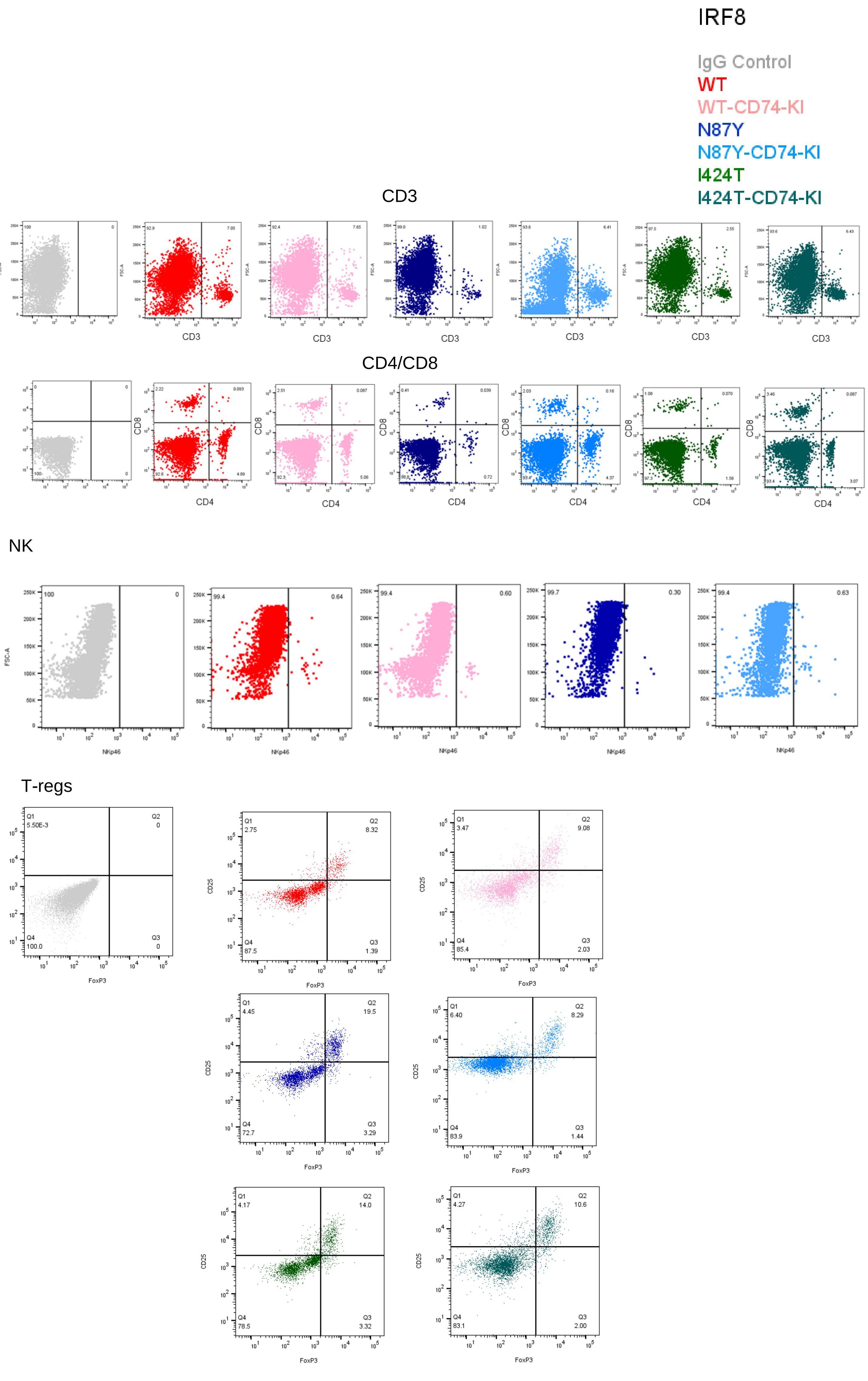

Figure S31. **FACS analysis of lymphoma microenvironment. Top to bottom.** Representative displays of CD3, CD4/CD8, NK, T-regs measurements in the TME of lymphomas expressing IRF8 WT, N87Y or I424T, -/+ CD74 ectopic expression. NK was quantified only in IRF8 WT and N87Y mutant. Gating strategy for T-regs was shown in Supplemental Figure 6A. Color labeling scheme is shown at the top right.

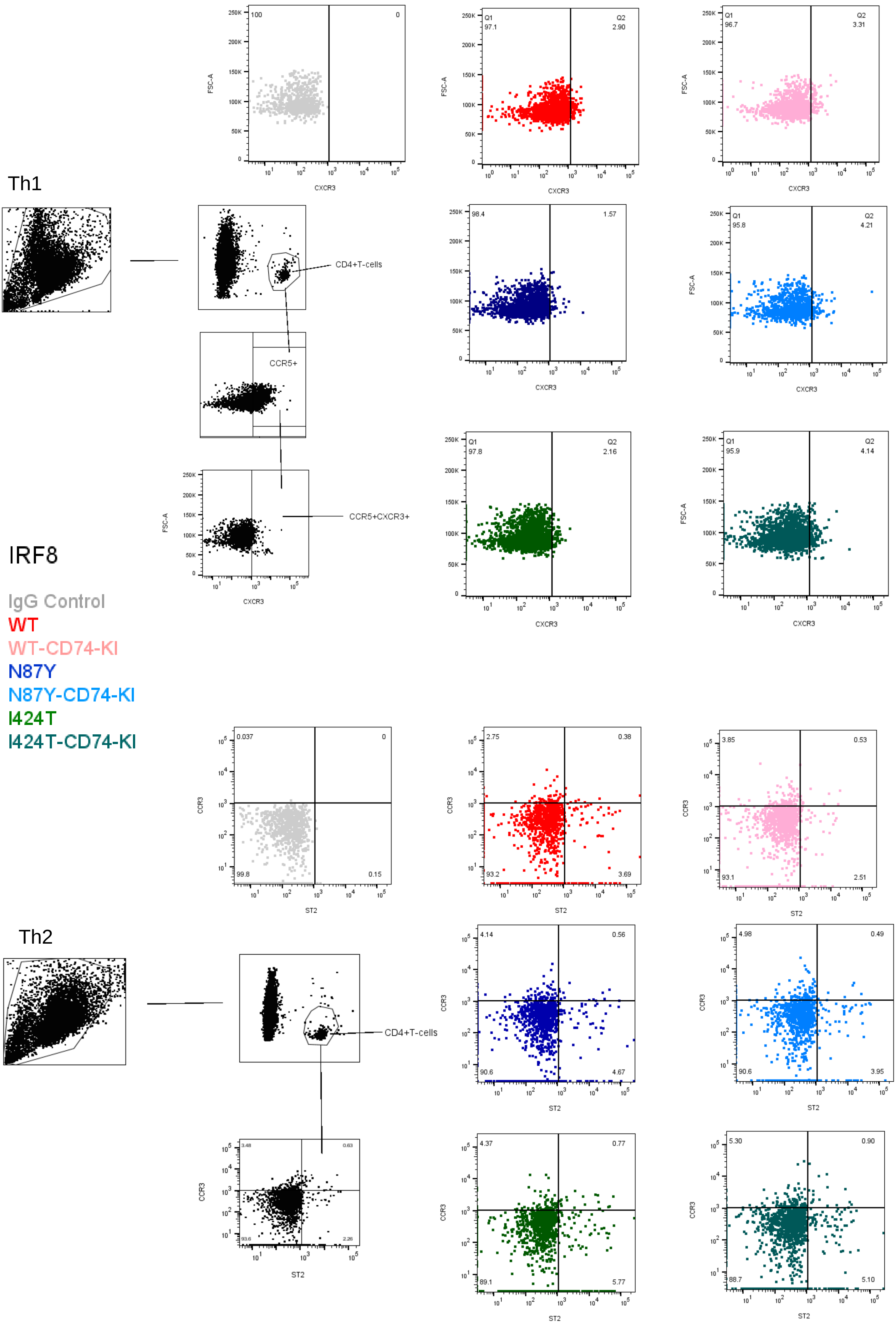

Figure S32. **FACS analysis of lymphoma microenvironment. Top to bottom.** Representative displays of Th1 and Th2 measurements in the TME of lymphomas expressing IRF8 WT, N87Y or I424T, -/+ CD74 ectopic expression. The gating strategies and color labeling scheme are shown to the left of the figure.

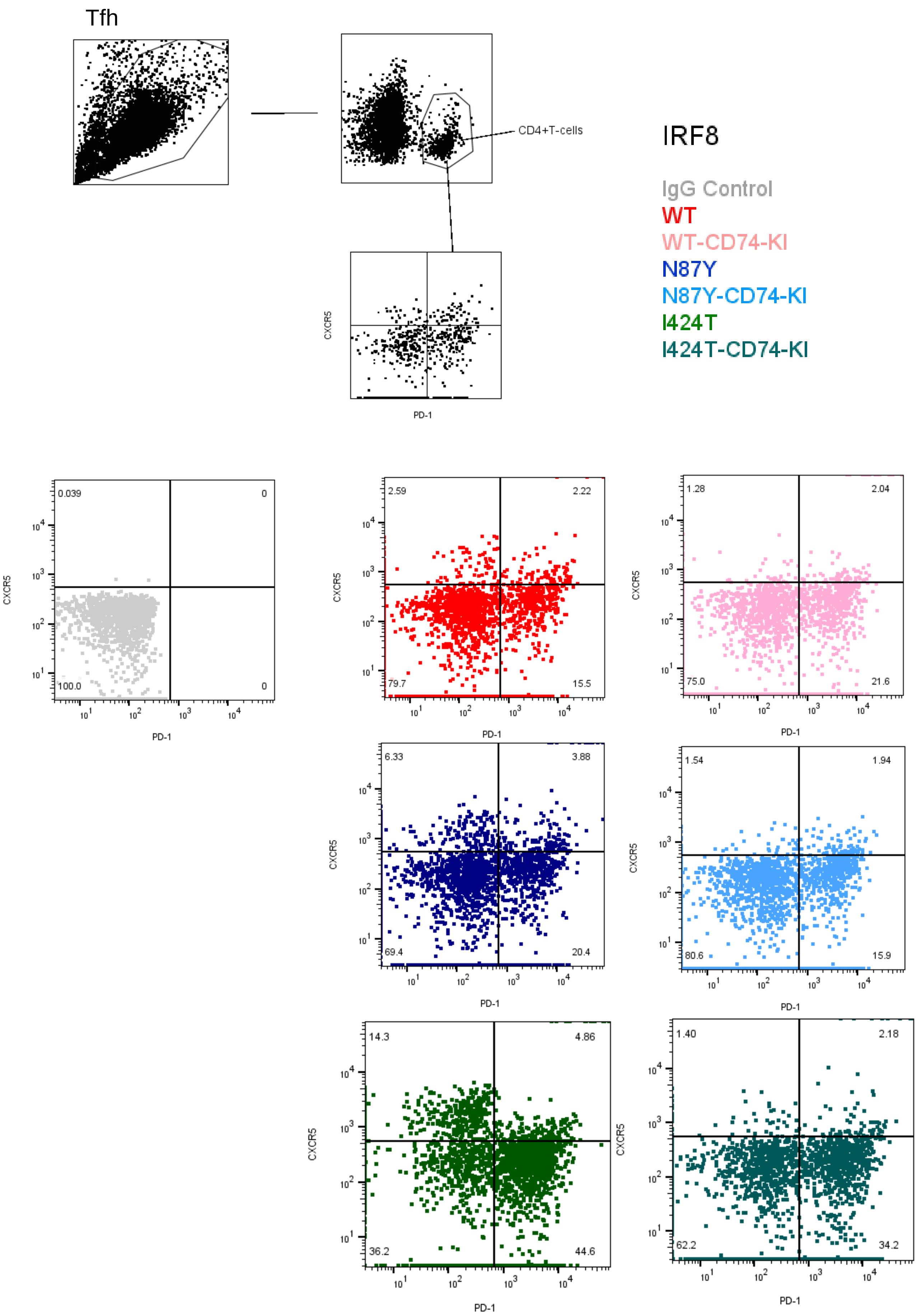

Figure S33. **FACS analysis of lymphoma microenvironment.** Representative displays of Tfh cell measurements in the TME of lymphomas expressing IRF8 WT, N87Y or I424T, -/+ CD74 ectopic expression. The gating strategies and color labeling scheme are shown at the top of the figure.

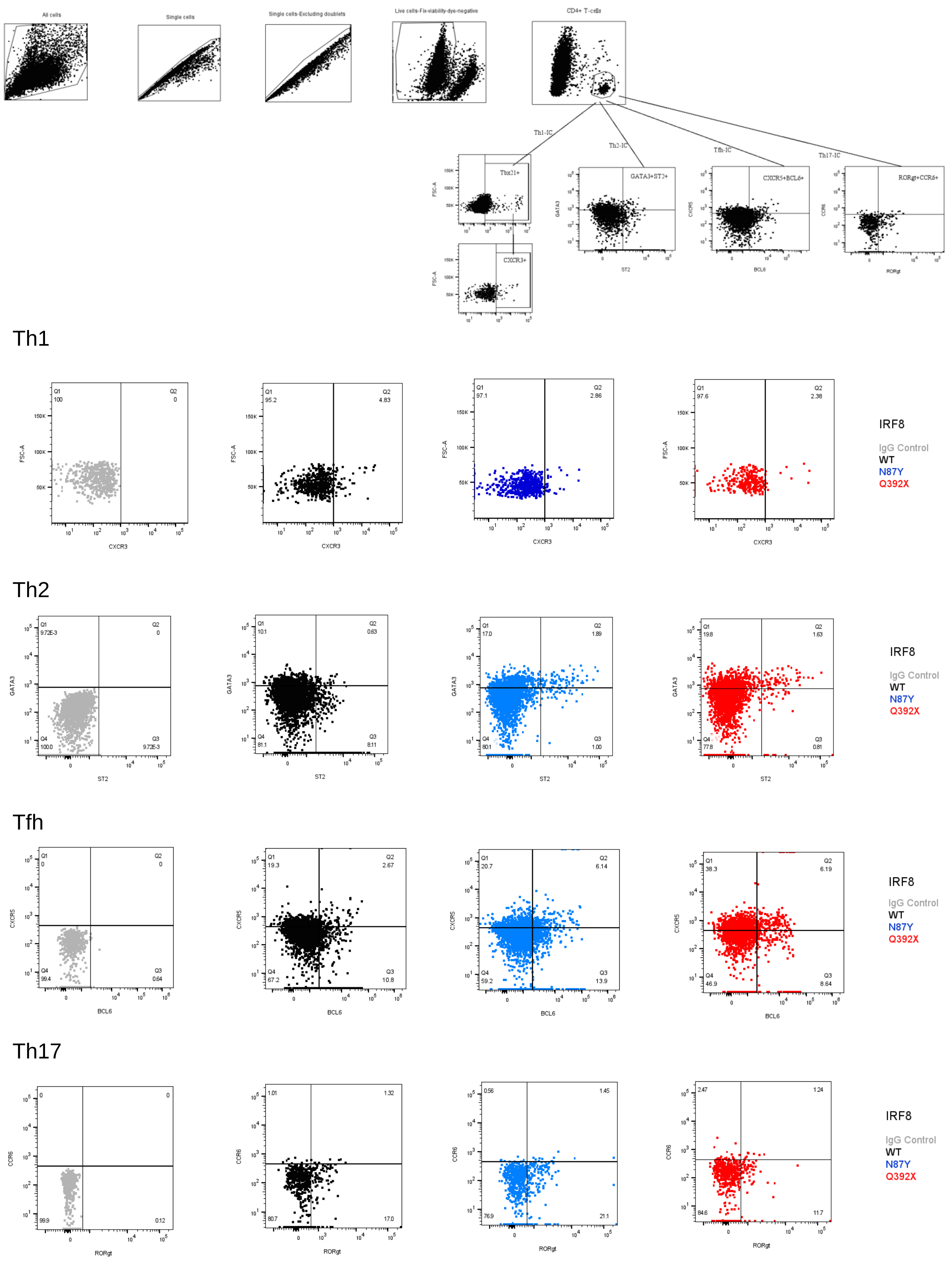

	Th1 cell surface	Th1 intra-cellular	Pearson *r*		Th2 cell surface	Th2 intra-cellular	Pearson *r*		Tfh cell surface	Tfh intra-cellular	Pearson *r*
IRF8 N87Y	0.95%	1.87%	0.986	IRF8 N87Y	0.54%	1.57%	0.982	IRF8 N87Y	2.32%	3.24%	0.969
IRF8 Q392X	0.72%	1.99%		IRF8 Q392X	0.34%	1.14%		IRF8 Q392X	1.95%	3.17%	
IRF8 WT	2.53%	3.89%		IRF8 WT	0.26%	0.78%		IRF8 WT	1.31%	2.70%	

Figure S34. **FACS analysis of lymphoma microenvironment.** Representative displays of CD4 subpopulations Th1, Th2, Tfh and Th17, detected with intra-cellular stain of specific transcription factors, in the TME of lymphomas expressing IRF8 WT, N87Y or Q39X mutants. The gating strategies and color labeling scheme are shown at the top of the figure. The high correlation (Pearson) between cell surface versus intra-cellular FACS for detection of Th1 (CCR5+CXCR3 vs. CXCR3+Tbx21), Th2 (CCR3+ST2 vs. ST2+GATA3) and Tfh (CXCR5+PD-1 vs. CXCR5+BCL6) is shown at the bottom.

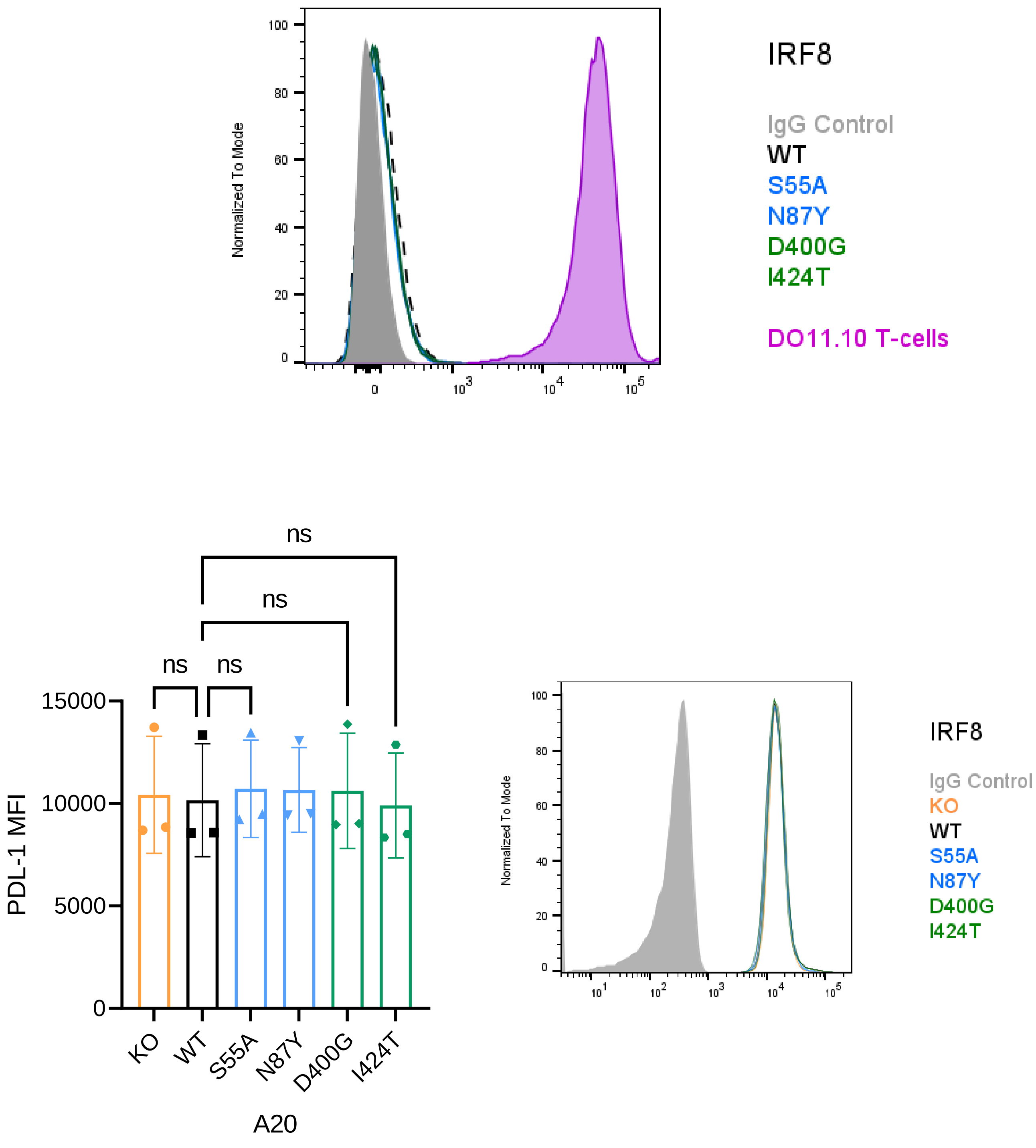

Figure S35. **FACS analysis of PD1 and PDL-1 in A20 lymphoma models of IRF8 WT or mutant. Top** - PD1 is not expressed in A20 cells, irrespective of IRF8 status – DO.11.10 CD4+ cells were used as positive controls. **Bottom** - PDL-1 expression (MFI left, three bio replicates) and representative histogram (right), indicate that IRF8 status does not influence PDL-1 levels in this model.

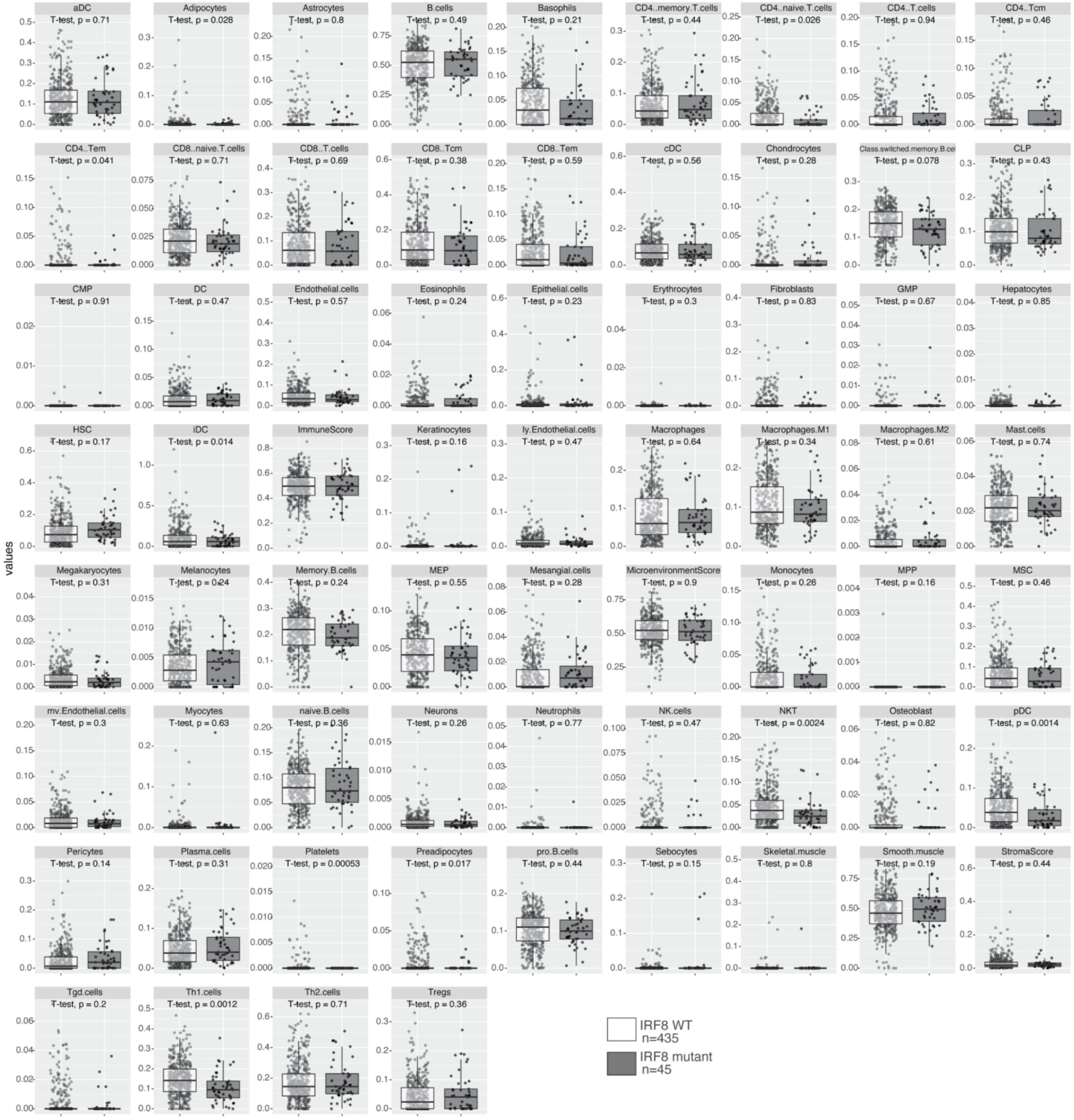

Figure S36. Immune composition of the lymphoma microenvironment of IRF8-WT and mutant human primary DLBCLs. Estimates of 64 cell type distributions scores from 480 DLBCL RNAseq (from dbGAPphs001444.v2.p1) using xCELL in IRF8 mutant (gray, n=45) or wildtype (WT, white, n=435) tumors, boxplots depict median and interquartile range, p-values are from two-sided Student's t-tests. Related to Figure 7A.

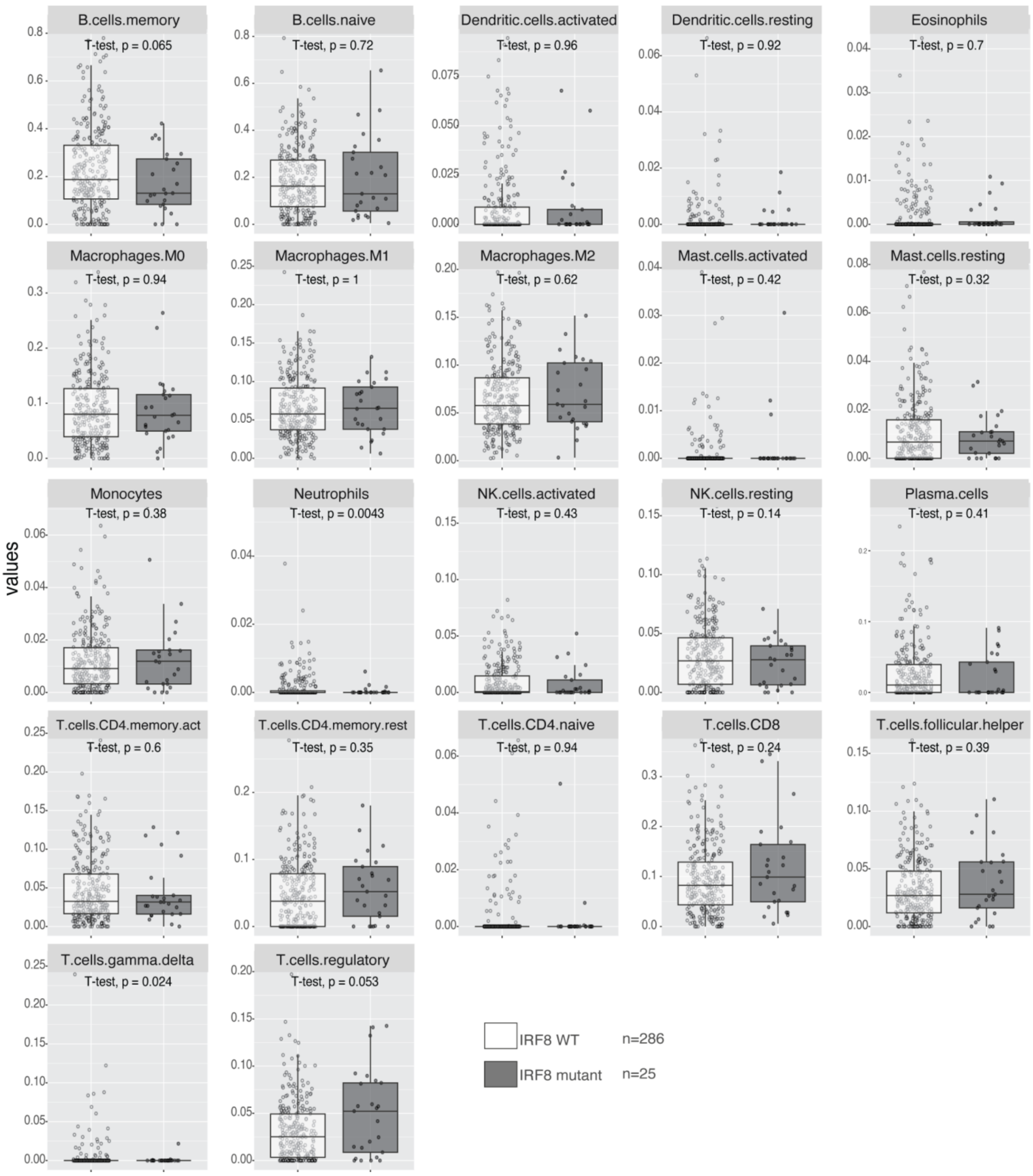

Figure S37. Immune composition of the lymphoma microenvironment of IRF8-WT and mutant human primary DLBCLs. CIBERSTORx-estimation of proportions of 22 immune cell types in a DLBLC sub-cohort (IRF8 WT, n = 286, IRF8 mutant, n = 25), that excludes tumors with mutation in other genes known to deregulate antigen processing/presentation (CIITA, B2M, CREBBP, EP300 and EZH2). Boxplots depict median and IQR. P values are from two-sided Student’s t-tests. Related to Figure 7B.

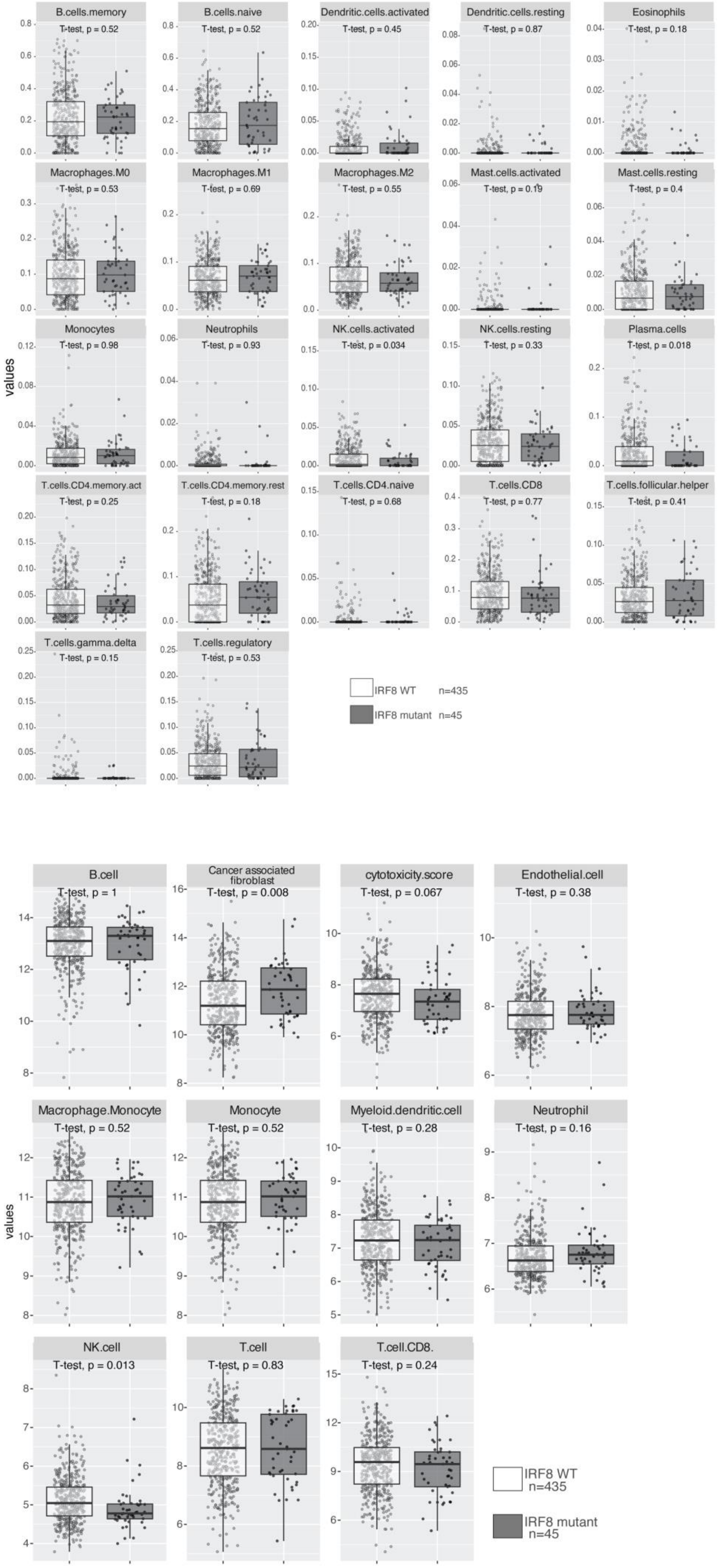

Figure S38. Immune composition of the lymphoma microenvironment of IRF8-WT and mutant human primary DLBCLs. **Top.** CIBERSTORx-estimation of proportions of 22 immune cell types in IRF8 mutant (gray, n=45) or wildtype (WT, white, n=435) DLBCL RNAseq (from dbGAPphs001444.v2.p1). Boxplots depict median and IQR. P values are from two-sided Students' t-test. **Bottom.** Estimates of 11 immune cell types or scores distributions from 480 DLBCL RNAseq (from dbGAPphs001444.v2.p1) using MCP-counter in IRF8 mutant (gray, n=45) or wildtype (WT, white, n=435) tumors. Boxplots depict median and interquartile range, p-values are two-sided Student's t-tests. Related to Figure 7C.

Contents

Table S1. Summary of publicly available cohorts of Diffuse Large B-Cell Lymphomas (DLBCLs) reporting*IRF8* mutations
Table S2. IRF8 mutations reported in Diffuse Large B-Cell Lymphomas (DLBCLs)
Table S3. IRF8 variant allele frequency (VAF) % in DLBCLs
Table S4. Cell of origin (COO) and subgroup classification of Diffuse Large B-Cell Lymphomas (DLBCLs) carrying IRF8 mutation from publicly available cohort
Table S5. Summary of IRF8 mutation rates in published cohorts of other mature B-cell malignancies
Table S6. List of IRF8 mutations in published cohorts of other mature B-cell malignancies
Table S7. IRF8 mutations reported in Diffuse Large B-Cell Lymphomas (DLBCLs) from NCI cohort, reanalyzed from dbGAPphs001444.v2.p1
Table S8. Oligonucleotide sequences

Table S1. Summary of publicly available cohorts of Diffuse Large B-Cell Lymphomas (DLBCLs) reporting *IRF8* mutations

**Study Name**	**DLBCLs (n)**	**IRF8 mutant tumors (n)**	**% samples with IRF8 mutation**	**Source**
Diffuse Large B cell Lymphoma (DFCI)	135	9	6.7	cBioportal and *(12)*
Diffuse Large B-cell Lymphoma (BCGSC)	30	3	10.0	cBioportal and *(39)*
Diffuse Large B-Cell Lymphoma (TCGA, PanCancer Atlas)	41	4	9.8	cBioportal
Diffuse Large B-Cell Lymphoma (Duke)	1001	64	6.4	cBioportal and *(37)*
Mature B-cell malignancies (MD Anderson Cancer Center)	148	8	5.4	cBioPortal and *(40)*
Diffuse Large B-Cell Lymphoma (NCI, dbGAP)	574	49	8.5	dbGAP and *(13)*
Diffuse Large B-Cell Lymphoma (UK)	929	73	7.9	*(36)*
Diffuse Large B-Cell Lymphoma (LymphGen and BCGSC)	329	34	10.3	*(7, 38, 41)*
**Total**	**3187**	**244**	**8.1**	

Table S2. IRF8 mutations reported in Diffuse Large B-Cell Lymphomas (DLBCLs)

Study of Origin	Sample ID	IRF8 Protein Change	IRF8 Domain	Mutation Type
(12)	DLBCL-RICOVER_1106	T80A	DNA-binding	Missense_Mutation
(12)	DLBCL-RICOVER_1199	P398Rfs*56	C-terminus	Frame_Shift_Ins
(12)	DLBCL-LS3615	I424N	C-terminus	Missense_Mutation
(12)	DLBCL-RICOVER_950	Y23H	DNA-binding	Missense_Mutation
(12)	DLBCL-LS3309	P398Rfs*56	C-terminus	Frame_Shift_Ins
(12)	DLBCL-RICOVER_267	G388Afs*3	C-terminus	Frame_Shift_Del
(12)	DLBCL-LS4618	S55A	DNA-binding	Missense_Mutation
(12)	DLBCL-RICOVER_197	S55A	DNA-binding	Missense_Mutation
(12)	DLBCL-LS146	N131D	DNA-binding	Missense_Mutation
(39)	RG116	S55A	DNA-binding	Missense_Mutation
(39)	RG064	S55A	DNA-binding	Missense_Mutation
(39)	RG083	S34T	DNA-binding	Missense_Mutation
(37)	695	N87Y	DNA-binding	Missense_Mutation
(37)	2075	Y23H	DNA-binding	Missense_Mutation
(37)	2076	V287M	IRF-association	Missense_Mutation
(37)	2135	P237S	IRF-association	Missense_Mutation
(37)	2154	D256N	IRF-association	Missense_Mutation
(37)	2190	S416*	C-terminus	Nonsense_Mutation
(37)	2209	I424T	C-terminus	Missense_Mutation
(37)	2251	A257T	IRF-association	Missense_Mutation
(37)	2262	A61T	DNA-binding	Missense_Mutation
(37)	2297	E314K	IRF-association	Missense_Mutation
(37)	2459	*427Eext*24	C-terminus	Nonstop_Mutation
(37)	2471	Q118K	DNA-binding	Missense_Mutation
(37)	2522	Q392*	C-terminus	Nonsense_Mutation
(37)	2530	H413Tfs*57	C-terminus	Frame_Shift_Ins
(37)	2533	E115K	DNA-binding	Missense_Mutation
(37)	2599	Y23H	DNA-binding	Missense_Mutation
(37)	2640	I424Dfs*46	C-terminus	Frame_Shift_Ins
(37)	2680	Q180P	IRF-association	Missense_Mutation
(37)	2696	V287M	IRF-association	Missense_Mutation
(37)	2728	T96M	DNA-binding	Missense_Mutation
(37)	2779	K66R	DNA-binding	Missense_Mutation
(37)	2779	E93G	DNA-binding	Missense_Mutation
(37)	2805	R268W	IRF-association	Missense_Mutation
(37)	2813	A257T	IRF-association	Missense_Mutation
(37)	2837	K66R	DNA-binding	Missense_Mutation
(37)	2871	T80A	DNA-binding	Missense_Mutation
(37)	2897	L86F	DNA-binding	Missense_Mutation
(37)	2900	R268W	IRF-association	Missense_Mutation
(37)	2918	D256N	IRF-association	Missense_Mutation
(37)	2918	E353K	IRF-association	Missense_Mutation
(37)	2934	Q392*	C-terminus	Nonsense_Mutation
(37)	2944	C84W	DNA-binding	Missense_Mutation
(37)	2945	R161dup	IRF-association	In_Frame_Ins
(37)	2950	T80A	DNA-binding	Missense_Mutation
(37)	3386	E115K	DNA-binding	Missense_Mutation
(37)	3417	Q422delinsPTDHRLK	C-terminus	In_Frame_Ins
(37)	3460	T80A	DNA-binding	Missense_Mutation
(37)	3485	G238S	IRF-association	Missense_Mutation
(37)	3535	Y23H	DNA-binding	Missense_Mutation
(37)	3542	Y23H	DNA-binding	Missense_Mutation
(37)	3632	C410*	C-terminus	Nonsense_Mutation
(37)	3635	Y23H	DNA-binding	Missense_Mutation
(37)	3647	K66R	DNA-binding	Missense_Mutation
(37)	3647	T96M	DNA-binding	Missense_Mutation
(37)	3697	T80A	DNA-binding	Missense_Mutation
(37)	3737	T80A	DNA-binding	Missense_Mutation
(37)	3740	T96M	DNA-binding	Missense_Mutation
(37)	3745	G301D	IRF-association	Missense_Mutation
(37)	3750	S416*	C-terminus	Nonsense_Mutation
(37)	3758	C410*	C-terminus	Nonsense_Mutation
(37)	3760	Q423*	C-terminus	Nonsense_Mutation
(37)	3812	A76V	DNA-binding	Missense_Mutation
(37)	3821	V287M	IRF-association	Missense_Mutation
(37)	3835	Y23H	DNA-binding	Missense_Mutation
(37)	3840	N87Y	DNA-binding	Missense_Mutation
(37)	3851	*427Eext*24	C-terminus	Nonstop_Mutation
(37)	3856	N87Y	DNA-binding	Missense_Mutation
(37)	3856	I112F	DNA-binding	Missense_Mutation
(37)	3892	A257T	IRF-association	Missense_Mutation
(37)	3900	G388D	C-terminus	Missense_Mutation
(37)	3901	C84R	DNA-binding	Missense_Mutation
(37)	3909	D256N	IRF-association	Missense_Mutation
(37)	3924	Q401Tfs*53	C-terminus	Frame_Shift_Ins
(37)	3936	K66R	DNA-binding	Missense_Mutation
(37)	3943	G388D	C-terminus	Missense_Mutation
(37)	3967	T80A	DNA-binding	Missense_Mutation
(37)	3969	A257T	IRF-association	Missense_Mutation
(37)	3972	Q422Tfs*38	C-terminus	Frame_Shift_Ins
Diffuse Large B-Cell Lymphoma (TCGA, PanCancer Atlas)	TCGA-RQ-A6JB-01	S55A	DNA-binding	Missense_Mutation
Diffuse Large B-Cell Lymphoma (TCGA, PanCancer Atlas)	TCGA-GS-A9TZ-01	D91N	DNA-binding	Missense_Mutation
Diffuse Large B-Cell Lymphoma (TCGA, PanCancer Atlas)	TCGA-GS-A9TX-01	E395K	C-terminus	Missense_Mutation
Diffuse Large B-Cell Lymphoma (TCGA, PanCancer Atlas)	TCGA-GS-A9TY-01	T80A	DNA-binding	Missense_Mutation
(40)	DLBCL_009	I424F	C-terminus	Missense_Mutation
(40)	DLBCL_012	Q392*	C-terminus	Nonsense_Mutation
(40)	DLBCL_019	Y23H	DNA-binding	Missense_Mutation
(40)	DLBCL_019	S20R	DNA-binding	Missense_Mutation
(40)	DLBCL_031	A76V	DNA-binding	Missense_Mutation
(40)	DLBCL_038	G7del	DNA-binding	In_Frame_Del
(40)	DLBCL_057	D91N	DNA-binding	Missense_Mutation
(40)	DLBCL_058	S55A	DNA-binding	Missense_Mutation
(40)	DLBCL_060	Y23H	DNA-binding	Missense_Mutation
(40)	DLBCL_120	S55A	DNA-binding	Missense_Mutation
(40)	DLBCL_124	T80A	DNA-binding	Missense_Mutation
(40)	DLBCL_135	E380Rfs*11	IRF-association	Frame_Shift_Del
(40)	DLBCL_144	K66R	DNA-binding	Missense_Mutation
(40)	DLBCL_144	L82F	DNA-binding	Missense_Mutation
(40)	DLBCL_144	I424S	C-terminus	Missense_Mutation
*(7, 38, 41) **	DLC0041	N/A	na	Truncation_Mutation
*(7, 38, 41) **	DLC0047	N/A	na	Missense_Mutation
*(7, 38, 41) **	DLC0087	N/A	na	Missense_Mutation
*(7, 38, 41) **	DLC0097	N/A	na	Missense_Mutation
*(7, 38, 41) **	DLC0105	N/A	na	Missense_Mutation
*(7, 38, 41) **	DLC0105	N/A	na	Truncation_Mutation
*(7, 38, 41) **	DLC0123	N/A	na	Missense_Mutation
*(7, 38, 41) **	DLC0134	N/A	na	Missense_Mutation
*(7, 38, 41) **	DLC0155	N/A	na	Truncation_Mutation
*(7, 38, 41) **	DLC0161	N/A	na	Missense_Mutation
*(7, 38, 41) **	DLC0169	N/A	na	Missense_Mutation
*(7, 38, 41) **	DLC0183	N/A	na	Missense_Mutation
*(7, 38, 41) **	DLC0194	N/A	na	Missense_Mutation
*(7, 38, 41) **	DLC0210	N/A	na	Missense_Mutation
*(7, 38, 41) **	DLC0236	N/A	na	Truncation_Mutation
*(7, 38, 41) **	DLC0246	N/A	na	Missense_Mutation
*(7, 38, 41) **	DLC0246	N/A	na	Truncation_Mutation
*(7, 38, 41) **	DLC0252	N/A	na	Truncation_Mutation
*(7, 38, 41) **	DLC0255	N/A	na	Missense_Mutation
*(7, 38, 41) **	DLC0256	N/A	na	Missense_Mutation
*(7, 38, 41) **	DLC0267	N/A	na	Truncation_Mutation
*(7, 38, 41) **	DLC0269	N/A	na	Missense_Mutation
*(7, 38, 41) **	DLC0270	N/A	na	Missense_Mutation
*(7, 38, 41) **	DLC0280	N/A	na	Missense_Mutation
*(7, 38, 41) **	DLC0310	N/A	na	Truncation_Mutation
*(7, 38, 41) **	DLC0317	N/A	na	Truncation_Mutation
*(7, 38, 41) **	DLC0325	N/A	na	Missense_Mutation
*(7, 38, 41) **	DLC0326	N/A	na	Missense_Mutation
*(7, 38, 41) **	DLC0353	N/A	na	Truncation_Mutation
*(7, 38, 41) **	DLC0370	N/A	na	Missense_Mutation
*(7, 38, 41) **	DLC0371	N/A	na	Truncation_Mutation
*(7, 38, 41) **	DLC0374	N/A	na	Missense_Mutation
*(7, 38, 41) **	DLC0380	N/A	na	Truncation_Mutation
*(7, 38, 41) **	DLC0382	N/A	na	Missense_Mutation
*(7, 38, 41) **	DLC0393	N/A	na	Truncation_Mutation
*(7, 38, 41) **	DLC0397	N/A	na	Missense_Mutation
Diffuse Large B cell Lymphoma (NCI Cohort, dbGAPphs001444.v2.p1)**	N/A	A76V	DNA-binding	Missense_Mutation
Diffuse Large B cell Lymphoma (NCI Cohort, dbGAPphs001444.v2.p1)**	N/A	Q392X	C-terminus	Truncation_Mutation
Diffuse Large B cell Lymphoma (NCI Cohort, dbGAPphs001444.v2.p1)**	N/A	D91A	DNA-binding	Missense_Mutation
Diffuse Large B cell Lymphoma (NCI Cohort, dbGAPphs001444.v2.p1)**	N/A	P114R	DNA-binding	Missense_Mutation
Diffuse Large B cell Lymphoma (NCI Cohort, dbGAPphs001444.v2.p1)**	N/A	L82V	DNA-binding	Missense_Mutation
Diffuse Large B cell Lymphoma (NCI Cohort, dbGAPphs001444.v2.p1)**	N/A	F417V	C-terminus	Missense_Mutation
Diffuse Large B cell Lymphoma (NCI Cohort, dbGAPphs001444.v2.p1)**	N/A	Y23H	DNA-binding	Missense_Mutation
Diffuse Large B cell Lymphoma (NCI Cohort, dbGAPphs001444.v2.p1)**	N/A	R276H	IRF-association	Missense_Mutation
Diffuse Large B cell Lymphoma (NCI Cohort, dbGAPphs001444.v2.p1)**	N/A	X427fs	C-terminus	Truncation_Mutation
Diffuse Large B cell Lymphoma (NCI Cohort, dbGAPphs001444.v2.p1)**	N/A	X427QextX?	C-terminus	Missense_Mutation
Diffuse Large B cell Lymphoma (NCI Cohort, dbGAPphs001444.v2.p1)**	N/A	R419fs	C-terminus	Truncation_Mutation
Diffuse Large B cell Lymphoma (NCI Cohort, dbGAPphs001444.v2.p1)**	N/A	V426fs	C-terminus	Truncation_Mutation
Diffuse Large B cell Lymphoma (NCI Cohort, dbGAPphs001444.v2.p1)**	N/A	X427fs	C-terminus	Truncation_Mutation
Diffuse Large B cell Lymphoma (NCI Cohort, dbGAPphs001444.v2.p1)**	N/A	R415fs	C-terminus	Truncation_Mutation
Diffuse Large B cell Lymphoma (NCI Cohort, dbGAPphs001444.v2.p1)**	N/A	S416fs	C-terminus	Truncation_Mutation
Diffuse Large B cell Lymphoma (NCI Cohort, dbGAPphs001444.v2.p1)**	N/A	V426fs	C-terminus	Truncation_Mutation
Diffuse Large B cell Lymphoma (NCI Cohort, dbGAPphs001444.v2.p1)**	N/A	D91N	DNA-binding	Missense_Mutation
Diffuse Large B cell Lymphoma (NCI Cohort, dbGAPphs001444.v2.p1)**	N/A	K45R	DNA-binding	Missense_Mutation
Diffuse Large B cell Lymphoma (NCI Cohort, dbGAPphs001444.v2.p1)**	N/A	E380fs	IRF-association	Truncation_Mutation
Diffuse Large B cell Lymphoma (NCI Cohort, dbGAPphs001444.v2.p1)**	N/A	D343G	IRF-association	Missense_Mutation
Diffuse Large B cell Lymphoma (NCI Cohort, dbGAPphs001444.v2.p1)**	N/A	N87H	DNA-binding	Missense_Mutation
Diffuse Large B cell Lymphoma (NCI Cohort, dbGAPphs001444.v2.p1)**	N/A	E74D	DNA-binding	Missense_Mutation
Diffuse Large B cell Lymphoma (NCI Cohort, dbGAPphs001444.v2.p1)**	N/A	G44D	DNA-binding	Missense_Mutation
Diffuse Large B cell Lymphoma (NCI Cohort, dbGAPphs001444.v2.p1)**	N/A	Q118K	DNA-binding	Missense_Mutation
Diffuse Large B cell Lymphoma (NCI Cohort, dbGAPphs001444.v2.p1)**	N/A	V426fs	C-terminus	Truncation_Mutation
Diffuse Large B cell Lymphoma (NCI Cohort, dbGAPphs001444.v2.p1)**	N/A	Q285K	IRF-association	Missense_Mutation
Diffuse Large B cell Lymphoma (NCI Cohort, dbGAPphs001444.v2.p1)**	N/A	E420fs	C-terminus	Truncation_Mutation
Diffuse Large B cell Lymphoma (NCI Cohort, dbGAPphs001444.v2.p1)**	N/A	A393fs	C-terminus	Truncation_Mutation
Diffuse Large B cell Lymphoma (NCI Cohort, dbGAPphs001444.v2.p1)**	N/A	S416X	C-terminus	Truncation_Mutation
Diffuse Large B cell Lymphoma (NCI Cohort, dbGAPphs001444.v2.p1)**	N/A	G7del	DNA-binding	In_Frame_Del
Diffuse Large B cell Lymphoma (NCI Cohort, dbGAPphs001444.v2.p1)**	N/A	E353K	IRF-association	Missense_Mutation
Diffuse Large B cell Lymphoma (NCI Cohort, dbGAPphs001444.v2.p1)**	N/A	Q423fs	C-terminus	Truncation_Mutation
Diffuse Large B cell Lymphoma (NCI Cohort, dbGAPphs001444.v2.p1)**	N/A	S55A	DNA-binding	Missense_Mutation
Diffuse Large B cell Lymphoma (NCI Cohort, dbGAPphs001444.v2.p1)**	N/A	I424fs	C-terminus	Truncation_Mutation
Diffuse Large B cell Lymphoma (NCI Cohort, dbGAPphs001444.v2.p1)**	N/A	R404fs	C-terminus	Truncation_Mutation
Diffuse Large B cell Lymphoma (NCI Cohort, dbGAPphs001444.v2.p1)**	N/A	F403fs	C-terminus	Truncation_Mutation
Diffuse Large B cell Lymphoma (NCI Cohort, dbGAPphs001444.v2.p1)**	N/A	Q401X	C-terminus	Truncation_Mutation
Diffuse Large B cell Lymphoma (NCI Cohort, dbGAPphs001444.v2.p1)**	N/A	G128A	DNA-binding	Missense_Mutation
Diffuse Large B cell Lymphoma (NCI Cohort, dbGAPphs001444.v2.p1)**	N/A	G44S	DNA-binding	Missense_Mutation
Diffuse Large B cell Lymphoma (NCI Cohort, dbGAPphs001444.v2.p1)**	N/A	Q50P	DNA-binding	Missense_Mutation
Diffuse Large B cell Lymphoma (NCI Cohort, dbGAPphs001444.v2.p1)**	N/A	N87Y	DNA-binding	Missense_Mutation
Diffuse Large B cell Lymphoma (NCI Cohort, dbGAPphs001444.v2.p1)**	N/A	T80A	DNA-binding	Missense_Mutation
Diffuse Large B cell Lymphoma (NCI Cohort, dbGAPphs001444.v2.p1)**	N/A	F403L	C-terminus	Missense_Mutation
Diffuse Large B cell Lymphoma (NCI Cohort, dbGAPphs001444.v2.p1)**	N/A	E395fs	C-terminus	Truncation_Mutation
Diffuse Large B cell Lymphoma (NCI Cohort, dbGAPphs001444.v2.p1)**	N/A	L82F	DNA-binding	Missense_Mutation
Diffuse Large B cell Lymphoma (NCI Cohort, dbGAPphs001444.v2.p1)**	N/A	X427fs	C-terminus	Truncation_Mutation
Diffuse Large B cell Lymphoma (NCI Cohort, dbGAPphs001444.v2.p1)**	N/A	D400G	C-terminus	Missense_Mutation
Diffuse Large B cell Lymphoma (NCI Cohort, dbGAPphs001444.v2.p1)**	N/A	E420X	C-terminus	Truncation_Mutation
Diffuse Large B cell Lymphoma (NCI Cohort, dbGAPphs001444.v2.p1)**	N/A	R268Q	IRF-association	Missense_Mutation
(36)	P22	E396*	C-terminus	Nonsense_Mutation
(36)	P25	S55A	DNA-binding	Missense_Mutation
(36)	P43	?	na	Nonstop_Mutation
(36)	P48	N421fs*32	C-terminus	Frameshift
(36)	P63	V390fs*79	C-terminus	Frameshift
(36)	P63	N87Y	DNA-binding	Missense_Mutation
(36)	P96	Q423fs*31	C-terminus	Frameshift
(36)	P162	N87Y	DNA-binding	Missense_Mutation
(36)	P170	Y23H	DNA-binding	Missense_Mutation
(36)	P172	T80A	DNA-binding	Missense_Mutation
(36)	P186	427E	C-terminus	Nonstop_Mutation
(36)	P190	E420*	C-terminus	Nonsense_Mutation
(36)	P193	Q392*	C-terminus	Nonsense_Mutation
(36)	P197	T80S	DNA-binding	Missense_Mutation
(36)	P207	427fs*26	C-terminus	Frameshift
(36)	P217	Q392*	C-terminus	Nonsense_Mutation
(36)	P217	Y23H	DNA-binding	Missense_Mutation
(36)	P220	E396*	C-terminus	Nonsense_Mutation
(36)	P223	K66R	DNA-binding	Missense_Mutation
(36)	P232	C385fs*54	C-terminus	Frameshift
(36)	P234	?	na	Nonstop_Mutation
(36)	P238	T80A	DNA-binding	Missense_Mutation
(36)	P246	Q422fs*30	C-terminus	Frameshift
(36)	P250	T80A	DNA-binding	Missense_Mutation
(36)	P298	E420*	C-terminus	Nonsense_Mutation
(36)	P303	Q392fs*58	C-terminus	Frameshift
(36)	P313	427fs*26	C-terminus	Frameshift
(36)	P324	Q422fs*31	C-terminus	Frameshift
(36)	P343	V426fs*27	C-terminus	Frameshift
(36)	P359	S416_I424delinsF	C-terminus	In_Frame_InDel
(36)	P368	?	na	Nonstop_Mutation
(36)	P371	427fs*26	C-terminus	Frameshift
(36)	P373	Q392*	C-terminus	Nonsense_Mutation
(36)	P383	Q423*	C-terminus	Nonsense_Mutation
(36)	P391	427Q	C-terminus	Nonstop_Mutation
(36)	P395	Y23H	DNA-binding	Missense_Mutation
(36)	P395	K66R	DNA-binding	Missense_Mutation
(36)	P438	?	na	Nonstop_Mutation
(36)	P443	N87Y	DNA-binding	Missense_Mutation
(36)	P456	Q181fs*133	IRF-association	Frameshift
(36)	P473	Q422fs*30	C-terminus	Frameshift
(36)	P486	P398fs*54	C-terminus	Frameshift
(36)	P488	Q392*	C-terminus	Nonsense_Mutation
(36)	P501	K66R	DNA-binding	Missense_Mutation
(36)	P504	I424F	C-terminus	Missense_Mutation
(36)	P517	S55A	DNA-binding	Missense_Mutation
(36)	P521	R404fs*49	C-terminus	Frameshift
(36)	P550	V390fs*79	C-terminus	Frameshift
(36)	P568	N421fs*32	C-terminus	Frameshift
(36)	P583	Y23H	DNA-binding	Missense_Mutation
(36)	P587	?	na	Nonstop_Mutation
(36)	P601	S416*	C-terminus	Nonsense_Mutation
(36)	P616	E420*	C-terminus	Nonsense_Mutation
(36)	P629	I424F	C-terminus	Missense_Mutation
(36)	P642	427fs*27	C-terminus	Frameshift
(36)	P650	S55A	DNA-binding	Missense_Mutation
(36)	P663	S416*	C-terminus	Nonsense_Mutation
(36)	P682	S55A	DNA-binding	Missense_Mutation
(36)	P684	Q422fs*31	C-terminus	Frameshift
(36)	P692	E420*	C-terminus	Nonsense_Mutation
(36)	P694	Q422fs*30	C-terminus	Frameshift
(36)	P696	T80A	DNA-binding	Missense_Mutation
(36)	P705	R404fs*49	C-terminus	Frameshift
(36)	P708	Y23H	DNA-binding	Missense_Mutation
(36)	P732	S55A	DNA-binding	Missense_Mutation
(36)	P733	K66R	DNA-binding	Missense_Mutation
(36)	P751	S55A	DNA-binding	Missense_Mutation
(36)	P755	S55A	DNA-binding	Missense_Mutation
(36)	P769	N87Y	DNA-binding	Missense_Mutation
(36)	P779	F403fs*66	C-terminus	Frameshift
(36)	P815	Q423*	C-terminus	Nonsense
(36)	P849	E420fs*33	C-terminus	Frameshift
(36)	P904	Y23H	DNA-binding	Missense_Mutation

[*] sample-specific variant description not provided (only gene-specific) [illegible]

** dbGAP Study Accession: phs001444.v2.p1- reanalized in this study

Table S3. IRF8 variant allele frequency (VAF) % in DLBCLs

Study of Origin	Sample ID	IRF8 Protein Change	VAF (%)	More than one
(40)	DLBCL_009	I424F	37%	
(40)	DLBCL_012	Q392*	15%	
(40)	DLBCL_019	Y23H	38%	Yes
(40)	DLBCL_019	S20R	38%	Yes
(40)	DLBCL_031	A76V	35%	
(40)	DLBCL_038	G7del	43%	
(40)	DLBCL_057	D91N	42%	
(40)	DLBCL_058	S55A	84%	
(40)	DLBCL_060	Y23H	12%	
(40)	DLBCL_120	S55A	23%	
(40)	DLBCL_124	T80A	35%	
(40)	DLBCL_135	E380Rfs*11	28%	
(40)	DLBCL_144	K66R	72%	Yes
(40)	DLBCL_144	L82F	73%	Yes
(40)	DLBCL_144	I424S	63%	Yes
Diffuse Large B cell Lymphoma (NCI Cohort, dbGAPphs001444.v2.p1*)	DLBCL10463	Glu353Lys	33%	
Diffuse Large B cell Lymphoma (NCI Cohort, dbGAPphs001444.v2.p1*)	DLBCL10478	Arg419fs	68%	
Diffuse Large B cell Lymphoma (NCI Cohort, dbGAPphs001444.v2.p1*)	DLBCL10488	Glu395fs	36%	Yes
Diffuse Large B cell Lymphoma (NCI Cohort, dbGAPphs001444.v2.p1*)	DLBCL10488	Gln118Lys	30%	Yes
Diffuse Large B cell Lymphoma (NCI Cohort, dbGAPphs001444.v2.p1*)	DLBCL10502	Gln392*	21%	
Diffuse Large B cell Lymphoma (NCI Cohort, dbGAPphs001444.v2.p1*)	DLBCL10512	Ter427Glnext*?	1%	
Diffuse Large B cell Lymphoma (NCI Cohort, dbGAPphs001444.v2.p1*)	DLBCL10516	Asn421fs	18%	
Diffuse Large B cell Lymphoma (NCI Cohort, dbGAPphs001444.v2.p1*)	DLBCL10518	Ter437fs	49%	Yes
Diffuse Large B cell Lymphoma (NCI Cohort, dbGAPphs001444.v2.p1*)	DLBCL10518	Val426fs	49%	Yes
Diffuse Large B cell Lymphoma (NCI Cohort, dbGAPphs001444.v2.p1*)	DLBCL10519	Tyr23His	26%	
Diffuse Large B cell Lymphoma (NCI Cohort, dbGAPphs001444.v2.p1*)	DLBCL10523	Thr80Ala	22%	Yes
Diffuse Large B cell Lymphoma (NCI Cohort, dbGAPphs001444.v2.p1*)	DLBCL10523	Glu420*	26%	Yes
Diffuse Large B cell Lymphoma (NCI Cohort, dbGAPphs001444.v2.p1*)	DLBCL10523	Asn421fs	26%	Yes
Diffuse Large B cell Lymphoma (NCI Cohort, dbGAPphs001444.v2.p1*)	DLBCL10527	Thr80Ala	13%	Yes
Diffuse Large B cell Lymphoma (NCI Cohort, dbGAPphs001444.v2.p1*)	DLBCL10527	Leu82Phe	13%	Yes
Diffuse Large B cell Lymphoma (NCI Cohort, dbGAPphs001444.v2.p1*)	DLBCL10527	Glu74Asp	16%	Yes
Diffuse Large B cell Lymphoma (NCI Cohort, dbGAPphs001444.v2.p1*)	DLBCL10532	Thr80Ala	39%	
Diffuse Large B cell Lymphoma (NCI Cohort, dbGAPphs001444.v2.p1*)	DLBCL10553	Asp91Asn	43%	
Diffuse Large B cell Lymphoma (NCI Cohort, dbGAPphs001444.v2.p1*)	DLBCL10554	Glu380fs	23%	
Diffuse Large B cell Lymphoma (NCI Cohort, dbGAPphs001444.v2.p1*)	DLBCL10782	Gly7del	52%	
Diffuse Large B cell Lymphoma (NCI Cohort, dbGAPphs001444.v2.p1*)	DLBCL10843	Asn87Tyr	41%	Yes
Diffuse Large B cell Lymphoma (NCI Cohort, dbGAPphs001444.v2.p1*)	DLBCL10843	Asp91Ala	42%	Yes
Diffuse Large B cell Lymphoma (NCI Cohort, dbGAPphs001444.v2.p1*)	DLBCL10894	Arg415fs	33%	Yes
Diffuse Large B cell Lymphoma (NCI Cohort, dbGAPphs001444.v2.p1*)	DLBCL10894	Ser416del	33%	Yes
Diffuse Large B cell Lymphoma (NCI Cohort, dbGAPphs001444.v2.p1*)	DLBCL10913	Ser416del	30%	
Diffuse Large B cell Lymphoma (NCI Cohort, dbGAPphs001444.v2.p1*)	DLBCL10940	Gln392*	40%	
Diffuse Large B cell Lymphoma (NCI Cohort, dbGAPphs001444.v2.p1*)	DLBCL10941	Gln392*	14%	
Diffuse Large B cell Lymphoma (NCI Cohort, dbGAPphs001444.v2.p1*)	DLBCL10950	Tyr23His	30%	
Diffuse Large B cell Lymphoma (NCI Cohort, dbGAPphs001444.v2.p1*)	DLBCL10965	Gln392*	22%	
Diffuse Large B cell Lymphoma (NCI Cohort, dbGAPphs001444.v2.p1*)	DLBCL10993	Asp400Gly	25%	
Diffuse Large B cell Lymphoma (NCI Cohort, dbGAPphs001444.v2.p1*)	DLBCL11181	Asn87Tyr	41%	
Diffuse Large B cell Lymphoma (NCI Cohort, dbGAPphs001444.v2.p1*)	DLBCL11191	Asn421fs	11%	
Diffuse Large B cell Lymphoma (NCI Cohort, dbGAPphs001444.v2.p1*)	DLBCL11430	Ser55Ala	23%	
Diffuse Large B cell Lymphoma (NCI Cohort, dbGAPphs001444.v2.p1*)	DLBCL11477	Phe403fs	57%	
Diffuse Large B cell Lymphoma (NCI Cohort, dbGAPphs001444.v2.p1*)	DLBCL11478	Gln401*	13%	
Diffuse Large B cell Lymphoma (NCI Cohort, dbGAPphs001444.v2.p1*)	DLBCL11479	Gln285Lys	21%	
Diffuse Large B cell Lymphoma (NCI Cohort, dbGAPphs001444.v2.p1*)	DLBCL11494	Ter427fs	11%	
Diffuse Large B cell Lymphoma (NCI Cohort, dbGAPphs001444.v2.p1*)	DLBCL11497	Ser55Ala	43%	
Diffuse Large B cell Lymphoma (NCI Cohort, dbGAPphs001444.v2.p1*)	DLBCL11507	Thr80Ala	47%	
Diffuse Large B cell Lymphoma (NCI Cohort, dbGAPphs001444.v2.p1*)	DLBCL11519	Gln118Lys	35%	
Diffuse Large B cell Lymphoma (NCI Cohort, dbGAPphs001444.v2.p1*)	DLBCL11526	Glu395fs	26%	
Diffuse Large B cell Lymphoma (NCI Cohort, dbGAPphs001444.v2.p1*)	DLBCL11532	Ter427Glnext*?	45%	
Diffuse Large B cell Lymphoma (NCI Cohort, dbGAPphs001444.v2.p1*)	DLBCL11541	Val426fs	36%	
Diffuse Large B cell Lymphoma (NCI Cohort, dbGAPphs001444.v2.p1*)	DLBCL11542	Gly44Ser	32%	Yes
Diffuse Large B cell Lymphoma (NCI Cohort, dbGAPphs001444.v2.p1*)	DLBCL11542	Gly44Asp	32%	Yes
Diffuse Large B cell Lymphoma (NCI Cohort, dbGAPphs001444.v2.p1*)	DLBCL11542	Ter427Glnext*?	28%	Yes
Diffuse Large B cell Lymphoma (NCI Cohort, dbGAPphs001444.v2.p1*)	DLBCL11563	Tyr23His	12%	
Diffuse Large B cell Lymphoma (NCI Cohort, dbGAPphs001444.v2.p1*)	DLBCL11568	Leu82Val	9%	
Diffuse Large B cell Lymphoma (NCI Cohort, dbGAPphs001444.v2.p1*)	DLBCL11578	Ser55Ala	89%	
Diffuse Large B cell Lymphoma (NCI Cohort, dbGAPphs001444.v2.p1*)	DLBCL11581	Arg276His	47%	
Diffuse Large B cell Lymphoma (NCI Cohort, dbGAPphs001444.v2.p1*)	DLBCL11589	Asn87Tyr	23%	
Diffuse Large B cell Lymphoma (NCI Cohort, dbGAPphs001444.v2.p1*)	DLBCL11590	Tyr23His	52%	
Diffuse Large B cell Lymphoma (NCI Cohort, dbGAPphs001444.v2.p1*)	DLBCL11672	Arg404fs	79%	
Diffuse Large B cell Lymphoma (NCI Cohort, dbGAPphs001444.v2.p1*)	DLBCL11675	Pro114Arg	59%	
Diffuse Large B cell Lymphoma (NCI Cohort, dbGAPphs001444.v2.p1*)	DLBCL11680	Ser55Ala	38%	
(12)	DLBCL-LS146	N131D	64%	
(12)	DLBCL-LS3309	P398Rfs*56	77%	
(12)	DLBCL-LS3615	I424N	19%	
(12)	DLBCL-LS4618	S55A	26%	
(12)	DLBCL-RICOVER_1106	T80A	40%	
(12)	DLBCL-RICOVER_1199	P398Rfs*56	15%	
(12)	DLBCL-RICOVER_197	S55A	56%	
(12)	DLBCL-RICOVER_267	G388Afs*3	52%	
(12)	DLBCL-RICOVER_950	Y23H	21%	
Diffuse Large B-Cell Lymphoma (TCGA, PanCancer Atlas)	TCGA-GS-A9TX-01	E395K	7%	
Diffuse Large B-Cell Lymphoma (TCGA, PanCancer Atlas)	TCGA-GS-A9TY-01	T80A	62%	
Diffuse Large B-Cell Lymphoma (TCGA, PanCancer Atlas)	TCGA-GS-A9TZ-01	D91N	36%	
Diffuse Large B-Cell Lymphoma (TCGA, PanCancer Atlas)	TCGA-RQ-A6JB-01	S55A	78%	

Table S4. Cell of origin (COO) classification of Diffuse Large B-Cell Lymphomas (DLBCLs) carrying IRF8 mutation from publicly available cohorts

Study of Origin	Sample ID	COO Class	Subgroup
(37)	695	GCB	
(37)	2075	GCB	
(37)	2076	GCB	
(37)	2154	GCB	
(37)	2190	Unclass	
(37)	2209	GCB	
(37)	2251	ABC	
(37)	2262	Unclass	
(37)	2459	GCB	
(37)	2522	ABC	
(37)	2530	ABC	
(37)	2533	GCB	
(37)	2640	GCB	
(37)	2680	Unclass	
(37)	2696	GCB	
(37)	2728	ABC	
(37)	2779	GCB	
(37)	2779	GCB	
(37)	2805	GCB	
(37)	2813	GCB	
(37)	2837	GCB	
(37)	2871	ABC	
(37)	2897	ABC	
(37)	2900	GCB	
(37)	2918	GCB	
(37)	2918	GCB	
(37)	2934	GCB	
(37)	2944	GCB	
(37)	2945	ABC	
(37)	2950	Unclass	
(37)	3386	GCB	
(37)	3417	ABC	
(37)	3460	GCB	
(37)	3485	GCB	
(37)	3632	GCB	
(37)	3635	GCB	
(37)	3647	GCB	
(37)	3647	GCB	
(37)	3697	ABC	
(37)	3737	GCB	
(37)	3740	GCB	
(37)	3745	GCB	
(37)	3750	ABC	
(37)	3758	GCB	
(37)	3812	GCB	
(37)	3835	GCB	
(37)	3851	GCB	
(37)	3856	GCB	
(37)	3856	GCB	
(37)	3892	Unclass	
(37)	3900	Unclass	
(37)	3901	GCB	
(37)	3909	GCB	
(37)	3936	GCB	
(37)	3943	Unclass	
(37)	3967	Unclass	
(37)	3969	GCB	
(37)	3972	GCB	
(40)	DLBCL_009	GCB	
(40)	DLBCL_012	ABC	
(40)	DLBCL_019	GCB	
(40)	DLBCL_019	GCB	
(40)	DLBCL_031	GCB	
(40)	DLBCL_038	GCB	
(40)	DLBCL_057	GCB	
(40)	DLBCL_058	GCB	
(40)	DLBCL_060	Unclass	
(40)	DLBCL_060	Unclass	
(40)	DLBCL_135	GCB	
Diffuse Large B cell Lymphoma (NCI Cohort, dbGAPphs001444.v2.p1)	DLBCL10463	ABC	MCD
Diffuse Large B cell Lymphoma (NCI Cohort, dbGAPphs001444.v2.p1)	DLBCL10478	GCB	ST2
Diffuse Large B cell Lymphoma (NCI Cohort, dbGAPphs001444.v2.p1)	DLBCL10488	Unclass	BN2
Diffuse Large B cell Lymphoma (NCI Cohort, dbGAPphs001444.v2.p1)	DLBCL10502	GCB	Other
Diffuse Large B cell Lymphoma (NCI Cohort, dbGAPphs001444.v2.p1)	DLBCL10512	GCB	EZB
Diffuse Large B cell Lymphoma (NCI Cohort, dbGAPphs001444.v2.p1)	DLBCL10516	ABC	Other
Diffuse Large B cell Lymphoma (NCI Cohort, dbGAPphs001444.v2.p1)	DLBCL10518	GCB	EZB
Diffuse Large B cell Lymphoma (NCI Cohort, dbGAPphs001444.v2.p1)	DLBCL10519	GCB	EZB
Diffuse Large B cell Lymphoma (NCI Cohort, dbGAPphs001444.v2.p1)	DLBCL10523	GCB	EZB
Diffuse Large B cell Lymphoma (NCI Cohort, dbGAPphs001444.v2.p1)	DLBCL10527	Unclass	Other
Diffuse Large B cell Lymphoma (NCI Cohort, dbGAPphs001444.v2.p1)	DLBCL10532	ABC	MCD
Diffuse Large B cell Lymphoma (NCI Cohort, dbGAPphs001444.v2.p1)	DLBCL10553	GCB	ST2
Diffuse Large B cell Lymphoma (NCI Cohort, dbGAPphs001444.v2.p1)	DLBCL10554	GCB	EZB
Diffuse Large B cell Lymphoma (NCI Cohort, dbGAPphs001444.v2.p1)	DLBCL10782	GCB	Mixed
Diffuse Large B cell Lymphoma (NCI Cohort, dbGAPphs001444.v2.p1)	DLBCL10843	ABC	BN2
Diffuse Large B cell Lymphoma (NCI Cohort, dbGAPphs001444.v2.p1)	DLBCL10894	ABC	Other
Diffuse Large B cell Lymphoma (NCI Cohort, dbGAPphs001444.v2.p1)	DLBCL10913	ABC	BN2
Diffuse Large B cell Lymphoma (NCI Cohort, dbGAPphs001444.v2.p1)	DLBCL10940	ABC	MCD
Diffuse Large B cell Lymphoma (NCI Cohort, dbGAPphs001444.v2.p1)	DLBCL10941	ABC	Mixed
Diffuse Large B cell Lymphoma (NCI Cohort, dbGAPphs001444.v2.p1)	DLBCL10950	ABC	Other
Diffuse Large B cell Lymphoma (NCI Cohort, dbGAPphs001444.v2.p1)	DLBCL10965	ABC	MCD
Diffuse Large B cell Lymphoma (NCI Cohort, dbGAPphs001444.v2.p1)	DLBCL10993	ABC	BN2
Diffuse Large B cell Lymphoma (NCI Cohort, dbGAPphs001444.v2.p1)	DLBCL11181	Unclass	BN2
Diffuse Large B cell Lymphoma (NCI Cohort, dbGAPphs001444.v2.p1)	DLBCL11191	Unclass	BN2
Diffuse Large B cell Lymphoma (NCI Cohort, dbGAPphs001444.v2.p1)	DLBCL11430	ABC	Other
Diffuse Large B cell Lymphoma (NCI Cohort, dbGAPphs001444.v2.p1)	DLBCL11477	Unclass	BN2
Diffuse Large B cell Lymphoma (NCI Cohort, dbGAPphs001444.v2.p1)	DLBCL11478	Unclass	BN2
Diffuse Large B cell Lymphoma (NCI Cohort, dbGAPphs001444.v2.p1)	DLBCL11479	Unclass	Other
Diffuse Large B cell Lymphoma (NCI Cohort, dbGAPphs001444.v2.p1)	DLBCL11494	Unclass	BN2
Diffuse Large B cell Lymphoma (NCI Cohort, dbGAPphs001444.v2.p1)	DLBCL11497	ABC	EZB
Diffuse Large B cell Lymphoma (NCI Cohort, dbGAPphs001444.v2.p1)	DLBCL11507	Unclass	A53
Diffuse Large B cell Lymphoma (NCI Cohort, dbGAPphs001444.v2.p1)	DLBCL11519	GCB	Other
Diffuse Large B cell Lymphoma (NCI Cohort, dbGAPphs001444.v2.p1)	DLBCL11526	GCB	EZB
Diffuse Large B cell Lymphoma (NCI Cohort, dbGAPphs001444.v2.p1)	DLBCL11532	GCB	EZB
Diffuse Large B cell Lymphoma (NCI Cohort, dbGAPphs001444.v2.p1)	DLBCL11541	GCB	Other
Diffuse Large B cell Lymphoma (NCI Cohort, dbGAPphs001444.v2.p1)	DLBCL11542	GCB	EZB
Diffuse Large B cell Lymphoma (NCI Cohort, dbGAPphs001444.v2.p1)	DLBCL11563	GCB	EZB
Diffuse Large B cell Lymphoma (NCI Cohort, dbGAPphs001444.v2.p1)	DLBCL11568	GCB	EZB
Diffuse Large B cell Lymphoma (NCI Cohort, dbGAPphs001444.v2.p1)	DLBCL11578	GCB	Other
Diffuse Large B cell Lymphoma (NCI Cohort, dbGAPphs001444.v2.p1)	DLBCL11581	GCB	EZB
Diffuse Large B cell Lymphoma (NCI Cohort, dbGAPphs001444.v2.p1)	DLBCL11589	GCB	EZB
Diffuse Large B cell Lymphoma (NCI Cohort, dbGAPphs001444.v2.p1)	DLBCL11590	GCB	EZB
Diffuse Large B cell Lymphoma (NCI Cohort, dbGAPphs001444.v2.p1)	DLBCL11672	GCB	EZB
Diffuse Large B cell Lymphoma (NCI Cohort, dbGAPphs001444.v2.p1)	DLBCL11675	GCB	EZB
Diffuse Large B cell Lymphoma (NCI Cohort, dbGAPphs001444.v2.p1)	DLBCL11680	GCB	EZB
(12)	DLBCL-LS146	GCB	A53
(12)	DLBCL-LS3309	GCB	EZB/A53
(12)	DLBCL-LS3615	Unclass	Other
(12)	DLBCL-LS4618	GCB	EZB
(12)	DLBCL-RICOVER_1106	ABC	MCD
(12)	DLBCL-RICOVER_197	GCB	EZB
(12)	DLBCL-RICOVER_267	GCB	EZB
(7, 38, 41)	DLC0041	GCB	EZB
(7, 38, 41)	DLC0047	GCB	EZB
(7, 38, 41)	DLC0087	GCB	EZB
(7, 38, 41)	DLC0097	GCB	EZB/ST2/A53
(7, 38, 41)	DLC0105	Unclass	Other
(7, 38, 41)	DLC0123	Unclass	Other
(7, 38, 41)	DLC0134	GCB	EZB
(7, 38, 41)	DLC0155	GCB	EZB
(7, 38, 41)	DLC0161	GCB	Other
(7, 38, 41)	DLC0169	GCB	ST2
(7, 38, 41)	DLC0183	Unclass	ST2
(7, 38, 41)	DLC0194	GCB	EZB
(7, 38, 41)	DLC0210	ABC	N1
(7, 38, 41)	DLC0236	GCB	EZB
(7, 38, 41)	DLC0246	GCB	BN2/MCD
(7, 38, 41)	DLC0252	GCB	EZB/A53
(7, 38, 41)	DLC0255	GCB	Other
(7, 38, 41)	DLC0256	GCB	EZB
(7, 38, 41)	DLC0267	GCB	EZB/A53
(7, 38, 41)	DLC0269	GCB	BN2
(7, 38, 41)	DLC0270	GCB	EZB
(7, 38, 41)	DLC0280	GCB	EZB/ST2/A53
(7, 38, 41)	DLC0310	GCB	BN2/EZB
(7, 38, 41)	DLC0317	ABC	Other
(7, 38, 41)	DLC0325	GCB	ST2
(7, 38, 41)	DLC0326	GCB	EZB
(7, 38, 41)	DLC0353	GCB	EZB
(7, 38, 41)	DLC0370	ABC	EZB
(7, 38, 41)	DLC0371	GCB	EZB
(7, 38, 41)	DLC0374	GCB	EZB
(7, 38, 41)	DLC0380	GCB	EZB
(7, 38, 41)	DLC0382	GCB	EZB
(7, 38, 41)	DLC0393	GCB	EZB
(7, 38, 41)	DLC0397	Unclass	ST2/A53
Diffuse Large B-Cell Lymphoma (TCGA, PanCancer Atlas)	TCGA-GS-A9TX	GCB	Other
Diffuse Large B-Cell Lymphoma (TCGA, PanCancer Atlas)	TCGA-GS-A9TZ	GCB	ST2
Diffuse Large B-Cell Lymphoma (TCGA, PanCancer Atlas)	TCGA-RQ-A6JB	GCB	EZB
(12)	DLBCL-RICOVER_1199	N/A	EZB
(12)	DLBCL-RICOVER_950	N/A	Other

Table S5. Summary of IRF8 mutation rates in published cohorts of other mature B-cell malignancies

Tumor type	Total samples	Samples with IRF8 mutations	% IRF8 mut samples	Fisher's exact test (v. DLBCLs)	Notes (source data)
Chronic Lymphocytic Leukemia	537	1	0.19		*(90)*
Chronic Lymphocytic Leukemia	506	1	0.20		*(91)*
Chronic lymphocytic leukemia	105	0	0.00		*(89)*
**Chronic Lymphocytic Leukemia (combined)**	**1148**	**2**	**0.17**	**< 0.00001**	
Multiple Myeloma	211	2	0.95		*(88)*
**Multiple Myeloma (combined)**	**211**	**2**	**0.95**	**0**	
Burkitt lymphoma	241	22	9.13		*(45)*
Burkitt lymphoma	101	7	6.93		*(44)*
**Burkitt lymphoma (combined)**	**342**	**29**	**8.48**	**0.594**	
Marginal zone lymphoma	20	1	5.00		*(46)*
Marginal zone lymphoma	34	2	5.88		*(46)*
Marginal zone lymphoma	12	1	8.33		*(46)*
Marginal zone lymphoma	74	2	2.70		*(46)*
Marginal zone lymphoma	38	1	2.63		*(46)*
**Marginal zone lymphoma (combined)**	**178**	**7**	**3.93**	**0.0768**	
follicular lymphoma	113	15	13.27		*(42)*
follicular lymphoma	55	7	12.73		*(43)*
**Follicular lymphoma (combined)**	**168**	**22**	**13.10**	**0.01**	

Table S6. List of IRF8 mutations in published cohorts of other mature B-cell malignancies

Diagnosis	sample ID	trv_type	amino_acid_change	IRF8 Domain	Source
Follicular lymphoma	LYM036-Naive	nonsense	p.Q392*	C-terminus	*(42)*
Follicular lymphoma	LYM045-Naïve	nonsense	p.L124*	DNA-binding domain	*(42)*
Follicular lymphoma	LYM045-Naïve	missense	p.C84R	DNA-binding domain	*(42)*
Follicular lymphoma	LYM045-Naïve	splice_region	e2-8	?	*(42)*
Follicular lymphoma	LYM117-Treated	frame_shift_de	p.V402fs	C-terminus	*(42)*
Follicular lymphoma	LYM177-Treated	missense	p.S55A	DNA-binding domain	*(42)*
Follicular lymphoma	LYM238-Sorted	frame_shift_de	p.R415fs	C-terminus	*(42)*
Follicular lymphoma	LYM238-Naive	frame_shift_de	p.R415fs	C-terminus	*(42)*
Follicular lymphoma	FLX011-Naive	missense	p.K66R	DNA-binding domain	*(42)*
Follicular lymphoma	FLX023-Naive	missense	p.C299Y	IRF association	*(42)*
Follicular lymphoma	FLX024-Naive	missense	p.Y107H	DNA-binding domain	*(42)*
Follicular lymphoma	FLX038-Naive	missense	p.K66R	DNA-binding domain	*(42)*
Follicular lymphoma	FLX038-Naive	missense	p.E115K	DNA-binding domain	*(42)*
Follicular lymphoma	FLX048-Naive	missense	p.G344S	IRF association	*(42)*
Follicular lymphoma	FLX049-Naive	missense	p.R340W	IRF association	*(42)*
Follicular lymphoma	FLX071-Naive	frame_shift_in:	p.Q414fs	C-terminus	*(42)*
Follicular lymphoma	FLX073-Naive	nonstop	p.*427Q	C-terminus	*(42)*
Follicular lymphoma	FLX082-Naive	missense	p.E420K	C-terminus	*(42)*
Follicular lymphoma	L62	nonstop	427X/K	C-terminus	*(43)*
Follicular lymphoma	L62	missense	115E/K	DNA-binding domain	*(43)*
Follicular lymphoma	ML55	frame_shift_de	Frameshift deletion	?	*(43)*
Follicular lymphoma	ML51	nonsense	395E>X	C-terminus	*(43)*
Follicular lymphoma	FL3	missense	55S/A	DNA-binding domain	*(43)*
Follicular lymphoma	FL19	missense	115E/K	DNA-binding domain	*(43)*
Follicular lymphoma	FL27	nonsense	392Q/X	C-terminus	*(43)*
Follicular lymphoma	FL51	frame_shift_de	Frameshift deletion	?	*(43)*
Follicular lymphoma	ML55/FL8	missense	267T/M	IRF association	*(43)*
Marginal zone B-cell lymphoma	08_136	Missense	p.T96A	DNA-binding domain	*(46)*
Marginal zone B-cell lymphoma	OL2	Nonsense	p.E167X	IRF association	*(46)*
Marginal zone B-cell lymphoma	S3	Missense	p.T80A	DNA-binding domain	*(46)*
Marginal zone B-cell lymphoma	OL2	nonframeshift	p.P166_E167insIE	IRF association	*(46)*
Marginal zone B-cell lymphoma	12T_Nodal	Missense	p.S55A	DNA-binding domain	*(46)*
Marginal zone B-cell lymphoma	30	Missense	p.T96M	DNA-binding domain	*(46)*
Marginal zone B-cell lymphoma	21D	Missense	p.A179V	IRF association	*(46)*
Burkitt lymphoma	1289	Missense	p.Y23H	DNA-binding domain	*(44)*
Burkitt lymphoma	4949	Missense	p.Q118K	DNA-binding domain	*(44)*
Burkitt lymphoma	2973	Missense	p.Q118R	DNA-binding domain	*(44)*
Burkitt lymphoma	1288	Missense	p.D356N	IRF association	*(44)*
Burkitt lymphoma	4946	frame_shift	p.I424fs	C-terminus	*(44)*
Burkitt lymphoma	1060	frame_shift	p.T425fs	C-terminus	*(44)*
Chronic Lymphocytic Leukemia	DFCI-CLL120-Tumor	Missense	T80A	DNA-binding domain	*(90)*
Chronic Lymphocytic Leukemia	cll_iuopa_2015_1163	frame_shift_de	T425del	C-terminus	*(91)*
Multiple Myeloma	MM-0468	Missense	T222P	IRF association	*(88)*
Multiple Myeloma	MM-0322	Missense	R170Q	IRF association	*(88)*
Burkitt lymphoma	MU69903008	Missense	I424F	C-terminus	*(45)*
Burkitt lymphoma	MU84439784	Missense	T22A	DNA-binding domain	*(45)*
Burkitt lymphoma	MU67456258	Missense	S55A	DNA-binding domain	*(45)*
Burkitt lymphoma	MU1879565	Missense	Y23H	DNA-binding domain	*(45)*
Burkitt lymphoma	MU86030292	Frameshift	*427C	C-terminus	*(45)*
Burkitt lymphoma	MU85813334	Stop Gained	S416*	C-terminus	*(45)*
Burkitt lymphoma	MU86903725	Stop Gained	E420*	C-terminus	*(45)*
Burkitt lymphoma	MU68541625	Missense	P106S	DNA-binding domain	*(45)*
Burkitt lymphoma	MU82150161	Frameshift	P398D	IRF association	*(45)*
Burkitt lymphoma	MU86128862	Missense	K66R	DNA-binding domain	*(45)*
Burkitt lymphoma	MU82820511	Frameshift	A387fs	C-terminus	*(45)*
Burkitt lymphoma	MU86643116	Missense	E353K	IRF association	*(45)*
Burkitt lymphoma	MU86198647	Missense	I27S	DNA-binding domain	*(45)*
Burkitt lymphoma	MU82416405	Missense	F57L	DNA-binding domain	*(45)*
Burkitt lymphoma	MU68935112	Missense	V426G	C-terminus	*(45)*
Burkitt lymphoma	MU86109699	Missense	C26R	DNA-binding domain	*(45)*
Burkitt lymphoma	MU87262274	Frameshift	F147fs	IRF association	*(45)*
Burkitt lymphoma	MU87005070	Frameshift	L143fs	IRF association	*(45)*
Burkitt lymphoma	MU81838057	Missense	Q118K	IRF association	*(45)*

Table S7. IRF8 mutations reported in Diffuse Large B-Cell Lymphomas (DLBCLs) from NCI cohort, reanalyzed from dbGAPphs001444.v2.p1

Sample ID	Cancer Type	Transcript	Protein Change	Mutation Type	Chromosome	Start Position	End Position	Reference Allele	Variant Allele	Read Count Ref	Read Count Variant	Variant Freq (%)	Variant Class
DLBCL10463	DLBCL	NM_002163.4	Glu353Lys	Missense_Mutation	chr16	85920177	85920177	G	A	98	48	32.88	Missense
DLBCL10478	DLBCL	NM_002163.4	Arg419fs	FS	chr16	85921249	85921250	-	T	96	204	68.00	Disruptive
DLBCL10488	DLBCL	NM_002163.4	Glu395fs	FS	chr16	85921178	85921178	C	-	110	62	36.05	Disruptive
DLBCL10488	DLBCL	NM_002163.4	Gln118Lys	Missense_Mutation	chr16	85909167	85909167	C	A	146	63	30.14	Missense
DLBCL10502	DLBCL	NM_002163.4	Gln392*	stop_gained	chr16	85921175	85921175	C	T	129	35	21.34	Disruptive
DLBCL10512	DLBCL	NM_002163.4	Ter427Glnext*?	FS	chr16	85921280	85921280	T	C	849	11	1.28	Disruptive
DLBCL10516	DLBCL	NM_002163.4	Asn421fs	FS	chr16	85921259	85921259	A	-	725	160	18.08	Disruptive
DLBCL10518	DLBCL	NM_002163.4,NI	Val426fs,Ter437fs	FS	chr16	85921275	85921287	TCTAAGTG(	-	275	267	49.26	Disruptive
DLBCL10519	DLBCL	NM_002163.4	Tyr23His	Missense_Mutation	chr16	85903082	85903082	T	C	303	104	25.55	Missense
DLBCL10523	DLBCL	NM_002163.4	Thr80Ala	Missense_Mutation	chr16	85909053	85909053	A	G	89	25	21.93	Missense
DLBCL10523	DLBCL	NM_002163.4	Asn421fs	FS	chr16	85921259	85921259	A	-	99	34	25.56	Disruptive
DLBCL10527	DLBCL	NM_002163.4	Thr80Ala	Missense_Mutation	chr16	85909053	85909053	A	G	136	21	13.38	Missense
DLBCL10527	DLBCL	NM_002163.4	Leu82Phe	Missense_Mutation	chr16	85909061	85909061	A	C	136	21	13.38	Missense
DLBCL10527	DLBCL	NM_002163.4	Glu74Asp	Missense_Mutation	chr16	85909037	85909037	A	T	212	40	15.87	Missense
DLBCL10532	DLBCL	NM_002163.4	Thr80Ala	Missense_Mutation	chr16	85909053	85909053	A	G	97	62	38.99	Missense
DLBCL10553	DLBCL	NM_002163.4	Asp91Asn	Missense_Mutation	chr16	85909086	85909086	G	A	36	27	42.86	Missense
DLBCL10554	DLBCL	NM_002163.4	Glu380fs	FS	chr16	85921138	85921138	G	-	141	41	22.53	Disruptive
DLBCL10782	DLBCL	NM_002163.4	Gly7del	In_Frame_Del	chr16	85903029	85903031	GGT	-	210	229	52.16	Disruptive
DLBCL10843	DLBCL	NM_002163.4	Asn87Tyr	Missense_Mutation	chr16	85909074	85909074	A	T	143	98	40.66	Missense
DLBCL10843	DLBCL	NM_002163.4	Asp91Ala	Missense_Mutation	chr16	85909087	85909087	A	C	139	100	41.84	Missense
DLBCL10894	DLBCL	NM_002163.4	Arg415fs	FS	chr16	85921243	85921265	GATCATTTT	-	373	187	33.39	Disruptive
DLBCL10913	DLBCL	NM_002163.4	Ser416del	In_Frame_Del	chr16	85921245	85921247	CAT	-	398	171	30.05	Disruptive
DLBCL10940	DLBCL	NM_002163.4	Gln392*	stop_gained	chr16	85921175	85921175	C	T	181	119	39.67	Disruptive
DLBCL10941	DLBCL	NM_002163.4	Gln392*	stop_gained	chr16	85921175	85921175	C	T	160	25	13.51	Disruptive
DLBCL10950	DLBCL	NM_002163.4	Tyr23His	Missense_Mutation	chr16	85903082	85903082	T	C	311	133	29.95	Missense
DLBCL10965	DLBCL	NM_002163.4	Gln392*	stop_gained	chr16	85921175	85921175	C	T	200	57	22.18	Disruptive
DLBCL10993	DLBCL	NM_002163.4	Asp400Gly	Missense_Mutation	chr16	85921200	85921200	A	G	136	46	25.27	Missense
DLBCL11181	DLBCL	NM_002163.4	Asn87Tyr	Missense_Mutation	chr16	85909074	85909074	A	T	72	50	40.98	Missense
DLBCL11191	DLBCL	NM_002163.4	Asn421fs	FS	chr16	85921259	85921259	A	-	232	114	32.95	Disruptive
DLBCL11430	DLBCL	NM_002163.4	Ser55Ala	Missense_Mutation	chr16	85903178	85903178	T	G	182	53	22.55	Missense
DLBCL11477	DLBCL	NM_002163.4	Phe403fs	FS	chr16	85921207	85921207	T	-	71	96	57.49	Disruptive
DLBCL11478	DLBCL	NM_002163.4	Gln401*	stop_gained	chr16	85921202	85921202	C	T	129	19	12.84	Disruptive
DLBCL11479	DLBCL	NM_002163.4	Gln285Lys	Missense_Mutation	chr16	85918668	85918668	C	A	319	84	20.84	Missense
DLBCL11494	DLBCL	NM_002163.4	Ter427fs	FS	chr16	85921279	85921280	-	TT	631	76	10.75	Disruptive
DLBCL11497	DLBCL	NM_002163.4	Ser55Ala	Missense_Mutation	chr16	85903178	85903178	T	G	161	119	42.50	Missense
DLBCL11507	DLBCL	NM_002163.4	Thr80Ala	Missense_Mutation	chr16	85909053	85909053	A	G	10	9	47.37	Missense
DLBCL11519	DLBCL	NM_002163.4	Gln118Lys	Missense_Mutation	chr16	85909167	85909167	C	A	87	47	35.07	Missense
DLBCL11526	DLBCL	NM_002163.4	Glu395fs	FS	chr16	85921178	85921178	C	-	45	16	26.23	Disruptive
DLBCL11532	DLBCL	NM_002163.4	Ter427Glnext*?	FS	chr16	85921280	85921280	T	C	208	171	45.12	Disruptive
DLBCL11541	DLBCL	NM_002163.4	Val426fs	FS	chr16	85921274	85921274	C	-	150	85	36.17	Disruptive
DLBCL11542	DLBCL	NM_002163.4	Gly44Asn	Missense_Mutation	chr16	85903145	85903146	GG	AA	268	125	31.81	Missense
DLBCL11542	DLBCL	NM_002163.4	Ter427Glnext*?	FS	chr16	85921280	85921280	T	C	480	191	28.46	Disruptive
DLBCL11563	DLBCL	NM_002163.4	Tyr23His	Missense_Mutation	chr16	85903082	85903082	T	C	380	50	11.63	Missense
DLBCL11568	DLBCL	NM_002163.4	Leu82Val	Missense_Mutation	chr16	85909059	85909059	T	G	99	10	9.17	Missense
DLBCL11578	DLBCL	NM_002163.4	Ser55Ala	Missense_Mutation	chr16	85903178	85903178	T	G	47	375	88.86	Missense
DLBCL11581	DLBCL	NM_002163.4	Arg276His	Missense_Mutation	chr16	85918642	85918642	G	A	144	127	46.86	Missense
DLBCL11589	DLBCL	NM_002163.4	Asn87Tyr	Missense_Mutation	chr16	85909074	85909074	A	T	80	24	23.08	Missense
DLBCL11590	DLBCL	NM_002163.4	Tyr23His	Missense_Mutation	chr16	85903082	85903082	T	C	176	193	52.30	Missense
DLBCL11672	DLBCL	NM_002163.4	Arg404fs	FS	chr16	85921209	85921209	C	-	65	250	79.37	Disruptive
DLBCL11675	DLBCL	NM_002163.4	Pro114Arg	Missense_Mutation	chr16	85909156	85909156	C	G	24	34	58.62	Missense
DLBCL11680	DLBCL	NM_002163.4	Ser55Ala	Missense_Mutation	chr16	85903178	85903178	T	G	273	167	37.95	Missense

Supplemental Table 8

**Oligonucleotides**	Forward	Reverse
Human *IRF8*	GATGTGTGACCGGAATGGT	GCTCTTCCTCAGGAACAATTCG
Human *CD74*	CGCGACCTTATCTCCAACAATG	GGATGGAAAAGCCTGTGTACAG
Human *CIITA*	AGCCTTTCAAAGCCAAGTCC	TTGTTCTCACTCAGCGCATC
Human *TBP*	TATAATCCCAAGCGGTTTGCTGCG	AATTGTTGGTGGGTGAGCACAAGG
Human *HLA-DRA*	TTTCCGCAAGTTCCACTATCTCCC	AATAATGATGCCCACCAGACCCAC
Human *HLA-DRB1*	GCGGTTCCTGGACAGATACTT	CTGGGTCTTTGAAGGATATACAG
Human *HLA-DMB*	CTCTCACAGCACCTCAACC	TAGAAGCCCCACACATAGCA
Mouse *cd74*	ATGAGCAAGAACTCCCTGGAG	CAGGCCAGAAGATAGGTCTTC
Mouse *ciita*	TCTCGTGCAGACCCAGAG	AGGCTGGAAGGATCTTTGC
Mouse h*2-dmb2*	GTGAAGGTCTCTGTGTGTCTGC	TTCTGCTTTCTAGTGCCGTC
Mouse *tbp*	CTGGAATTGACCGCAGCTT	CAGTTGTCCGTGGCTCTCTT
Cd74 promoter	CCCCTTCATCCTGCCCAG	CCCACAGCAGGTCACTCC
H2-Dmb2 promoter	GACTCTAAAACCCTCCGTACATG	GTGAAACCTCTTGAGACACCAC
Ciita promoter	GTGTCCAATGCAATTATCATTTCAG	AGCCACCACAGCTTCTTATG
H2-Aa promoter	CAAAGCTGGAAAGAGAGCAG	TTTACAGGCGTGAATAGGAG
Negative ctrl - ChIP	TGTGTATCTGTGGGTTTGTGC	TCTATGACTCTAGCTCCTGGG
**Guide RNAs**		
Human *IRF8* g1	CACCGGTGGTCGGCGGCTTCGACAG	
Human *IRF8* g2	CACCGGAAGCCGCCGACCACCATTC	
Mouse *Irf8* g1	CACCGAGACCATGTTCCGTATCCCC	
Mouse *Irf8* g2	CACCGCGGCAAGCAGGATTACAATC	

Mouse *Irf8* g3	CACCGGCAGCCGCCGCCCGCCGTTC	
Mouse *Irf8* g5	CACCGGTTCTTCCTCGGGGACAATT	
Mouse *cd74* g2	CACCGGTACACCGGTGTCTCTGTCC	
Mouse *cd74* g1	CACCGTCGCATGAAGCTTCCGAAAT	

REFERENCES AND NOTES

1. V. Thorsson, D. L. Gibbs, S. D. Brown, D. Wolf, D. S. Bortone, T. H. Ou Yang, E. Porta-Pardo, G. F. Gao, C. L. Plaisier, J. A. Eddy, E. Ziv, A. C. Culhane, E. O. Paull, I. K. A. Sivakumar, A. J. Gentles, R. Malhotra, F. Farshidfar, A. Colaprico, J. S. Parker, L. E. Mose, N. S. Vo, J. Liu, Y. Liu, J. Rader, V. Dhankani, S. M. Reynolds, R. Bowlby, A. Califano, A. D. Cherniack, D. Anastassiou, D. Bedognetti, Y. Mokrab, A. M. Newman, A. Rao, K. Chen, A. Krasnitz, H. Hu, T. M. Malta, H. Noushmehr, C. S. Pedamallu, S. Bullman, A. I. Ojesina, A. Lamb, W. Zhou, H. Shen, T. K. Choueiri, J. N. Weinstein, J. Guinney, J. Saltz, R. A. Holt, C. S. Rabkin, Cancer Genome Atlas Research Network, A. J. Lazar, J. S. Serody, E. G. Demicco, M. L. Disis, B. G. Vincent, I. Shmulevich, The immune landscape of cancer. *Immunity* **48**, 812–830.e14 (2018).

2. M. D. Wellenstein, K. E. de Visser, Cancer-cell-intrinsic mechanisms shaping the tumor immune landscape. *Immunity* **48**, 399–416 (2018).

3. P. Sharma, S. Goswami, D. Raychaudhuri, B. A. Siddiqui, P. Singh, A. Nagarajan, J. Liu, S. K. Subudhi, C. Poon, K. L. Gant, S. M. Herbrich, S. Anandhan, S. Islam, M. Amit, G. Anandappa, J. P. Allison, Immune checkpoint therapy-current perspectives and future directions. *Cell* **186**, 1652–1669 (2023).

4. M. Challa-Malladi, Y. K. Lieu, O. Califano, A. B. Holmes, G. Bhagat, V. V. Murty, D. Dominguez-Sola, L. Pasqualucci, R. Dalla-Favera, Combined genetic inactivation of β2-microglobulin and CD58 reveals frequent escape from immune recognition in diffuse large B cell lymphoma. *Cancer Cell* **20**, 728–740 (2011).

5. C. Steidl, S. P. Shah, B. W. Woolcock, L. Rui, M. Kawahara, P. Farinha, N. A. Johnson, Y. Zhao, A. Telenius, S. B. Neriah, A. McPherson, B. Meissner, U. C. Okoye, A. Diepstra, A. van den Berg, M. Sun, G. Leung, S. J. Jones, J. M. Connors, D. G. Huntsman, K. J. Savage, L. M. Rimsza, D. E. Horsman, L. M. Staudt, U. Steidl, M. A. Marra, R. D. Gascoyne, MHC class II transactivator CIITA is a recurrent gene fusion partner in lymphoid cancers. *Nature* **471**, 377–381 (2011).

6. A. Mottok, B. Woolcock, F. C. Chan, K. M. Tong, L. Chong, P. Farinha, A. Telenius, E. Chavez, S. Ramchandani, M. Drake, M. Boyle, S. Ben-Neriah, D. W. Scott, L. M. Rimsza, R. Siebert, R. D. Gascoyne, C. Steidl, Genomic alterations in CIITA are frequent in primary mediastinal large B cell

lymphoma and are associated with diminished MHC class II expression. *Cell Rep.* **13**, 1418–1431 (2015).

7. D. Ennishi, K. Takata, W. Beguelin, G. Duns, A. Mottok, P. Farinha, A. Bashashati, S. Saberi, M. Boyle, B. Meissner, S. Ben-Neriah, B. W. Woolcock, A. Telenius, D. Lai, M. Teater, R. Kridel, K. J. Savage, L. H. Sehn, R. D. Morin, M. A. Marra, S. P. Shah, J. M. Connors, R. D. Gascoyne, D. W. Scott, A. M. Melnick, C. Steidl, Molecular and genetic characterization of MHC deficiency identifies EZH2 as therapeutic target for enhancing immune recognition. *Cancer Discov.* **9**, 546–563 (2019).

8. H. Hashwah, C. A. Schmid, S. Kasser, K. Bertram, A. Stelling, M. G. Manz, A. Muller, Inactivation of CREBBP expands the germinal center B cell compartment, down-regulates MHCII expression and promotes DLBCL growth. *Proc. Natl. Acad. Sci. U.S.A.* **114**, 9701–9706 (2017).

9. M. R. Green, S. Kihira, C. L. Liu, R. V. Nair, R. Salari, A. J. Gentles, J. Irish, H. Stehr, C. Vicente-Duenas, I. Romero-Camarero, I. Sanchez-Garcia, S. K. Plevritis, D. A. Arber, S. Batzoglou, R. Levy, A. A. Alizadeh, Mutations in early follicular lymphoma progenitors are associated with suppressed antigen presentation. *Proc. Natl. Acad. Sci. U.S.A.* **112**, E1116–1125 (2015).

10. R. D. Morin, S. E. Arthur, D. J. Hodson, Molecular profiling in diffuse large B-cell lymphoma: Why so many types of subtypes? *Br. J. Haematol.* **196**, 814–829 (2022).

11. D. Ennishi, E. D. Hsi, C. Steidl, D. W. Scott, Toward a new molecular taxonomy of diffuse large B-cell lymphoma. *Cancer Discov.* **10**, 1267–1281 (2020).

12. B. Chapuy, C. Stewart, A. J. Dunford, J. Kim, A. Kamburov, R. A. Redd, M. S. Lawrence, M. G. M. Roemer, A. J. Li, M. Ziepert, A. M. Staiger, J. A. Wala, M. D. Ducar, I. Leshchiner, E. Rheinbay, A. Taylor-Weiner, C. A. Coughlin, J. M. Hess, C. S. Pedamallu, D. Livitz, D. Rosebrock, M. Rosenberg, A. A. Tracy, H. Horn, P. van Hummelen, A. L. Feldman, B. K. Link, A. J. Novak, J. R. Cerhan, T. M. Habermann, R. Siebert, A. Rosenwald, A. R. Thorner, M. L. Meyerson, T. R. Golub, R. Beroukhim, G. G. Wulf, G. Ott, S. J. Rodig, S. Monti, D. S. Neuberg, M. Loeffler, M. Pfreundschuh, L. Trumper, G. Getz, M. A. Shipp, Molecular subtypes of diffuse large B cell lymphoma are associated with distinct pathogenic mechanisms and outcomes. *Nat. Med.* **24**, 679–690 (2018).

13. R. Schmitz, G. W. Wright, D. W. Huang, C. A. Johnson, J. D. Phelan, J. Q. Wang, S. Roulland, M. Kasbekar, R. M. Young, A. L. Shaffer, D. J. Hodson, W. Xiao, X. Yu, Y. Yang, H. Zhao, W. Xu, X. Liu, B. Zhou, W. Du, W. C. Chan, E. S. Jaffe, R. D. Gascoyne, J. M. Connors, E. Campo, A. Lopez-Guillermo, A. Rosenwald, G. Ott, J. Delabie, L. M. Rimsza, K. Tay Kuang Wei, A. D. Zelenetz, J. P. Leonard, N. L. Bartlett, B. Tran, J. Shetty, Y. Zhao, D. R. Soppet, S. Pittaluga, W. H. Wilson, L. M. Staudt, Genetics and pathogenesis of diffuse large B-cell lymphoma. *N. Engl. J. Med.* **378**, 1396–1407 (2018).

14. L. Pasqualucci, R. Dalla-Favera, Genetics of diffuse large B-cell lymphoma. *Blood* **131**, 2307–2319 (2018).

15. H. Bouamar, S. Abbas, A. P. Lin, L. Wang, D. Jiang, K. N. Holder, M. C. Kinney, S. Hunicke-Smith, R. C. Aguiar, A capture-sequencing strategy identifies IRF8, EBF1, and APRIL as novel IGH fusion partners in B-cell lymphoma. *Blood* **122**, 726–733 (2013).

16. B. Chapuy, M. R. McKeown, C. Y. Lin, S. Monti, M. G. Roemer, J. Qi, P. B. Rahl, H. H. Sun, K. T. Yeda, J. G. Doench, E. Reichert, A. L. Kung, S. J. Rodig, R. A. Young, M. A. Shipp, J. E. Bradner, Discovery and characterization of super-enhancer-associated dependencies in diffuse large B cell lymphoma. *Cancer Cell* **24**, 777–790 (2013).

17. E. Bal, R. Kumar, M. Hadigol, A. B. Holmes, L. K. Hilton, J. W. Loh, K. Dreval, J. C. H. Wong, S. Vlasevska, C. Corinaldesi, R. K. Soni, K. Basso, R. D. Morin, H. Khiabanian, L. Pasqualucci, R. Dalla-Favera, Super-enhancer hypermutation alters oncogene expression in B cell lymphoma. *Nature* **607**, 808–815 (2022).

18. H. Wang, C. H. Lee, C. Qi, P. Tailor, J. Feng, S. Abbasi, T. Atsumi, H. C. Morse, III, IRF8 regulates B-cell lineage specification, commitment, and differentiation. *Blood* **112**, 4028–4038 (2008).

19. A. Yanez, H. S. Goodridge, Interferon regulatory factor 8 and the regulation of neutrophil, monocyte, and dendritic cell production. *Curr. Opin. Hematol.* **23**, 11–17 (2016).

20. T. Holtschke, J. Lohler, Y. Kanno, T. Fehr, N. Giese, F. Rosenbauer, J. Lou, K. P. Knobeloch, L. Gabriele, J. F. Waring, M. F. Bachmann, R. M. Zinkernagel, H. C. Morse III, K. Ozato, I. Horak,

Immunodeficiency and chronic myelogenous leukemia-like syndrome in mice with a targeted mutation of the ICSBP gene. *Cell* **87**, 307–317 (1996).

21. S. Salem, D. Langlais, F. Lefebvre, G. Bourque, V. Bigley, M. Haniffa, J. L. Casanova, D. Burk, A. Berghuis, K. M. Butler, T. R. Leahy, S. Hambleton, P. Gros, Functional characterization of the human dendritic cell immunodeficiency associated with the IRF8(K108E) mutation. *Blood* **124**, 1894–1904 (2014).

22. S. Salem, D. Salem, P. Gros, Role of IRF8 in immune cells functions, protection against infections, and susceptibility to inflammatory diseases. *Hum. Genet.* **139**, 707–721 (2020).

23. E. M. Mace, V. Bigley, J. T. Gunesch, I. K. Chinn, L. S. Angelo, M. A. Care, S. Maisuria, M. D. Keller, S. Togi, L. B. Watkin, D. F. LaRosa, S. N. Jhangiani, D. M. Muzny, A. Stray-Pedersen, Z. Coban Akdemir, J. B. Smith, M. Hernandez-Sanabria, D. T. Le, G. D. Hogg, T. N. Cao, A. G. Freud, E. P. Szymanski, S. Savic, M. Collin, A. J. Cant, R. A. Gibbs, S. M. Holland, M. A. Caligiuri, K. Ozato, S. Paust, G. M. Doody, J. R. Lupski, J. S. Orange, Biallelic mutations in IRF8 impair human NK cell maturation and function. *J. Clin. Invest.* **127**, 306–320 (2017).

24. S. Hambleton, S. Salem, J. Bustamante, V. Bigley, S. Boisson-Dupuis, J. Azevedo, A. Fortin, M. Haniffa, L. Ceron-Gutierrez, C. M. Bacon, G. Menon, C. Trouillet, D. McDonald, P. Carey, F. Ginhoux, L. Alsina, T. J. Zumwalt, X. F. Kong, D. Kumararatne, K. Butler, M. Hubeau, J. Feinberg, S. Al-Muhsen, A. Cant, L. Abel, D. Chaussabel, R. Doffinger, E. Talesnik, A. Grumach, A. Duarte, K. Abarca, D. Moraes-Vasconcelos, D. Burk, A. Berghuis, F. Geissmann, M. Collin, J. L. Casanova, P. Gros, IRF8 mutations and human dendritic-cell immunodeficiency. *N. Engl. J. Med.* **365**, 127–138 (2011).

25. A. Martinez, S. Pittaluga, M. Rudelius, T. Davies-Hill, D. Sebasigari, T. J. Fountaine, S. Hewitt, E. S. Jaffe, M. Raffeld, Expression of the interferon regulatory factor 8/ICSBP-1 in human reactive lymphoid tissues and B-cell lymphomas: A novel germinal center marker. *Am. J. Surg. Pathol.* **32**, 1190–1200 (2008).

26. C. H. Lee, M. Melchers, H. Wang, T. A. Torrey, R. Slota, C. F. Qi, J. Y. Kim, P. Lugar, H. J. Kong, L. Farrington, B. van der Zouwen, J. X. Zhou, V. Lougaris, P. E. Lipsky, A. C. Grammer, H. C. Morse III,

Regulation of the germinal center gene program by interferon (IFN) regulatory factor 8/IFN consensus sequence-binding protein. *J. Exp. Med.* **203**, 63–72 (2006).

27. J. Feng, H. Wang, D. M. Shin, M. Masiuk, C. F. Qi, H. C. Morse III, IFN regulatory factor 8 restricts the size of the marginal zone and follicular B cell pools. *J. Immunol.* **186**, 1458–1466 (2011).

28. S. Carotta, S. N. Willis, J. Hasbold, M. Inouye, S. H. Pang, D. Emslie, A. Light, M. Chopin, W. Shi, H. Wang, H. C. Morse III, D. M. Tarlinton, L. M. Corcoran, P. D. Hodgkin, S. L. Nutt, The transcription factors IRF8 and PU.1 negatively regulate plasma cell differentiation. *J. Exp. Med.* **211**, 2169–2181 (2014).

29. H. Wang, S. Jain, P. Li, J. X. Lin, J. Oh, C. Qi, Y. Gao, J. Sun, T. Sakai, Z. Naghashfar, S. Abbasi, A. L. Kovalchuk, S. Bolland, S. L. Nutt, W. J. Leonard, H. C. Morse, Transcription factors IRF8 and PU.1 are required for follicular B cell development and BCL6-driven germinal center responses. *Proc. Natl. Acad. Sci. U.S.A.* **116**, 9511–9520 (2019).

30. T. Tamura, P. Thotakura, T. S. Tanaka, M. S. Ko, K. Ozato, Identification of target genes and a unique cis element regulated by IRF-8 in developing macrophages. *Blood* **106**, 1938–1947 (2005).

31. K. Eagle, T. Harada, J. Kalfon, M. W. Perez, Y. Heshmati, J. Ewers, J. V. Koren, J. M. Dempster, G. Kugener, V. R. Paralkar, C. Y. Lin, N. V. Dharia, K. Stegmaier, S. H. Orkin, M. Pimkin, Transcriptional plasticity drives leukemia immune escape. *Blood Cancer Discov.* **3**, 394–409 (2022).

32. J. F. Marquis, O. Kapoustina, D. Langlais, R. Ruddy, C. R. Dufour, B. H. Kim, J. D. MacMicking, V. Giguere, P. Gros, Interferon regulatory factor 8 regulates pathways for antigen presentation in myeloid cells and during tuberculosis. *PLOS Genet.* **7**, e1002097 (2011).

33. D. M. Shin, C. H. Lee, H. C. Morse III, IRF8 governs expression of genes involved in innate and adaptive immunity in human and mouse germinal center B cells. *PLOS ONE* **6**, e27384 (2011).

34. N. Pishesha, T. J. Harmand, H. L. Ploegh, A guide to antigen processing and presentation. *Nat. Rev. Immunol.* **22**, 751–764 (2022).

35. B. N. Devaiah, D. S. Singer, CIITA and its dual roles in mhc gene transcription. *Front. Immunol.* **4**, 476 (2013).

36. S. E. Lacy, S. L. Barrans, P. A. Beer, D. Painter, A. G. Smith, E. Roman, S. L. Cooke, C. Ruiz, P. Glover, S. J. L. Van Hoppe, N. Webster, P. J. Campbell, R. M. Tooze, R. Patmore, C. Burton, S. Crouch, D. J. Hodson, Targeted sequencing in DLBCL, molecular subtypes, and outcomes: A haematological malignancy research network report. *Blood* **135**, 1759–1771 (2020).

37. A. Reddy, J. Zhang, N. S. Davis, A. B. Moffitt, C. L. Love, A. Waldrop, S. Leppa, A. Pasanen, L. Meriranta, M. L. Karjalainen-Lindsberg, P. Norgaard, M. Pedersen, A. O. Gang, E. Hogdall, T. B. Heavican, W. Lone, J. Iqbal, Q. Qin, G. Li, S. Y. Kim, J. Healy, K. L. Richards, Y. Fedoriw, L. Bernal-Mizrachi, J. L. Koff, A. D. Staton, C. R. Flowers, O. Paltiel, N. Goldschmidt, M. Calaminici, A. Clear, J. Gribben, E. Nguyen, M. B. Czader, S. L. Ondrejka, A. Collie, E. D. Hsi, E. Tse, R. K. H. Au-Yeung, Y. L. Kwong, G. Srivastava, W. W. L. Choi, A. M. Evens, M. Pilichowska, M. Sengar, N. Reddy, S. Li, A. Chadburn, L. I. Gordon, E. S. Jaffe, S. Levy, R. Rempel, T. Tzeng, L. E. Happ, T. Dave, D. Rajagopalan, J. Datta, D. B. Dunson, S. S. Dave, Genetic and functional drivers of diffuse large B cell lymphoma. *Cell* **171**, 481–494.e15 (2017).

38. D. Ennishi, A. Jiang, M. Boyle, B. Collinge, B. M. Grande, S. Ben-Neriah, C. Rushton, J. Tang, N. Thomas, G. W. Slack, P. Farinha, K. Takata, T. Miyata-Takata, J. Craig, A. Mottok, B. Meissner, S. Saberi, A. Bashashati, D. Villa, K. J. Savage, L. H. Sehn, R. Kridel, A. J. Mungall, M. A. Marra, S. P. Shah, C. Steidl, J. M. Connors, R. D. Gascoyne, R. D. Morin, D. W. Scott, Double-hit gene expression signature defines a distinct subgroup of germinal center B-cell-like diffuse large B-cell lymphoma. *J. Clin. Oncol.* **37**, 190–201 (2019).

39. R. D. Morin, K. Mungall, E. Pleasance, A. J. Mungall, R. Goya, R. D. Huff, D. W. Scott, J. Ding, A. Roth, R. Chiu, R. D. Corbett, F. C. Chan, M. Mendez-Lago, D. L. Trinh, M. Bolger-Munro, G. Taylor, A. Hadj Khodabakhshi, S. Ben-Neriah, J. Pon, B. Meissner, B. Woolcock, N. Farnoud, S. Rogic, E. L. Lim, N. A. Johnson, S. Shah, S. Jones, C. Steidl, R. Holt, I. Birol, R. Moore, J. M. Connors, R. D. Gascoyne, M. A. Marra, Mutational and structural analysis of diffuse large B-cell lymphoma using whole-genome sequencing. *Blood* **122**, 1256–1265 (2013).

40. M. C. J. Ma, S. Tadros, A. Bouska, T. Heavican, H. Yang, Q. Deng, D. Moore, A. Akhter, K. Hartert, N. Jain, J. Showell, S. Ghosh, L. Street, M. Davidson, C. Carey, J. Tobin, D. Perumal, J. M. Vose, M. A. Lunning, A. R. Sohani, B. J. Chen, S. Buckley, L. J. Nastoupil, R. E. Davis, J. R. Westin, N. H. Fowler, S. Parekh, M. Gandhi, S. Neelapu, D. Stewart, K. Bhalla, J. Iqbal, T. Greiner, S. J. Rodig, A. Mansoor, M. R. Green, Subtype-specific and co-occurring genetic alterations in B-cell non-Hodgkin lymphoma. *Haematologica* **107**, 690–701 (2022).

41. G. W. Wright, D. W. Huang, J. D. Phelan, Z. A. Coulibaly, S. Roulland, R. M. Young, J. Q. Wang, R. Schmitz, R. D. Morin, J. Tang, A. Jiang, A. Bagaev, O. Plotnikova, N. Kotlov, C. A. Johnson, W. H. Wilson, D. W. Scott, L. M. Staudt, A probabilistic classification tool for genetic subtypes of diffuse large B cell lymphoma with therapeutic implications. *Cancer Cell* **37**, 551–568.e14 (2020).

42. K. Krysiak, F. Gomez, B. S. White, M. Matlock, C. A. Miller, L. Trani, C. C. Fronick, R. S. Fulton, F. Kreisel, A. F. Cashen, K. R. Carson, M. M. Berrien-Elliott, N. L. Bartlett, M. Griffith, O. L. Griffith, T. A. Fehniger, Recurrent somatic mutations affecting B-cell receptor signaling pathway genes in follicular lymphoma. *Blood* **129**, 473–483 (2017).

43. H. Li, M. S. Kaminski, Y. Li, M. Yildiz, P. Ouillette, S. Jones, H. Fox, K. Jacobi, K. Saiya-Cork, D. Bixby, D. Lebovic, D. Roulston, K. Shedden, M. Sabel, L. Marentette, V. Cimmino, A. E. Chang, S. N. Malek, Mutations in linker histone genes HIST1H1 B, C, D, and E; OCT2 (POU2F2); IRF8; and ARID1A underlying the pathogenesis of follicular lymphoma. *Blood* **123**, 1487–1498 (2014).

44. R. I. Panea, C. L. Love, J. R. Shingleton, A. Reddy, J. A. Bailey, A. M. Moormann, J. A. Otieno, J. M. Ong'echa, C. I. Oduor, K. M. S. Schroeder, N. Masalu, N. J. Chao, M. Agajanian, M. B. Major, Y. Fedoriw, K. L. Richards, G. Rymkiewicz, R. R. Miles, B. Alobeid, G. Bhagat, C. R. Flowers, S. L. Ondrejka, E. D. Hsi, W. W. L. Choi, R. K. H. Au-Yeung, W. Hartmann, G. Lenz, H. Meyerson, Y. Y. Lin, Y. Zhuang, M. A. Luftig, A. Waldrop, T. Dave, D. Thakkar, H. Sahay, G. Li, B. C. Palus, V. Seshadri, S. Y. Kim, R. D. Gascoyne, S. Levy, M. Mukhopadyay, D. B. Dunson, S. S. Dave, The whole-genome landscape of Burkitt lymphoma subtypes. *Blood* **134**, 1598–1607 (2019).

45. J. Richter, M. Schlesner, S. Hoffmann, M. Kreuz, E. Leich, B. Burkhardt, M. Rosolowski, O. Ammerpohl, R. Wagener, S. H. Bernhart, D. Lenze, M. Szczepanowski, M. Paulsen, S. Lipinski, R. B. Russell, S. Adam-Klages, G. Apic, A. Claviez, D. Hasenclever, V. Hovestadt, N. Hornig, J. O. Korbel,

D. Kube, D. Langenberger, C. Lawerenz, J. Lisfeld, K. Meyer, S. Picelli, J. Pischimarov, B. Radlwimmer, T. Rausch, M. Rohde, M. Schilhabel, R. Scholtysik, R. Spang, H. Trautmann, T. Zenz, A. Borkhardt, H. G. Drexler, P. Moller, R. A. MacLeod, C. Pott, S. Schreiber, L. Trumper, M. Loeffler, P. F. Stadler, P. Lichter, R. Eils, R. Kuppers, M. Hummel, W. Klapper, P. Rosenstiel, A. Rosenwald, B. Brors, R. Siebert; ICGM MMML-Seq Project, Recurrent mutation of the ID3 gene in Burkitt lymphoma identified by integrated genome, exome and transcriptome sequencing. *Nat. Genet.* **44**, 1316–1320 (2012).

46. V. Vela, D. Juskevicius, S. Dirnhofer, T. Menter, A. Tzankov, Mutational landscape of marginal zone B-cell lymphomas of various origin: Organotypic alterations and diagnostic potential for assignment of organ origin. *Virchows Arch.* **480**, 403–413 (2022).

47. C. R. Escalante, E. Nistal-Villan, L. Shen, A. Garcia-Sastre, A. K. Aggarwal, Structure of IRF-3 bound to the PRDIII-I regulatory element of the human interferon-beta enhancer. *Mol. Cell* **26**, 703–716 (2007).

48. J. Jumper, R. Evans, A. Pritzel, T. Green, M. Figurnov, O. Ronneberger, K. Tunyasuvunakool, R. Bates, A. Zidek, A. Potapenko, A. Bridgland, C. Meyer, S. A. A. Kohl, A. J. Ballard, A. Cowie, B. Romera-Paredes, S. Nikolov, R. Jain, J. Adler, T. Back, S. Petersen, D. Reiman, E. Clancy, M. Zielinski, M. Steinegger, M. Pacholska, T. Berghammer, S. Bodenstein, D. Silver, O. Vinyals, A. W. Senior, K. Kavukcuoglu, P. Kohli, D. Hassabis, Highly accurate protein structure prediction with AlphaFold. *Nature* **596**, 583–589 (2021).

49. B. Z. Levi, S. Hashmueli, M. Gleit-Kielmanowicz, A. Azriel, D. Meraro, ICSBP/IRF-8 transactivation: A tale of protein-protein interaction. *J. Interferon Cytokine Res.* **22**, 153–160 (2002).

50. Y. Xu, L. Jiang, J. Fang, R. Fang, H. C. Morse III, G. Ouyang, J. X. Zhou, Loss of IRF8 inhibits the growth of diffuse large B-cell lymphoma. *J. Cancer* **6**, 953–961 (2015).

51. M. A. Smith, G. Wright, J. Wu, P. Tailor, K. Ozato, X. Chen, S. Wei, J. F. Piskurich, J. P. Ting, K. L. Wright, Positive regulatory domain I (PRDM1) and IRF8/PU.1 counter-regulate MHC class II transactivator (CIITA) expression during dendritic cell maturation. *J. Biol. Chem.* **286**, 7893–7904 (2011).

52. V. Vila-del Sol, C. Punzon, M. Fresno, IFN-gamma-induced TNF-alpha expression is regulated by interferon regulatory factors 1 and 8 in mouse macrophages. *J. Immunol.* **181**, 4461–4470 (2008).

53. J. Liao, Y. Luan, Z. Ren, X. Liu, D. Xue, H. Xu, Z. Sun, K. Yang, H. Peng, Y. X. Fu, Converting lymphoma cells into potent antigen-presenting cells for interferon-induced tumor regression. *Cancer Immunol. Res.* **5**, 560–570 (2017).

54. B. M. Roy, D. V. Zhukov, J. A. Maynard, Flanking residues are central to DO11.10 T cell hybridoma stimulation by ovalbumin 323-339. *PLOS ONE* **7**, e47585 (2012).

55. F. Miyagawa, J. Gutermuth, H. Zhang, S. I. Katz, The use of mouse models to better understand mechanisms of autoimmunity and tolerance. *J. Autoimmun.* **35**, 192–198 (2010).

56. M. de Charette, R. Houot, Hide or defend, the two strategies of lymphoma immune evasion: Potential implications for immunotherapy. *Haematologica* **103**, 1256–1268 (2018).

57. C. D. Scharer, N. M. Choi, B. G. Barwick, P. Majumder, S. Lohsen, J. M. Boss, Genome-wide CIITA-binding profile identifies sequence preferences that dictate function versus recruitment. *Nucleic Acids Res.* **43**, 3128–3142 (2015).

58. B. G. Nixon, F. Kuo, L. Ji, M. Liu, K. Capistrano, M. Do, R. A. Franklin, X. Wu, E. R. Kansler, R. M. Srivastava, T. A. Purohit, A. Sanchez, L. Vuong, C. Krishna, X. Wang, H. C. Morse Iii, J. J. Hsieh, T. A. Chan, K. M. Murphy, J. J. Moon, A. A. Hakimi, M. O. Li, Tumor-associated macrophages expressing the transcription factor IRF8 promote T cell exhaustion in cancer. *Immunity* **55**, 2044–2058.e5 (2022).

59. D. Aran, Z. Hu, A. J. Butte, xCell: Digitally portraying the tissue cellular heterogeneity landscape. *Genome Biol.* **18**, 220 (2017).

60. D. Krijgsman, M. Hokland, P. J. K. Kuppen, The role of natural killer T cells in cancer-a phenotypical and functional approach. *Front. Immunol.* **9**, 367 (2018).

61. M. Swiecki, M. Colonna, The multifaceted biology of plasmacytoid dendritic cells. *Nat. Rev. Immunol.* **15**, 471–485 (2015).

62. G. Oliveira, C. J. Wu, Dynamics and specificities of T cells in cancer immunotherapy. *Nat. Rev. Cancer* **23**, 295–316 (2023).

63. B. J. Sworder, D. M. Kurtz, S. K. Alig, M. J. Frank, N. Shukla, A. Garofalo, C. W. Macaulay, M. Shahrokh Esfahani, M. N. Olsen, J. Hamilton, H. Hosoya, M. Hamilton, J. Y. Spiegel, J. H. Baird, T. Sugio, M. Carleton, A. F. M. Craig, S. F. Younes, B. Sahaf, N. D. Sheybani, J. G. Schroers-Martin, C. L. Liu, J. S. Oak, M. C. Jin, S. Beygi, A. Huttmann, C. Hanoun, U. Duhrsen, J. R. Westin, M. S. Khodadoust, Y. Natkunam, R. G. Majzner, C. L. Mackall, M. Diehn, D. B. Miklos, A. A. Alizadeh, Determinants of resistance to engineered T cell therapies targeting CD19 in large B cell lymphomas. *Cancer Cell* **41**, 210–225.e5 (2023).

64. A. M. Newman, C. L. Liu, M. R. Green, A. J. Gentles, W. Feng, Y. Xu, C. D. Hoang, M. Diehn, A. A. Alizadeh, Robust enumeration of cell subsets from tissue expression profiles. *Nat. Methods* **12**, 453–457 (2015).

65. E. Becht, N. A. Giraldo, L. Lacroix, B. Buttard, N. Elarouci, F. Petitprez, J. Selves, P. Laurent-Puig, C. Sautes-Fridman, W. H. Fridman, A. de Reynies, Estimating the population abundance of tissue-infiltrating immune and stromal cell populations using gene expression. *Genome Biol.* **17**, 218 (2016).

66. N. M. Adams, C. M. Lau, X. Fan, M. Rapp, C. D. Geary, O. E. Weizman, C. Diaz-Salazar, J. C. Sun, Transcription factor IRF8 orchestrates the adaptive natural killer cell response. *Immunity* **48**, 1172–1182.e6 (2018).

67. J. C. Sun, S. Madera, N. A. Bezman, J. N. Beilke, M. H. Kaplan, L. L. Lanier, Proinflammatory cytokine signaling required for the generation of natural killer cell memory. *J. Exp. Med.* **209**, 947–954 (2012).

68. G. Schiavoni, F. Mattei, P. Sestili, P. Borghi, M. Venditti, H. C. Morse III, F. Belardelli, L. Gabriele, ICSBP is essential for the development of mouse type I interferon-producing cells and for the generation and activation of CD8alpha^{+} dendritic cells. *J. Exp. Med.* **196**, 1415–1425 (2002).

69. Z. Shulman, A. D. Gitlin, S. Targ, M. Jankovic, G. Pasqual, M. C. Nussenzweig, G. D. Victora, T follicular helper cell dynamics in germinal centers. *Science* **341**, 673–677 (2013).

70. E. Dheilly, E. Battistello, N. Katanayeva, S. Sungalee, J. Michaux, G. Duns, S. Wehrle, J. Sordet-Dessimoz, M. Mina, J. Racle, P. Farinha, G. Coukos, D. Gfeller, A. Mottok, R. Kridel, B. E. Correia, C. Steidl, M. Bassani-Sternberg, G. Ciriello, V. Zoete, E. Oricchio, Cathepsin S regulates antigen processing and T cell activity in non-hodgkin lymphoma. *Cancer Cell* **37**, 674–689.e12 (2020).

71. M. Boice, D. Salloum, F. Mourcin, V. Sanghvi, R. Amin, E. Oricchio, M. Jiang, A. Mottok, N. Denis-Lagache, G. Ciriello, W. Tam, J. Teruya-Feldstein, E. de Stanchina, W. C. Chan, S. N. Malek, D. Ennishi, R. J. Brentjens, R. D. Gascoyne, M. Cogne, K. Tarte, H. G. Wendel, Loss of the HVEM tumor suppressor in lymphoma and restoration by modified CAR-T cells. *Cell* **167**, 405–418.e13 (2016).

72. N. Gutierrez-Melo, D. Baumjohann, T follicular helper cells in cancer. *Trends Cancer* **9**, 309–325 (2023).

73. M. Ortega, H. Bhatnagar, A. P. Lin, L. Wang, J. C. Aster, H. Sill, R. C. Aguiar, A microRNA-mediated regulatory loop modulates NOTCH and MYC oncogenic signals in B- and T-cell malignancies. *Leukemia* **29**, 968–976 (2015).

74. A. P. Lin, Z. Qiu, P. Ethiraj, B. Sasi, C. Jaafar, D. Rakheja, R. C. T. Aguiar, MYC, mitochondrial metabolism and O-GlcNAcylation converge to modulate the activity and subcellular localization of DNA and RNA demethylases. *Leukemia* **36**, 1150–1159 (2022).

75. A. N. Suhasini, L. Wang, K. N. Holder, A. P. Lin, H. Bhatnagar, S. W. Kim, A. W. Moritz, R. C. T. Aguiar, A phosphodiesterase 4B-dependent interplay between tumor cells and the microenvironment regulates angiogenesis in B-cell lymphoma. *Leukemia* **30**, 617–626 (2016).

76. A. P. Lin, S. Abbas, S. W. Kim, M. Ortega, H. Bouamar, Y. Escobedo, P. Varadarajan, Y. Qin, J. Sudderth, E. Schulz, A. Deutsch, S. Mohan, P. Ulz, P. Neumeister, D. Rakheja, X. Gao, A. Hinck, S. T. Weintraub, R. J. DeBerardinis, H. Sill, P. L. Dahia, R. C. Aguiar, D2HGDH regulates alpha-ketoglutarate levels and dioxygenase function by modulating IDH2. *Nat. Commun.* **6**, 7768 (2015).

77. B. Sasi, P. Ethiraj, J. Myers, A. P. Lin, S. Jiang, Z. Qiu, K. N. Holder, R. C. T. Aguiar, Regulation of PD-L1 expression is a novel facet of cyclic-AMP-mediated immunosuppression. *Leukemia* **35**, 1990–2001 (2021).

78. J. D. Cooney, A. P. Lin, D. Jiang, L. Wang, A. N. Suhasini, J. Myers, Z. Qiu, A. Wolfler, H. Sill, R. C. T. Aguiar, Synergistic targeting of the regulatory and catalytic subunits of PI3Kδ in mature B-cell malignancies. *Clin. Cancer Res.* **24**, 1103–1113 (2018).

79. D. C. Koboldt, Q. Zhang, D. E. Larson, D. Shen, M. D. McLellan, L. Lin, C. A. Miller, E. R. Mardis, L. Ding, R. K. Wilson, VarScan 2: Somatic mutation and copy number alteration discovery in cancer by exome sequencing. *Genome Res.* **22**, 568–576 (2012).

80. E. Cerami, J. Gao, U. Dogrusoz, B. E. Gross, S. O. Sumer, B. A. Aksoy, A. Jacobsen, C. J. Byrne, M. L. Heuer, E. Larsson, Y. Antipin, B. Reva, A. P. Goldberg, C. Sander, N. Schultz, The cBio cancer genomics portal: An open platform for exploring multidimensional cancer genomics data. *Cancer Discov.* **2**, 401–404 (2012).

81. Z. Qiu, A. P. Lin, S. Jiang, S. M. Elkashef, J. Myers, S. Srikantan, B. Sasi, J. Z. Cao, L. A. Godley, D. Rakheja, Y. Lyu, S. Zheng, M. Madesh, Y. Shiio, P. L. M. Dahia, R. C. T. Aguiar, MYC regulation of D2HGDH and L2HGDH influences the epigenome and epitranscriptome. *Chem. Biol.* **27**, 538–550.e7 (2020).

82. H. Bouamar, D. Jiang, L. Wang, A. P. Lin, M. Ortega, R. C. Aguiar, MicroRNA 155 control of p53 activity is context dependent and mediated by Aicda and Socs1. *Mol. Cell. Biol.* **35**, 1329–1340 (2015).

83. E. Bustos-Moran, N. Blas-Rus, A. Alcaraz-Serna, S. Iborra, J. Gonzalez-Martinez, M. Malumbres, F. Sanchez-Madrid, Aurora A controls $CD8^+$ T cell cytotoxic activity and antiviral response. *Sci. Rep.* **9**, 2211 (2019).

84. Z. Qiu, K. N. Holder, A. P. Lin, J. Myers, S. Jiang, K. M. Gorena, M. C. Kinney, R. C. T. Aguiar, Generation and characterization of the Eμ-Irf8 mouse model. *Cancer Genet.* **245**, 6–16 (2020).

85. P. Ethiraj, B. Sasi, K. N. Holder, A. P. Lin, Z. Qiu, C. Jaafar, A. Elkhalili, P. Desai, A. Saksena, J. P. Ritter, R. C. T. Aguiar, Cyclic-AMP signalling, MYC and hypoxia-inducible factor 1α intersect to regulate angiogenesis in B-cell lymphoma. *Br. J. Haematol.* **198**, 349–359 (2022).

86. D. Jiang, R. C. Aguiar, MicroRNA-155 controls RB phosphorylation in normal and malignant B lymphocytes via the noncanonical TGF-β1/SMAD5 signaling module. *Blood* **123**, 86–93 (2014).

87. P. Emsley, K. Cowtan, Coot: Model-building tools for molecular graphics. *Acta Crystallogr. D Biol. Crystallogr.* **60**, 2126–2132 (2004).

88. J. G. Lohr, P. Stojanov, S. L. Carter, P. Cruz-Gordillo, M. S. Lawrence, D. Auclair, C. Sougnez, B. Knoechel, J. Gould, G. Saksena, K. Cibulskis, A. McKenna, M. A. Chapman, R. Straussman, J. Levy, L. M. Perkins, J. J. Keats, S. E. Schumacher, M. Rosenberg; Multiple Myeloma Research Consortium, G. Getz, T. R. Golub, Widespread genetic heterogeneity in multiple myeloma: Implications for targeted therapy. *Cancer Cell* **25**, 91–101 (2014).

89. V. Quesada, L. Conde, N. Villamor, G. R. Ordonez, P. Jares, L. Bassaganyas, A. J. Ramsay, S. Bea, M. Pinyol, A. Martinez-Trillos, M. Lopez-Guerra, D. Colomer, A. Navarro, T. Baumann, M. Aymerich, M. Rozman, J. Delgado, E. Gine, J. M. Hernandez, M. Gonzalez-Diaz, D. A. Puente, G. Velasco, J. M. Freije, J. M. Tubio, R. Royo, J. L. Gelpi, M. Orozco, D. G. Pisano, J. Zamora, M. Vazquez, A. Valencia, H. Himmelbauer, M. Bayes, S. Heath, M. Gut, I. Gut, X. Estivill, A. Lopez-Guillermo, X. S. Puente, E. Campo, C. Lopez-Otin, Exome sequencing identifies recurrent mutations of the splicing factor SF3B1 gene in chronic lymphocytic leukemia. *Nat. Genet.* **44**, 47–52 (2011).

90. D. A. Landau, E. Tausch, A. N. Taylor-Weiner, C. Stewart, J. G. Reiter, J. Bahlo, S. Kluth, I. Bozic, M. Lawrence, S. Bottcher, S. L. Carter, K. Cibulskis, D. Mertens, C. L. Sougnez, M. Rosenberg, J. M. Hess, J. Edelmann, S. Kless, M. Kneba, M. Ritgen, A. Fink, K. Fischer, S. Gabriel, E. S. Lander, M. A. Nowak, H. Dohner, M. Hallek, D. Neuberg, G. Getz, S. Stilgenbauer, C. J. Wu, Mutations driving CLL and their evolution in progression and relapse. *Nature* **526**, 525–530 (2015).

91. X. S. Puente, S. Bea, R. Valdes-Mas, N. Villamor, J. Gutierrez-Abril, J. I. Martin-Subero, M. Munar, C. Rubio-Perez, P. Jares, M. Aymerich, T. Baumann, R. Beekman, L. Belver, A. Carrio, G. Castellano, G. Clot, E. Colado, D. Colomer, D. Costa, J. Delgado, A. Enjuanes, X. Estivill, A. A. Ferrando, J. L. Gelpi, B. Gonzalez, S. Gonzalez, M. Gonzalez, M. Gut, J. M. Hernandez-Rivas, M. Lopez-Guerra, D. Martin-Garcia, A. Navarro, P. Nicolas, M. Orozco, A. R. Payer, M. Pinyol, D. G. Pisano, D. A. Puente, A. C. Queiros, V. Quesada, C. M. Romeo-Casabona, C. Royo, R. Royo, M. Rozman, N. Russinol, I. Salaverria, K. Stamatopoulos, H. G. Stunnenberg, D. Tamborero, M. J. Terol, A. Valencia, N. Lopez-Bigas, D. Torrents, I. Gut, A. Lopez-Guillermo, C. Lopez-Otin, E. Campo, Non-coding recurrent mutations in chronic lymphocytic leukaemia. *Nature* **526**, 519–524 (2015).
